# Favorable Conditions for Genomic Evaluation to Outperform Classical Pedigree Evaluation Highlighted by a Proof-of-Concept Study in Poplar

**DOI:** 10.3389/fpls.2020.581954

**Published:** 2020-10-28

**Authors:** Marie Pégard, Vincent Segura, Facundo Muñoz, Catherine Bastien, Véronique Jorge, Leopoldo Sanchez

**Affiliations:** ^1^BioForA, INRA, ONF, Orléans, France; ^2^AGAP, Univ Montpellier, CIRAD, INRAE, Institut Agro, Montpellier, France

**Keywords:** black poplar, genomic evaluation, marker density, degraded phenotypes, non-additive effects, multi-trait, intra-family selection, breeding scheme

## Abstract

Forest trees like poplar are particular in many ways compared to other domesticated species. They have long juvenile phases, ongoing crop-wild gene flow, extensive outcrossing, and slow growth. All these particularities tend to make the conduction of breeding programs and evaluation stages costly both in time and resources. Perennials like trees are therefore good candidates for the implementation of genomic selection (GS) which is a good way to accelerate the breeding process, by unchaining selection from phenotypic evaluation without affecting precision. In this study, we tried to compare GS to pedigree-based traditional evaluation, and evaluated under which conditions genomic evaluation outperforms classical pedigree evaluation. Several conditions were evaluated as the constitution of the training population by cross-validation, the implementation of multi-trait, single trait, additive and non-additive models with different estimation methods (G-BLUP or weighted G-BLUP). Finally, the impact of the marker densification was tested through four marker density sets. The population under study corresponds to a pedigree of 24 parents and 1,011 offspring, structured into 35 full-sib families. Four evaluation batches were planted in the same location and seven traits were evaluated on 1 and 2 years old trees. The quality of prediction was reported by the accuracy, the Spearman rank correlation and prediction bias and tested with a cross-validation and an independent individual test set. Our results show that genomic evaluation performance could be comparable to the already well-optimized pedigree-based evaluation under certain conditions. Genomic evaluation appeared to be advantageous when using an independent test set and a set of less precise phenotypes. Genome-based methods showed advantages over pedigree counterparts when ranking candidates at the within-family levels, for most of the families. Our study also showed that looking at ranking criteria as Spearman rank correlation can reveal benefits to genomic selection hidden by biased predictions.

## 1. Background

Forest tree species of interest for domestication like poplar are particular in many ways compared to other domesticated species, notably when it comes to breeding. Among the various particularities, forest trees have long juvenile phases, ongoing crop-wild gene flow, and extensive outcrossing (Miller and Gross, 2011). All of these hamper the process of “*controlled*” recombination by the breeder. Slow growth and cumbersomeness typical of trees do not facilitate either the conduction of breeding programs, notably with evaluation stages being costly both in time and resources. One of the poplar's particularities is clonality or the possibility of asexual reproduction, which is a powerful tool in evaluation and operational breeding (Bisognin, 2011). However, benefits rarely go hand in hand with simplicity. Typically for developing a new poplar variety, a first year is used for mating and seedling growth in nurseries. A second year is used to propagate the cuttings and install the experiments using a statistical design to do evaluations in different environments, and many subsequent years pass before we can assess genotype-by-environment (G × E) interactions, or late maturation traits like wood quality. Selection in poplars proceeds typically via independent level stages (independent culling levels), with early stages involving screening for fast-growing, disease-resistant individuals from large numbers of candidates. Late stages focus on a reduced remainder to select on final growth, architecture, disease resistance, and wood properties. This has been so far operationally efficient considering the constraints imposed by the particularities of trees, but it remains time consuming and lacks precision at the early stages.

For previous and additional reasons, perennials like trees are good candidates for the implementation of genomic selection (GS) (Muranty et al., 2014). GS can potentially accelerate the breeding process, by unchaining selection from phenotypic evaluation without affecting precision (Meuwissen et al., 2001). When applied early at the seedling stage, GS could potentially save evaluation resources and reduce the time required for evaluation of late maturation traits. GS involves ranking and selecting individuals by using a genome-wide marker set and prediction models calibrated previously in a training set. GS has been made possible thanks to easy access to cheap genotyping data, and to recent developments in evaluation methodology (de los Campos et al., 2009). Recent studies of GS in forest trees were conducted on several species: eucalypts (Resende et al., 2012b; Müller et al., 2017; Tan et al., 2017, 2018; Cappa et al., 2019; Ballesta et al., 2020), spruce (Gamal El-Dien et al., 2015, 2016; Ratcliffe et al., 2015; Lenz et al., 2017, 2020; Chen et al., 2018; Chamberland et al., 2020), pines (Resende et al., 2012a; de Almeida Filho et al., 2016; Ratcliffe et al., 2017; Gianola and Fernando, 2020; Ukrainetz and Mansfield, 2020), and rubber trees (Cros et al., 2019; Souza et al., 2019). Given the differences among forest species in general, and between their breeding programs in particular, assessments of GS feasibility at a case-by-case basis are often desirable.

According to Hayes et al. (2009), several parameters are involved in genomic evaluation accuracy. First, the extent of linkage disequilibrium in the population, which is linked to the effective population size, affects the accuracy of genomic prediction. Linkage facilitates the use of markers as *proxies* of unknown QTLs in estimating genetic effects. The required marker density is directly dictated by the extent of linkage disequilibrium: the lower the linkage disequilibrium, the higher the number of required markers (Grattapaglia and Resende, 2011; Wientjes et al., 2013). The second parameter of importance for accuracy is the composition of the training set. Such a set must be representative of the candidates for which a prediction is required. Several studies developed methods to optimize the composition of the training set (Rincent et al., 2012; Akdemir et al., 2015; Isidro et al., 2015). The third parameter is trait genetic architecture, usually unknown or poorly understood, but that has an influence on the performances of the different evaluation methods (Wimmer et al., 2013). Some evaluation methods, such as those using some efficient strategy to focus only on relevant variables like the family of bayesian methods, appear to be more efficient with traits with fairly uneven distributions of gene effects. Other methods with less stringent *a priori* on the distribution of gene effects work generally well with highly polygenic traits, like G-BLUP. Other modeling approaches intent to capture the underlying complexity of genetic architectures, by including non-additive effects like dominance and epistatic interactions (Toro and Varona, 2010; Su et al., 2012; Vitezica et al., 2013, 2017; Muñoz et al., 2014; Martini et al., 2017), and by considering multiple correlated traits. The latter have not been often used, despite some promising simulation studies (Calus and Veerkamp, 2011; Guo et al., 2014), empirical studies (Jia and Jannink, 2012), and the known fact from classical evaluation that genetic correlations can back accuracies of poorly heritable traits or those harboring many missing values in the dataset (Gilmour et al., 2009).

In the present work, we intended to benefit from the large corpus of knowledge already established around the concept of GS to carry out a proof-of-concept study on the feasibility of the methodology in the context of the black poplar breeding program in France. Black poplar is the leading Eurasian species of riparian forest, with a wide distribution area, and contributing as a parent together with *Populus deltoides* to one of the most widely used hybrid (*Populus* × *canadensis*) tree in the wood industry. This study is the first GS study for a *Populus* species. One of the main objectives of the study was to compare GS to pedigree-based traditional evaluation, by assessing different modeling options including non-additive genetic effects and multiple-trait evaluation. The study also considered the role of marker densification in the performance of GS, by benefiting from a recent imputation study (Pegard et al., 2018). The potential benefits of shortening the breeding cycle, although of importance, were not evaluated but only discussed in present work, because of the relatively late sexual maturity in the species. Finally, the design of the calibration and validation sets was taken into account as an additional factor in the comparison. Globally, the study intended to identify the situations in which GS could be a feasible option for poplar, and also the assessments required to reveal any eventual advantage.

## 2. Materials and Methods

### 2.1. Plant Material

The population under study corresponds to a pedigree of 24 parents and 1,011 offspring, structured into 35 full-sib cohorts, and involving a 4 by 4 factorial mating design together with a series of multiple pair-mating designs (Pegard et al., 2018). Most of the parents were sampled from natural populations or were high-performance trees already used in the breeding program. The population corresponds therefore to the offsprings of these individuals obtained by controlled crosses. The effective population size was estimated to be 12 from coancestry matrices (Caballero, 2000) computed from pedigree corrected by marker information. Family size ranged from 10 to 118, with an average of 26 individuals per family. Pedigree description and distribution of family sizes are available in Table 1.

**Table 1:** Description of the pedigree and distribution of family sizes after correction of the pedigree from the marker information.

**Father**	**SAN-GIORGIO**												
**Mother**	**SRZ**	**BDG**	**71077-308**	**92510-1**	**72145-007**	**72131-017**	**73182-009**	**73193-056**	**72131-036**	**3824-3**	**71034-2-406**	**72146-11**	**Total**
VGN	55	57	54	32		34	14	30					276
71041-3-402		28	11	17	30		25						111
71072-501	25	28	29										82
SSC	15	20	20	22									77
71040					24	20							44
662200037					25	32	118	31					206
73193-089						22	20	18					60
662200216							31		19				50
71069-914										22			22
73193-091										21		30	51
H480										13	19		32
Total	95	133	114	71	79	108	208	79	19	56	19	30	1,011

*Colors correspond to experimental field trials: green for the trial conducted in 2000/2001, pink for the trial conducted in 2012/2013, blue for the trial conducted in 2014/2016, and orange for the trial conducted in 2017/2018. These latter individuals represent two parental females (underlined codes) are progenies of VGN and BDG and were subsequently used as parent for the multiple pair mating*.

### 2.2. Phenotyping

Field evaluations corresponded to four different experimental trials. All four experimental trials were planted in the same location (47° 37'59” N, 1° 49'59” W, Guéméné-Penfao, France) with small variations in plot orientation and with common genotypes as controls across experimental trials (Supplementary Table 1). The first experimental trial (2000 and 2001) involved the factorial mating design with a total of 14 families and 413 offspring phenotyped. In second and third experimental trials, 126 individuals in 6 families and 105 from 5 families were phenotyped (2012/2013 and 2014/2016, respectively). Finally, in order to reinforce the connectivity between the different experimental trials, 10 additional full-sib families with some parents already in use in previous experimental trials were added in 2015 and phenotyped in 2017/2018. In total, 367 individuals were phenotyped in this last batch. At their respective time-frames, all 1,011 offspring and the 24 parents were vegetatively propagated, and field evaluated in separate experiments according to the same six randomized complete block design.

Phenotyping involved seven different measurements over different years (2000/2001, 2012/2013, 2014/2016, and 2017/2018), and for five different traits. Growth was assessed as stem circumference and tree height. Stem circumference at 1 m was considered for the second year (circ2). Height was assessed with a graduated rod after 1 (height1) and 2 years of growth (height2). Mean branching angle was scored on proleptic branches at the age of 2 years with a 1–4 scoring scale (angbranch; score 1 : 0–30° from the horizontal; score 2: 30–40°; score 3: 40–55°; score 4: >55°). The scale for angbranch was calibrated in such a way that resulting measures in the same population of reference resulted in phenotyping distributions being close to normality. Rust resistance was assessed with a 1 (no symptom) to 9 (generalized symptoms) scale (Legionnet et al., 1999) at year 1 (rust1) and year 2 (rust2). Budburst phenology of the stem terminal bud was evaluated by measuring its kinetics (every 3 or 5 days from March to April) with a 0–5 scale, where stage 0 corresponded to a completely closed bud while stage 5 corresponded to the initiation of stem internode elongation (Castellani et al., 1967). A local polynomial regression model was fitted between stages and dates for each individual and this model was further used to predict the date in Julian days at which the terminal bud was at stage 3 and in order to assess individual susceptibility to late frosts (Howe et al., 2000). As a result of such fitting for budburst, distributions were continuous and close to normality.

All seven phenotypes were independently adjusted to field micro-environmental heterogeneity with the breedR package [Muñoz and Sanchez, 2018, implemented in R3.3.1 platform (R Core Team, 2018)]. We used an individual-tree mixed model over all four experimental trials, comprising all available information with genotyped and non-genotyped individuals (not included in this study) according to a single-step formulation (mixture of pedigree relationship matrix for non-genotyped individuals and genomic equivalent for genotyped individuals) (Legarra et al., 2009). A random effect capturing spatial heterogeneity at individual level within trials was fitted thanks to the use of a bi-splines surface covering row and column axes (Cappa and Cantet, 2007; Cappa et al., 2015). Such a surface was nested within each evaluation experimental trial. Bi-splines were anchored at a given number of knots for rows and columns, with higher numbers increasing the roughness of the surfaces and lower numbers giving extra smoothness. Knot numbers were optimized by an automated grid search based on the Akaike information criterion (Akaike 1974) provided by breedR. The use of all available information in field trials, including non-genotyped individuals, minimized the occurrence of gaps in the surfaces and facilitated the prediction of accurate micro-environmental individual effects across the experiment. The fact of using common genotypes across trials (see Supplementary Table 1) and the use of genomic and pedigree relatedness in the mixed model facilitated the adjustment across trials. The micro-environmental individual effect was subtracted from the observed phenotype to obtain a spatially adjusted individual phenotype. A clonal mean of spatially adjusted phenotypes was calculated for each trait and used as raw phenotypes for the rest of the study (hereafter adjusted clonal mean). As a default option, data from all blocks (6 Blocs) were used as input to the model. The same model was fitted to data from only three of the blocks (blocks 1, 3, and 5 called 3 Blocs model afterwards), to assess prediction quality with a less precise phenotype. All measurements were tested for deviations from normality by a randomized Q-Q plot.

### 2.3. Genotyping

All 1,033 individuals in this population (22 parents, 2 being both parents and offspring, and 1,009 offspring) were genotyped using the Populus nigra 12K custom Infinium Bead-Chip (Illumina, San Diego, CA) (Faivre-Rampant et al., 2016). Additionally, 43 individuals were sequenced, including an extra founder that was identified as one of the grandparents in the pedigree. Among the remaining 42 sequenced, there were 22 parents, 14 progenies, and six unrelated individuals from natural populations. Progenies were chosen in such a way that all parents had at least one offspring with its genome sequenced. The set of unrelated individuals were used to assess the imputation ability under challenging conditions. In a previous study (Pegard et al., 2018) genotype imputation from 7K (effective SNPs out of 12K in array) to 1,466,586 SNPs was performed attaining imputation qualities higher than 0.84 per individual, and evaluated by a leave-one-out cross-validation scheme (CV). Resulting imputation was used in the present study to constitute alternative sets of selected markers for genotyping. For quality assessment and selection of the marker sets, we used the proportion of alleles correctly imputed by genomic position across individuals (Props), and Props corrected by the probability of correct imputation by chance (Badke et al., 2013; cProps). Among the imputed SNPs, we selected those with Props higher than 0.90, with cProps higher than 0.60 and a minor allele frequency (MAF) higher than 0.05, to obtain a set of 249,805 SNPs (250K). That latter set comprised the total of the 7K from the chip. We selected two alternative smaller marker sets: 50K (with 50,565 SNPs), and 7K_homo (with 7,048 SNPs) where coverage and homogeneity of density was optimized over the original 7K array. These two sets were composed by selecting, respectively 1 SNPs every 1,000 or 50,000 bp out of the 250K set. Whenever more than one candidate SNPs were available for the same window, we selected the one that had the highest values of Props and cProps.

### 2.4. Models

We estimated variance components and heritabilities with the complete data set and single trait models, and genetic correlations with a genomic multiple-trait model (GBLUP). The Akaike Information Criterion (AIC) was used to assess for each given trait the quality of each model. Two alternative methods were used to calculate genomic estimated breeding values for each trait: the best linear unbiased prediction based on genomic information (GBLUP) (Whittaker et al., 2000; Meuwissen et al., 2001), and the weighted GBLUP (wGBLUP; Legarra et al., 2009; Zhang et al., 2016). They were all compared to the best linear unbiased prediction based on pedigree information (PBLUP) (Henderson, 1975). The models for GBLUP (and PBLUP) using matrix notation for additive and non-additive effects were given by:

(1) $$\begin{array}{r} y=B\beta +Zu+\varepsilon \end{array}$$

(2) $$\begin{array}{r} y=B\beta +Zu+Wd+\varepsilon \end{array}$$

where *y* was the adjusted clonal mean, β a vector of fixed effects, *u* the vector of random additive effects following N(0,$G\sigma_{a}^{2}$) with $\sigma_{a}^{2}$ the additive variance and G (or A in PBLUP) the relationship matrix, *d* was the vector of random dominance effects following N(0,$D\sigma_{d}^{2}$) with $\sigma_{d}^{2}$ the dominance variance and *D* the dominance relationship matrix, ε the vector of residual effects following N(0,$I\sigma_{e}^{2}$) with $\sigma_{e}^{2}$ the residual variance. The design matrix *B* contains the values of the covariables with fixed effects and *Z*, *W*, and *I* are indicator matrices relating the clonal mean to the random effects. The methods used to obtain the relationship matrices are explained in the next section. The PBLUP and GBLUP single-trait models as well as the multi-trait models were fitted with the R package breedR (Muñoz and Sanchez, 2018). All the analyses are summarized in Table 2.

**Table 2:** Combination of models and marker sets tested.

**Methods**	**ADD**	**ADD + DOM**	**MultiTrait**	**SNP set**
P-BLUP	Yes	Yes	Yes	None
P-BLUPcor	Yes	Yes	Yes	None
GBLUP	Yes	Yes	Yes	7K
	Yes	Yes	No	50K
	Yes	Yes	No	100K
	Yes	Yes	No	250K
wGBLUP	Yes	Yes	No	7K
	Yes	Yes	No	50K
	Yes	Yes	No	100K
	Yes	Yes	No	250K
BayesCpi	Yes	No	No	7K
	Yes	No	No	50K
	Yes	No	No	100K
	No	No	No	250K

### 2.5. Relationship Matrix Estimation

The ARM (additive relationship matrix) was built from the known pedigree at the moment of the controlled crossings, and denoted hereafter as *A*. However, a preliminary marker assessment in this study showed that there were errors in the pedigree. Pedigree was corrected based on these results and a new reconstructed ARM was obtained, denoted hereafter as *A*_*cor*_. Pedigree errors involved in most cases a wrong paternity attribution and, less frequently, individuals supposed to be different genetically. The total number of parents after correction did not change, with an added father and a removed mother. The main change concerned the number of families that went from 39 to 35. Both *A* and *A*_*cor*_ were calculated with the R package nadiv (Wolak, 2012), and kept for the comparison in order to show the potential loss due to pedigree errors and the maximum performance attainable by pedigree. Concerning the genomic relationship, we used a normalized matrix (G Equation 3) calculated following VanRaden's formulation (Habier et al., 2007; VanRaden, 2007) and the scaling proposed by Forni et al. (2011) to assure compatibility with A, for each genotyping set (7K, 50K, 100K, and 250K) :

(3) $$\begin{array}{r} G=\frac{\left(M-P_{1}\right)W_{a}{\left(M-P_{1}\right)}^{\prime }}{trace\left[\left(M-P_{1}\right)W_{a}{\left(M-P_{1}\right)}^{\prime }\right]/n} \end{array}$$

where *M* was a genotyping matrix with *m* markers in columns and *n* individuals rows, *P*_1_ was a matrix (*n* × *p*) containing the minor allele frequency (2*p*_*i*_), at the marker *i*, and *W*_*a*_ was a matrix of weights described below. *Ad hoc* scripts in R were used to make the computations for G (R3.3.1 platform). To assess dominance effects, a dominance matrix based on the pedigree information was calculated with the R package nadiv (Wolak, 2012) with expected and observed pedigree information (*D* and *D*_*cor*_). The genomic dominance matrix was calculated as:

(4) $$\begin{array}{r} D=\frac{\left(X-P_{2}\right)W_{d}{\left(X-P_{2}\right)}^{\prime }}{trace\left[\left(X-P_{2}\right)W_{d}{\left(X-P_{2}\right)}^{\prime }\right]/n} \end{array}$$

where *X* was the genotyping (*n* × *p*) matrix containing code “0” for the homozygous and “1” for the heterozygous, *P*_2_ the (*n* × *p*) matrix containing the heterozygous frequency (2*p*_*i*_*q*_*i*_) according to Vitezica et al. (2013) and normalized in the same way as for G in Equation 3, and *W*_*d*_ the matrix of weights as described below. We used one of the procedures of Wang et al. (2012) for calculating weights in wGBLUP. Unlike GBLUP, where all markers have the same variance and therefore the same weight, the derivative wGBLUP uses a transformed G according to marker weights to select markers. The weights were calculated as $w_{j}={\hat{u}}_{j}^{2}$ where *w*_*j*_ was the weight for the SNP *j* and *û*_*j*_ was the estimated marker effect obtained as

(5) $$\begin{array}{r} {\hat{u}}_{a}=W_{a}X^{\prime }G^{-1}ĝ \end{array}$$

(6) $$\begin{array}{r} {\hat{u}}_{d}=W_{d}X^{\prime }D^{-1}\hat{d}, \end{array}$$

where *W*_*a,d*_ was a diagonal of weights, either a identity matrix (GBLUP) or a diagonal of w weights (wGBLUP) for additive (*W*_*a*_) or dominance (*W*_*d*_) relationship matrices, *ĝ* the genomic estimated breeding values (GEBV) and $\hat{d}$ the estimated dominance effects. Several iterations of recomputed *û*_*a*_, *û*_*d*_, *ĝ*, and $\hat{d}$ were performed to update G, following recommendation by Wang et al. (2012), and according to the following steps:

1. Define *i* = 1, *W*_(*a, d*)*i*_ = *I* and *G*_*i*_ as Equation (3)
2. Compute *ĝ*_*i*_ using GBLUP approach
3. Compute additive SNP effects with Equation (5) and dominance SNP effects with Equation (6)
4. Calculate SNP weights as $w_{aj+1}={\hat{u}}_{ai}^{2}$ and $w_{dj+1}={\hat{u}}_{di}^{2}$
5. Scale *w*_*aj*+1_ and *w*_*dj*+1_
6. Calculate *G*_*i*+1_ with Equation (3)
7. Calculate *D*_*i*+1_ with Equation (4)
8. *i* = *i* + 1
9. Iterate from 2 until *i* = 3.

A weighted relationship matrix was obtained from each subsequent iteration, giving respectively, Gw1, Gw2, and Gw3, as three distinct matrices leading to separate evaluation methods. In this study, therefore, eight relationship matrices were tested (A, Acor, G, Gw1, Gw2, Gw3, D, Dcor), and the resulting predictions were compared via cross-validation and by an independent data set.

### 2.6. Prediction Accuracy and Cross-Validation

We assessed the impact of the composition of the training (TS) and validation sets (VS) on the performance of the genomic evaluation by trying two TS/VS sizes and two different TS/VS compositions in a 10-fold cross-validation scheme. The two sizes were 50% (T50) and 25% (T25) of the individuals evaluated in the 2000/2001, 2012/2013, and 2014/2016 experimental trials. The last field evaluation trial of 2017/2018 did not contribute to TS and was used as an extra independent validation set (TestSet) for each of the four TS, as it represented a sample of the next generation of selection candidates. Such Testset represents an independent validation experiment without the risk of eventual overfitting that is typical of cross-validation schemes, and it is the result of a mating campaign involving a sample of parents from the breeding population (see Table 1). The two composition scenarios for TS and VS involved: a sampling of individuals independently of their family membership and a sampling of different family sets. Both size and composition were combined to obtain the desired percentage (50 or 25%) of individuals or the desired percentage (50 or 25%) of families. The performance of the models was evaluated following different criteria. Firstly, predictive ability, which was defined as the Pearson correlation coefficient between the adjusted clonal means and the GEBVs of the samples in the VS, or in the TestSet. The accuracy (Accuracy and Accuracy test) of the models were estimated by dividing each predictive ability by the square root of the heritability of the corresponding A model for the given trait. Additionally, the Spearman rank correlation between the adjusted clonal means and the GEBVs of the individuals in the VS was calculated (Spearman). We estimated the Spearman and Pearson correlation of the top 5% of the trait, for the section between 5 and 10%, and between 10 and 50% within the VS. Finally, we assessed potential bias in genomic predictions by estimating the intercept and the slope of the linear regression between the adjusted clonal means and the GEBVs of each model, in the VS and in the TestSet. Predictive abilities were also calculated at the within family level. The prediction ability obtained within families following PBLUP with the corrected pedigree were subtracted to the equivalent prediction ability obtained from the genomic model, for given cross-validation scenario and trait. A weighted average was then calculated according to the size of the family. given cross-validation scenario and trait. A weighted average was then calculated according to the size of the family.

### 2.7. Testing Factor Importance

In order to assess the main factors accounting for genomic evaluation performance, we applied the Random Forest algorithm (Liaw and Wiener, 2002) implemented in the Boruta R package (Kursa and Rudnicki, 2010). The main factors (or features) were: Trait, Matrix (A, Acor, G, Gw1, Gw2, Gw3, D, Dcor, Dw1, Dw2, Dw3), GeneticEffect (Additive, Additive, and Dominance), ST_MT (Single-Trait, Multiple-Trait), GenoSet (none, 7K,7K_homo, 50K, 250K), Type (Individual, Family), Perc (T50, T25), and PhenoSet (6 Blocs, 3 Blocs). Classification of features was done for each of the performance variables available: predicting ability, Accuracy, Spearman correlation, and slope.

## 3. Results

### 3.1. Heritabilities

**Figure 1:**
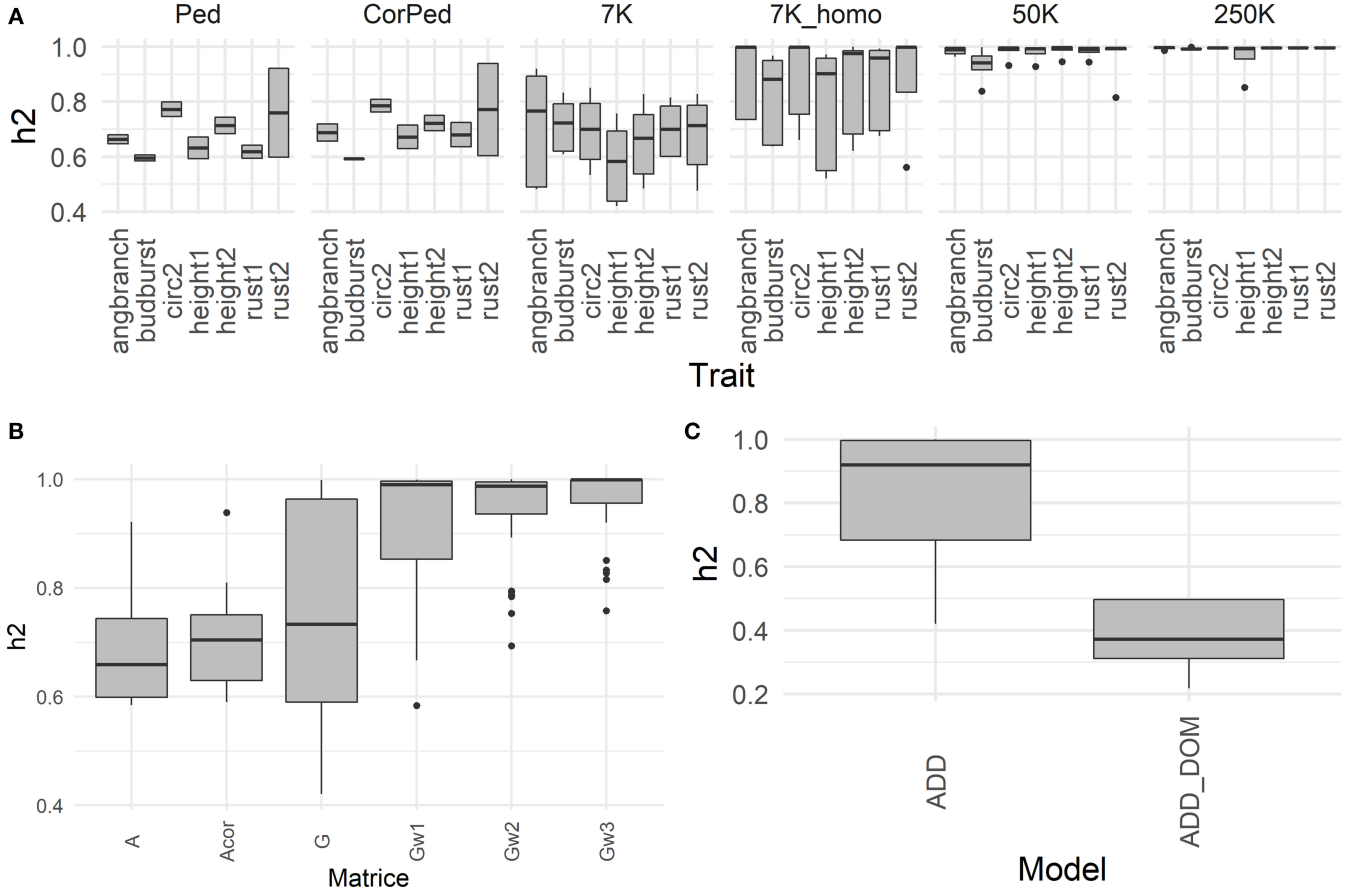
Heritability obtained with a global model using all the available data. **(A)** Heritabilities derived from an additive model and the 6-block adjusted dataset, the boxplots represent the heritabilities per trait (angbranch, budburst, circ2, height1, height2, rust1, rust2), according to the marker density (Ped, CorPed, 7K,7K_homo, 50K, and 250K), across matrix. **(B)** Heritabilities derived from an additive model and the 6-block adjusted dataset, the boxplots represent the heritabilities per matrix (A, Acor, G, Gw1, Gw2, Gw3), across traits and marker density. **(C)** Heritabilities derived from the 6-block adjusted dataset, the boxplots represent the heritabilities per model (ADD: additive; ADD_DOM), across traits, marker density, and matrix.

Heritabilities with their corresponding variance components and Akaike Information Criterion (AIC) are shown for all models and traits in the Supplementary Table 2. In general, most traits showed intermediate to high heritabilities (average of 0.73) as illustrated by Figure 1A, with height and rust showing the highest average values, and budburst correspondingly the lowest. The fact that we used adjusted clonal means as phenotypes to be explained in the models induced a low residual term, which in turn raised the heritability estimates. In terms of models, G and weighted G resulted in higher heritabilities across traits (Figure 1B), with an advantage to the latter under additive models, and to the former under models comprising also dominance (Figure 1C). Most of the genomic scenarios (G and weighted G) resulted in higher heritabilities than the pedigree-based counterparts, with uncorrected pedigree resulting in the lowest heritabilities overall. Another factor increasing heritability across traits was marker density, with highest values observed with the 250K SNPs set, followed by the 50k and the 7K_homo sets, with 7K resulting in the lowest values among genomic alternatives. On the contrary, using a phenotype adjusted with less information had little effect on heritabilities.

### 3.2. Accuracies Estimated by Cross-Validation With Different Training Sets

**Figure 2:**
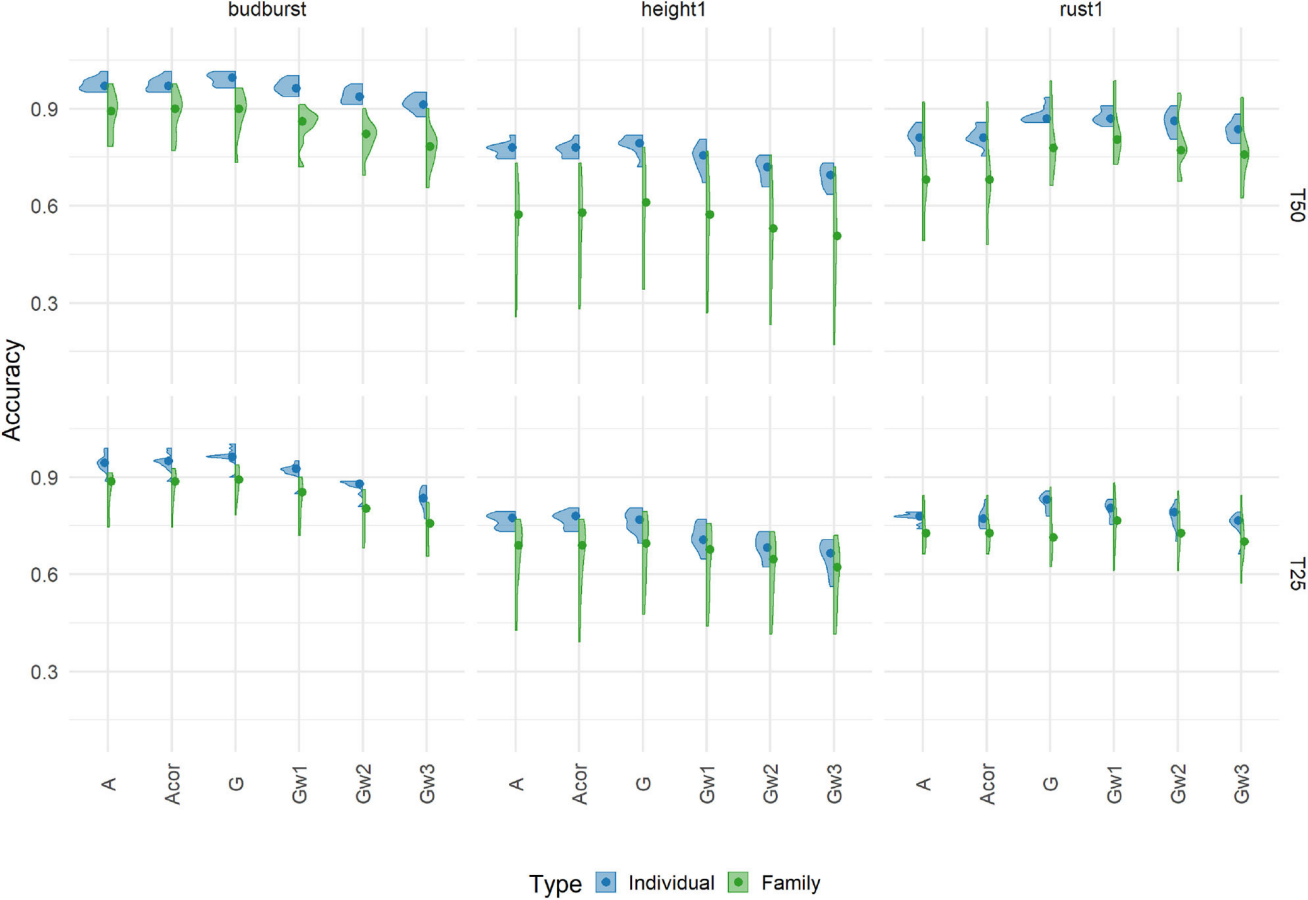
Cross-validation prediction accuracies using an additive model with 7K SNP for three traits, grouped by the proportion of individuals (Individual, in blue) or families (Family, in green), in training sets 50% (T50) and 25% (T25). Each violin plot represented the accuracy of 10 repetitions for each scenario, and the dot represented the median of each distribution.

Three out of seven traits (budburst, height1, and rust1) were selected to show the cross-validation accuracies in Figure 2 (the remaining traits are shown in Supplementary Figure 1), assuming different relationship matrices and four different training scenarios (size and composition). Results correspond to single-trait additive models with a relationship matrix based on the 7K SNP panel. Accuracies varied between 0.17 and 1.01 across all scenarios and traits. It is important to note that, because of the choice of a particular model of reference to provide a basis heritability (pedigree-based model with the A matrix), accuracies larger than one were obtained.

Accuracies responded greatly to changes in the way the training set was constituted (percentage and composition). The fact of using different families for training than for validation had a large impact on the accuracy when compared to the alternative scenario where the splitting between training and validation occurred mostly within families. Basically, as expected, predicting different families was less accurate than predicting different individuals within the same cohort, with losses in accuracy averaging 13%. This pattern was found for all traits, except for one training scenario for angbranch, where differences between the two compositions were also the weakest. Concerning the percentage, the effect of reducing the training set from T50 to T25 had also an impact on accuracy, although mostly when training and validation involved different families. On average, reduction in accuracy with decreasing training set size was around 4.2% for the training composition based on individuals, and vary depending on the trait (from −18 to 8%) for that based on families.

### 3.3. Challenging Prediction Models With New Individuals

**Figure 3:**
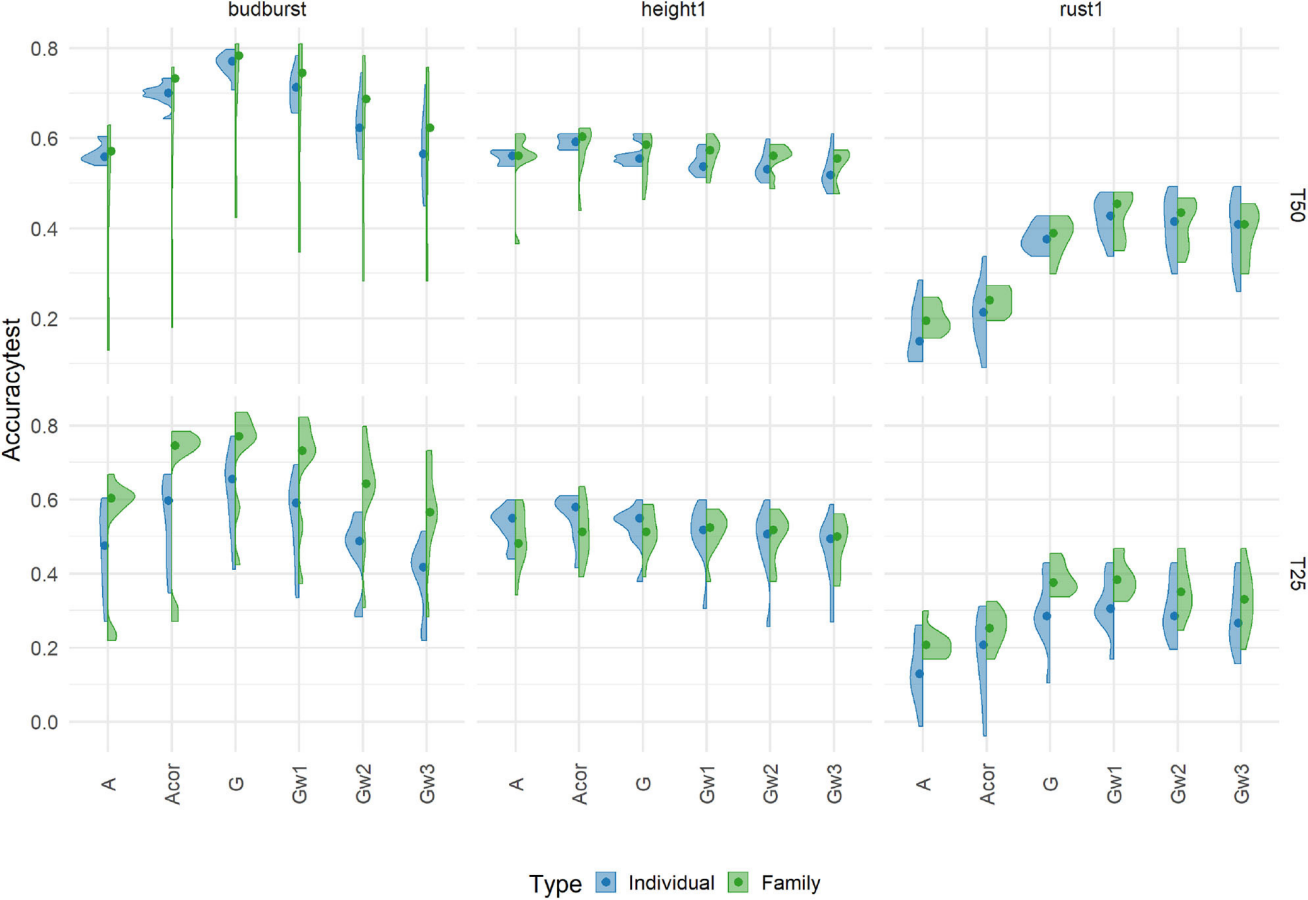
Prediction accuracies using an additive model with 7K SNP for five traits, grouped by the proportion of individuals (Individual, in blue) or families (Family, in green), in training sets 50% (T50) and 25% (T25) on an independent Test Set representing the candidates for selection. Each violin plot represented the accuracy of ten repetitions for each scenario, and the dot represented the median of each distribution.

We used a completely independent set of individuals representing the next generation of selection candidates to evaluate the different prediction models with 7K SNP and across two different training scenarios (T25 and T50). Results of accuracies from this independent set are presented for three traits in Figure 3.

Accuracies were substantially lower under the new more challenging testing scenario than those already shown for the cross-validation scheme for the same traits (see Figure 2). In general, marker-based models resulted in a less affected level of accuracy compared to the pedigree-based counterparts: the G-based and Gw1 models were the best performers, notably for rust1 and budburst. For height1, however, A and Acor models obtained comparable performances to those from genomic based models. Otherwise, the model based on uncorrected A had generally poorer accuracies than those shown by the corrected A. The behavior of the different models in terms of accuracies depended greatly on traits and, to a much lower extent, on the training scenario. Concerning the training scenario, it is to be noted that family sampling obtained slightly higher accuracies than individual sampling, although differences were not of significance. These results give an idea of the performance obtained in a real candidate selection test. In doing so, we decided to look at the impact of other factors on the independent dataset rather than on cross-validation. The results obtained for the cross-validation are in Supplementary Material.

### 3.4. Prediction Performance in the Test Set With More Complex Models

**Figure 4:**
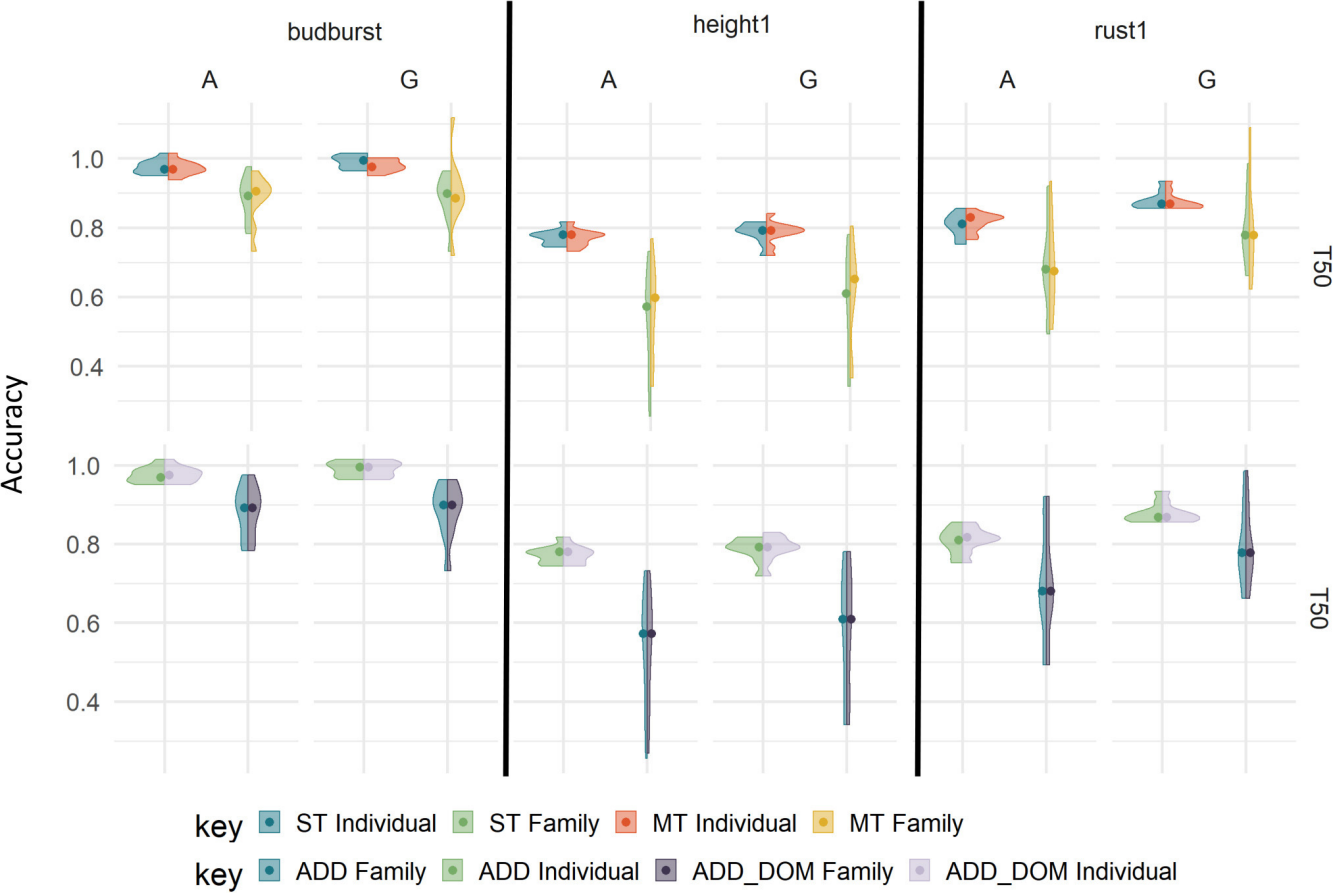
Prediction accuracies using different evaluation models on the TestSet by cross-validation type “T50” with 7K SNP, and rust1, budburst and height1. The upper panels involve single-trait (ST) vs. multiple-trait (MT) additive models: with ST with individual sampling (blue), ST with family sampling (green), MT with individual sampling (orange), and MT with family sampling (yellow). The lower panels involve additive (ADD) vs. additive and dominance (ADD_DOM) single-trait models: with ADD and individual sampling (blue), ADD and family sampling (green), ADD_DOM and individual sampling (light purple), ADD_DOM and family sampling (dark purple).

By adding a dominance effect to the single trait model for each trait with the 7K SNP panel, we did not observe significant changes in accuracy with respect to the purely additive model (Figure 4, upper part, and Supplementary Figure 2). Overall, dominance did not lead to losses in accuracy, with similar performances to that of additive counterparts across traits.

Another added complexity were the multi-trait additive models, which were also evaluated in terms of accuracies (Figure 4 lower part, and Supplementary Figure 3). The advantages of a multiple-trait approach over the single-trait counterpart were trait-dependent and generally very small. For instance, rust1 showed clearly no benefit in using a multiple-trait prediction, while for height1 the multiple-trait prediction had a small advantage when training over different families. For budburst, however, the multiple-trait approach brought a loss with the G-based model in both training scenario. Moreover, the multiple-trait approach did not seem to benefit from the use of marker-based G matrices over pedigrees. Therefore, the multiple-trait prediction did not bring a clear-cut advantage across traits and training scenarios. Genetic correlations between the traits involved in the multiple-trait analysis are shown in Supplementary Figure 4 as supplementary data.

In summary for the TestSet, the accuracy of unweighted G-based models appeared to be slightly better than with pedigree-based models, although in most cases the Acor model obtained comparable levels of performance to the best G-based method (data not shown). The cross-validation sampling strategy (individual/family) impacted the accuracy in all cases and for all traits, with individual scenarios having, in general, higher accuracy than family scenarios. The percentage of individuals in the training population (T50/T25) showed a less important impact on accuracy than that of composition. More advanced models involving dominance effects and multiple-traits did not improve the performance of genomic predictions.

### 3.5. Effect of Marker Density on Accuracies

**Figure 5:**
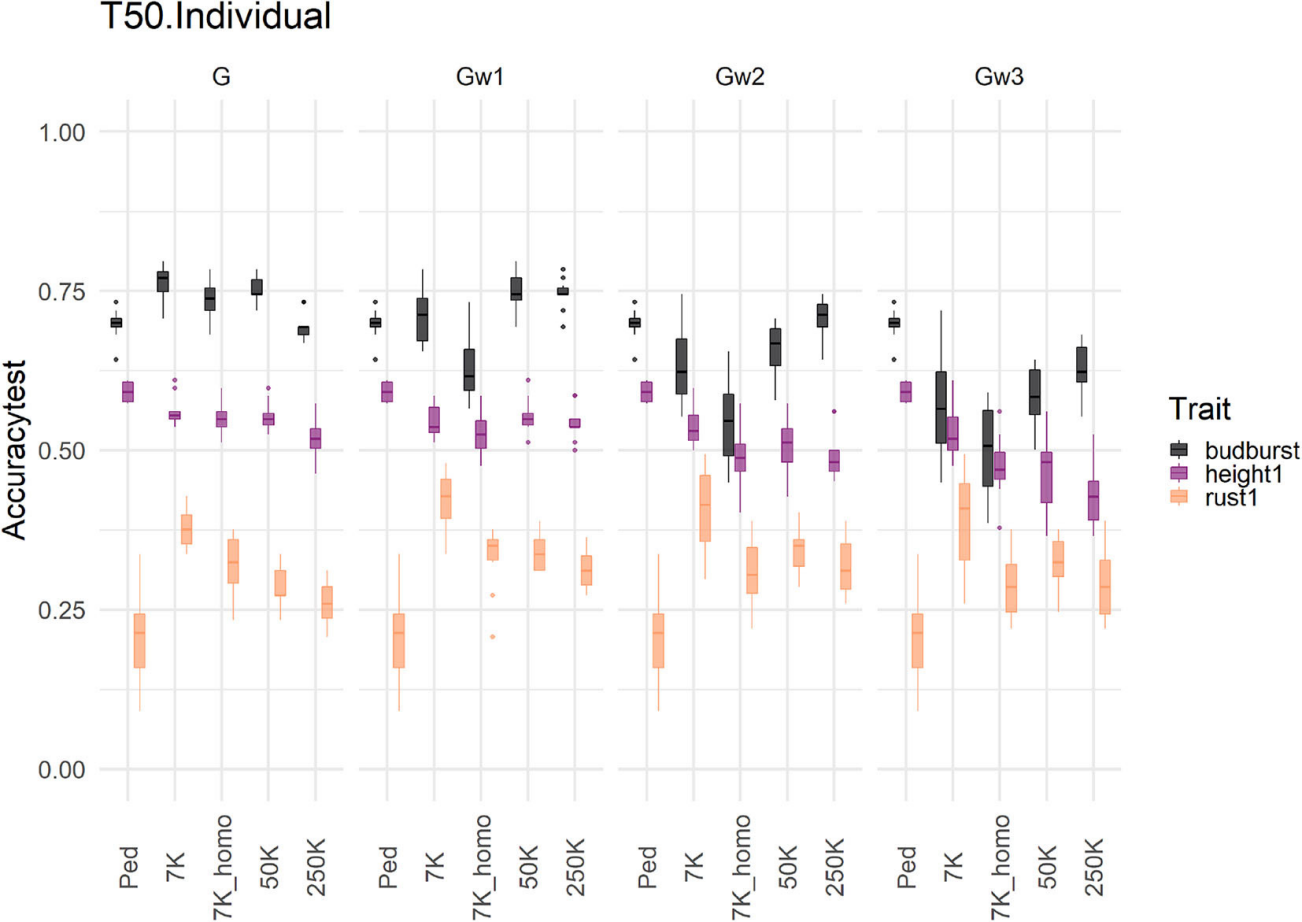
Marker densification impact on predictive accuracy of a single trait additive model with T50 individual in the TestSet for four genomic relationships matrices (in columns) and three different traits : height1 (purple), budburst (black), and rust1 (orange). The range of accuracies obtained with the pedigree information was represented in each column by the tag Ped. The accuracies distribution is represented by a boxplot.

The same three traits (budburst, height1, and rust1) were used to show the effect of an increase in marker density on prediction accuracy over different modeling approaches on the TestSet in Figure 5 (Supplementary Figures 5, 6). We compared the accuracies obtained with four marker sets of increasing density with a single-trait additive model, and T50/Individual sampling scheme.

The effects of density were clearly trait-dependent, and the choice of traits illustrated here cover well these differences in behavior. Such densities were also differently exploited according to traits by the different G matrices used in the modeling. For traits like height1 and rust1, densification in the number of SNPs had no clear benefit in terms of accuracy, and the use of weighted G matrices did not exploit the extra density to bring additional accuracy. For traits like budburst, however, densification brought some benefits in accuracy when combined with some weighting in the G matrices, notably after one step of weighting and using the highest densities of 250K.

Besides the number of markers, their distribution over the genome seemed also of relevance for accuracy. This is particularly illustrated in the comparison between the 7K and 7K_homo sets, where the latter represents an even distribution sample over the genome. Such even distribution was not beneficial for accuracies across traits compared to the original 7K set. This latter set was seemingly richer for some relevant genes, as the array design from which the 7K set results favored certain regions linked to important traits over a homogeneous distribution.

### 3.6. Challenging Prediction Models With Degraded Phenotypes

**Figure 6:**
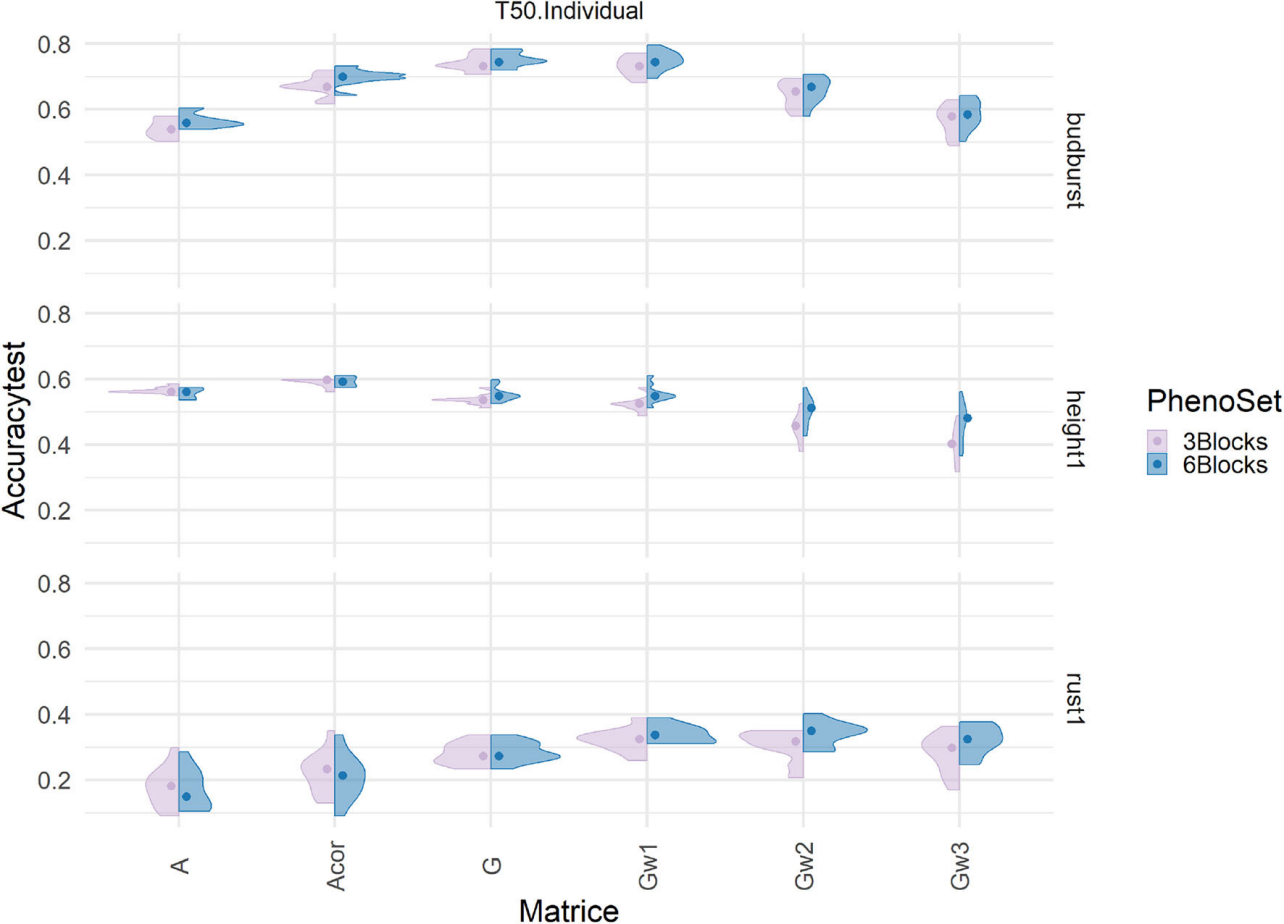
Impact on predictive accuracy of two alternative ways of producing phenotypes, with 3 (pink) and with 6 (blue) replicates, with a single trait additive model in the Test set (T50 individual sampling strategy) by genomic relationships matrices (in columns) and three different traits (in rows): height1, budburst, and rust1. The accuracies distribution is represented by a violin plot and their median by the dot.

Phenotypes used as dependent variables in the models resulted from averaging six field replicates that were previously spatially adjusted. To test whether the number of replicates could have an effect on the difference in performance between pedigree and genomic-based evaluations, new evaluations were produced based only on 3 out of 6 replicates. The adjusted clonal means produced were compared to those obtained with 6 Blocks. The correlation between the two clonal mean sets was close but not equal to 1 (from 0.8 to 0.94: Supplementary Figure 7), and a *t*-test on paired data confirmed the difference to be of significance between the two sets of data. Resulting accuracies under this new evaluation scheme are presented in Figure 6 (Supplementary Figures 8, 9 for the results in cross validation), involving the training scenario T50/individuals and the marker density set of 50K. The prediction accuracy was not significantly affected by the reduction in repetitions, across models and traits. This result was also observed for other training scenarios and for the remaining marker densities (not shown). Therefore, downgrading the phenotype with half the number of repetitions did not appear to affect pedigree-based predictions, which were almost equally competitive. This also suggests that evaluations under current conditions could have been simplified with either less field area or extended to extra candidates keeping the same field area.

### 3.7. Evaluation of Prediction Models With Complementary Criteria

Trends for slope of the linear regression between the adjusted clonal means used as phenotypes and the resulting GEBVs (or pedigree equivalents EBVs) across models showed that the pedigree-based approaches had the most robust behavior with values always around 1. Contrarily, G-based approaches often showed upwardly biased predictions (Supplementary Figures 10, 11). This deviation was always more pronounced for G-BLUP than for weighted G-BLUP, with a decreasing trend in slope with increasing steps of weighting. Marker densities had the effect of increasing slopes, notably for G-BLUP and weighted G-BLUP schemes with fewer steps of weighting. With a less pronounced effect, the change in training scenarios from individuals to families and from T50 to T25 increased slopes. In general, G-BLUP schemes showed the largest deviation in slopes due to changes in training scenarios. Slopes larger than one correspond generally to biases in predictions that depend on the magnitude of the predicted variable, being larger the bias the larger the phenotype.

**Figure 7:**
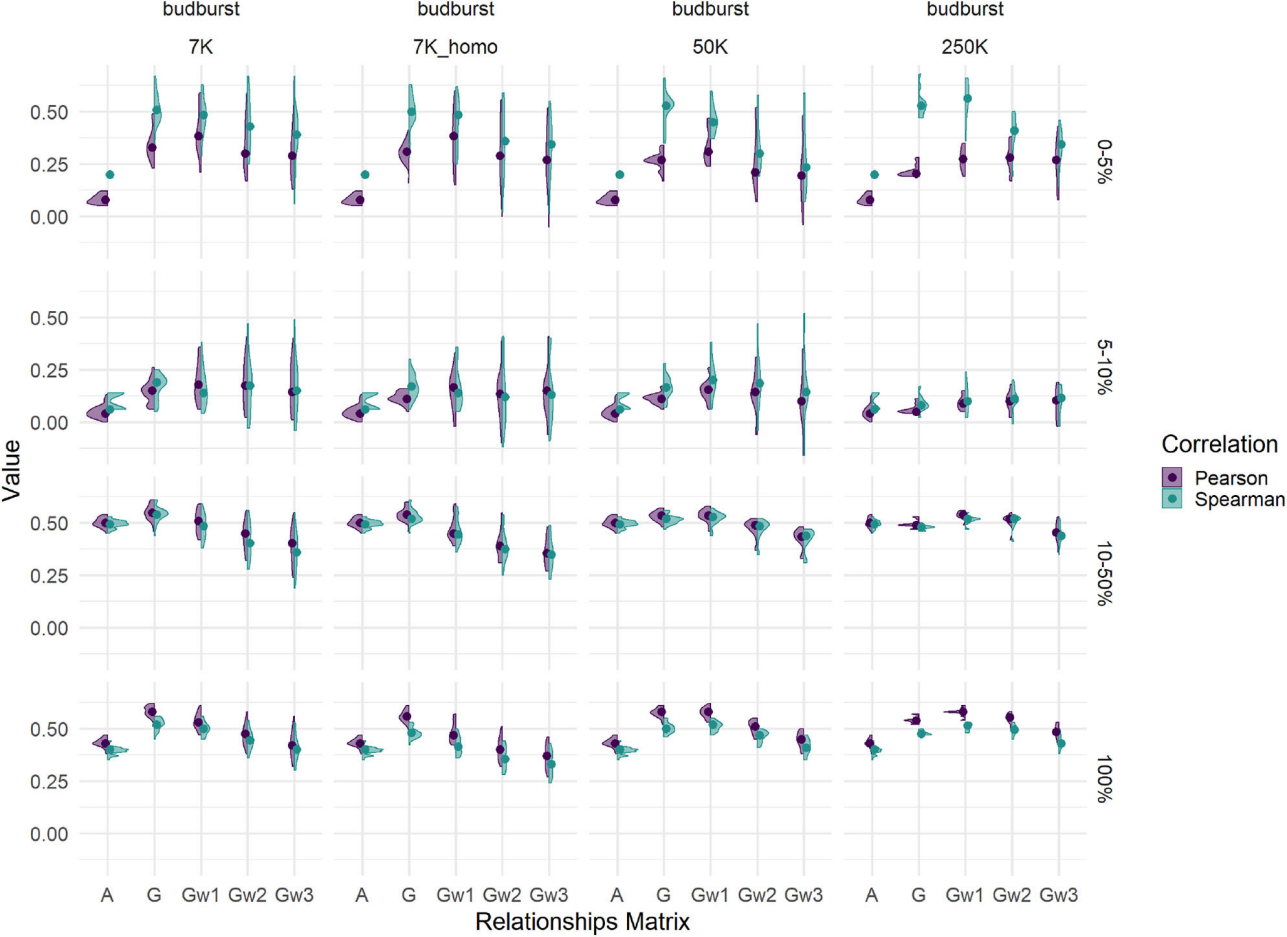
Comparison of Spearman (green) and Pearson (purple) correlations between phenotypes and estimated breeding values for budburst in the Test set (T50 individual sampling strategy), for different relationship matrices (within panels abscissas) and SNP densities (across panel columns). Across panel rows represent the tier used for the calculation of correlations: 0–5% for the 5% best individuals; 5–10%, between the 5 and 10% best individuals; 10–50%, between the 10 and 50% best individuals, and 100% for the whole Test set.

We compared two correlation coefficients: the classical Pearson correlation, on which predicting abilities are based, and a rank-based coefficient like Spearman. Such comparison was made across different tiers of the evaluated sample of candidates: from the 5% tier of best candidates to the totality of the TestSet, with the aim to explain the origin of biases. Results are shown in Figure 7 for budburst (Supplementary Figure 12 for the cross-validation results). Differences between the two coefficients were substantial within the best 5% tier, where the Spearman correlation appeared to magnify the advantages of G-based models over that of pedigree-based counterparts. Such advantage became more pronounced for that particular elite tier with G-based models using higher marker densities. Differences were less pronounced for other less performing tiers, notably those closer to the mean. For the totality of the TestSet, Pearson resulted in slightly higher values than those of Spearman. Thus, the behavior of the two correlations were opposite whether we looked at the best tier or to the whole distribution, with Spearman revealing extra differences between evaluation methods for the tail of the distribution that is usually relevant for selection. Similar patterns were observed for rust1 (Supplementary Figure 13 lower part). Height1 had a pattern slightly different, with an advantage of Spearman over Pearson for the G-based models relevant for the 50K SNP densities and for the 2 top tiers, and no advantage with the highest density 250K (Supplementary Figure 13 upper part).

### 3.8. Genomic Model to Select Among Full-Sibs

**Figure 8:**
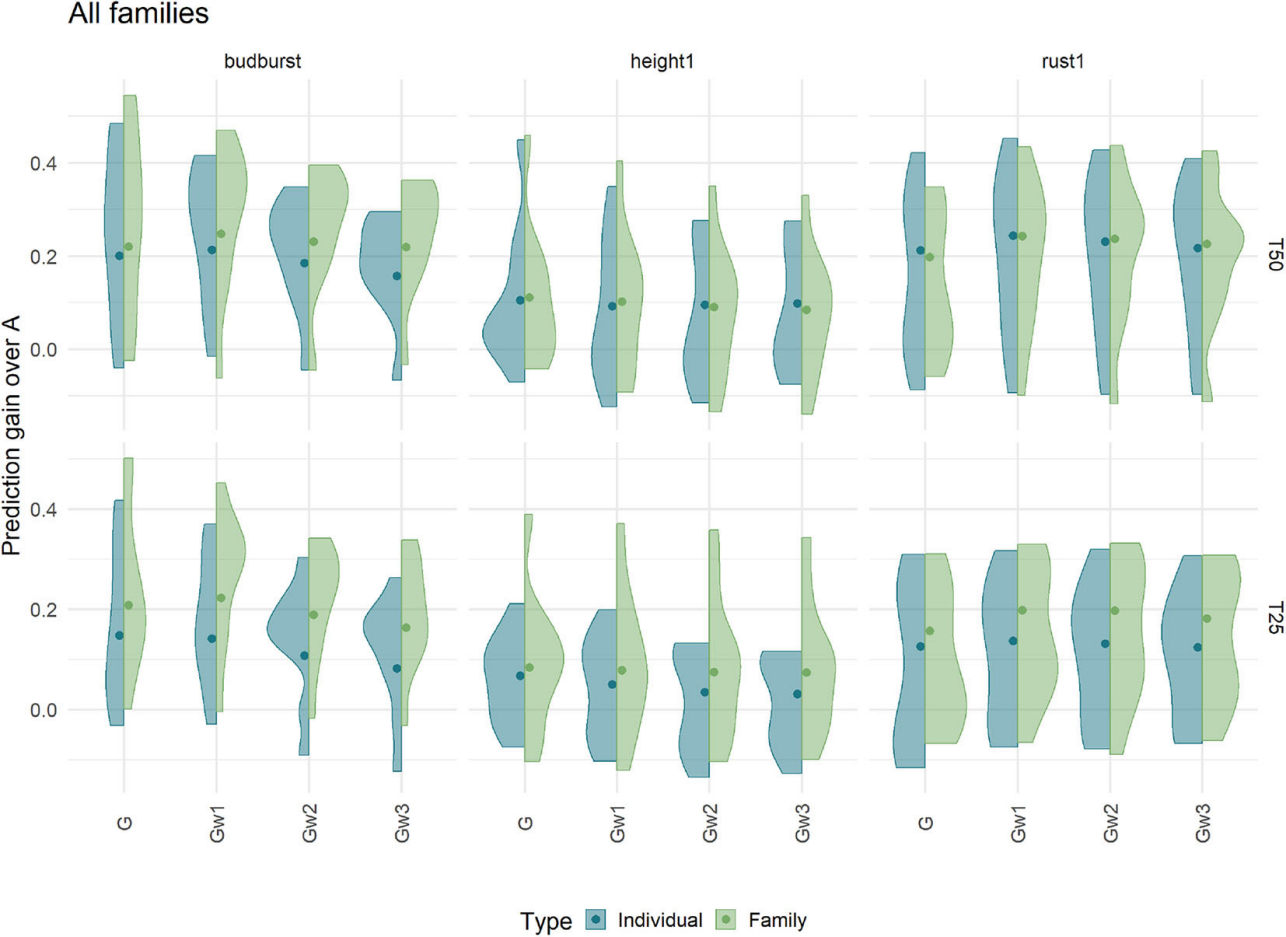
Prediction gain compared to Pedigree based predicting ability within independent test families. Predicting abilities were obtained using an additive model with 7K SNP for three traits grouped by the proportion of individuals (Individual) or families (Family) in training sets 50% (T50) and 25% (T25). The color of violin plots correspond to the sampling strategy: in blue, the individual sampling strategy and in green the family sampling strategy. Each violin plot represented the accuracy of ten repetitions for each relationship matrix. The dot represented the weighted mean of the prediction gain, the mean was weighted by the number of offspring in each family.

Differences in Prediction ability at within-family level between genome-based and pedigree-based predictions are shown in Figure 8, in the shape of distributions over all available full-sib families and for three traits. Results show important variation across families, spanning from no advantage of genome-based methods with respect to the pedigree counterpart (zero differences and below), to advantages over 0.4 for the genome-based option for some of the families. The different methods of constructing the G matrix (G and weighted G) had little effect on the differences, while increasing the training set (T50 vs. T25) or sampling families instead of individuals augmented slightly the genome-based advantage in terms of median differences. These advantages were higher for budburst and rust1 than for height1. Overall, genome-based methods showed advantages over pedigree counterparts when ranking candidates at the within-family levels, for most of the families.

### 3.9. Ranking of Factors Impacting Prediction Accuracies

**Figure 9:**
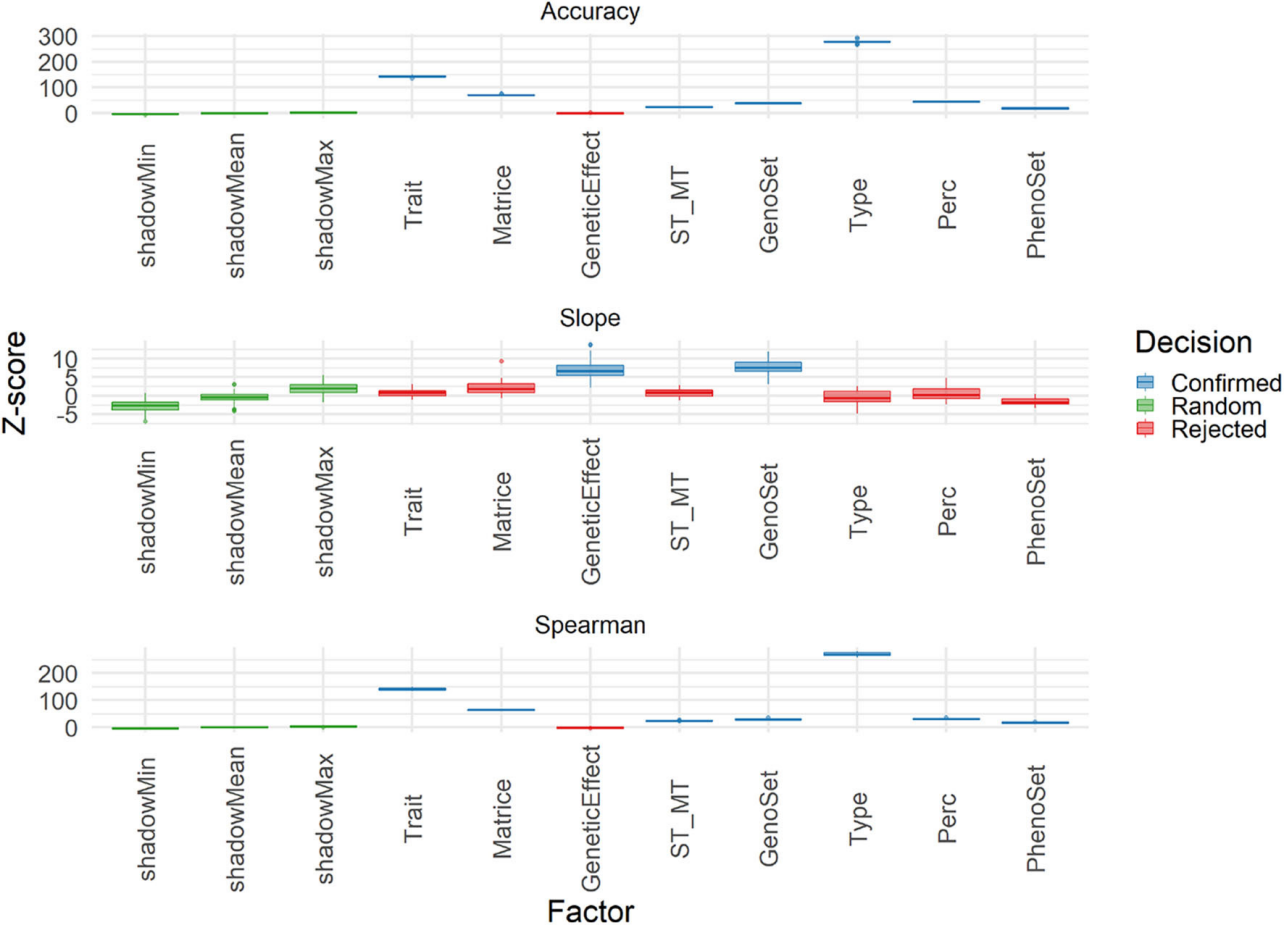
Importance (Z-score) for each feature estimated with Boruta algorithm to explain Accuracy, slope, and Spearman correlation (Spearman) variability in the validation population. Boruta shadow features were ShadowMin, ShadowMean, and ShadowMax, as random references. The test factors were Trait (rust1, rust2, height1, height2, circ2, budburst, angbranch), Matrix (A, Acor, G, Gw1, Gw2, Gw3, D), GeneticEffect (Additive, Additive, and Dominance), ST_MT (Single-Trait, Multiple-Trait), GenoSet (none, 7K,7K_homo, 50K, 250K), Type (Individual, Family), and Perc (T50, T25). Algorithm decision for each factor, based on the significativity of the difference between factors and the shadow features are: shadow features (green), confirmed (blue), and rejected (red).

**Figure 10:**
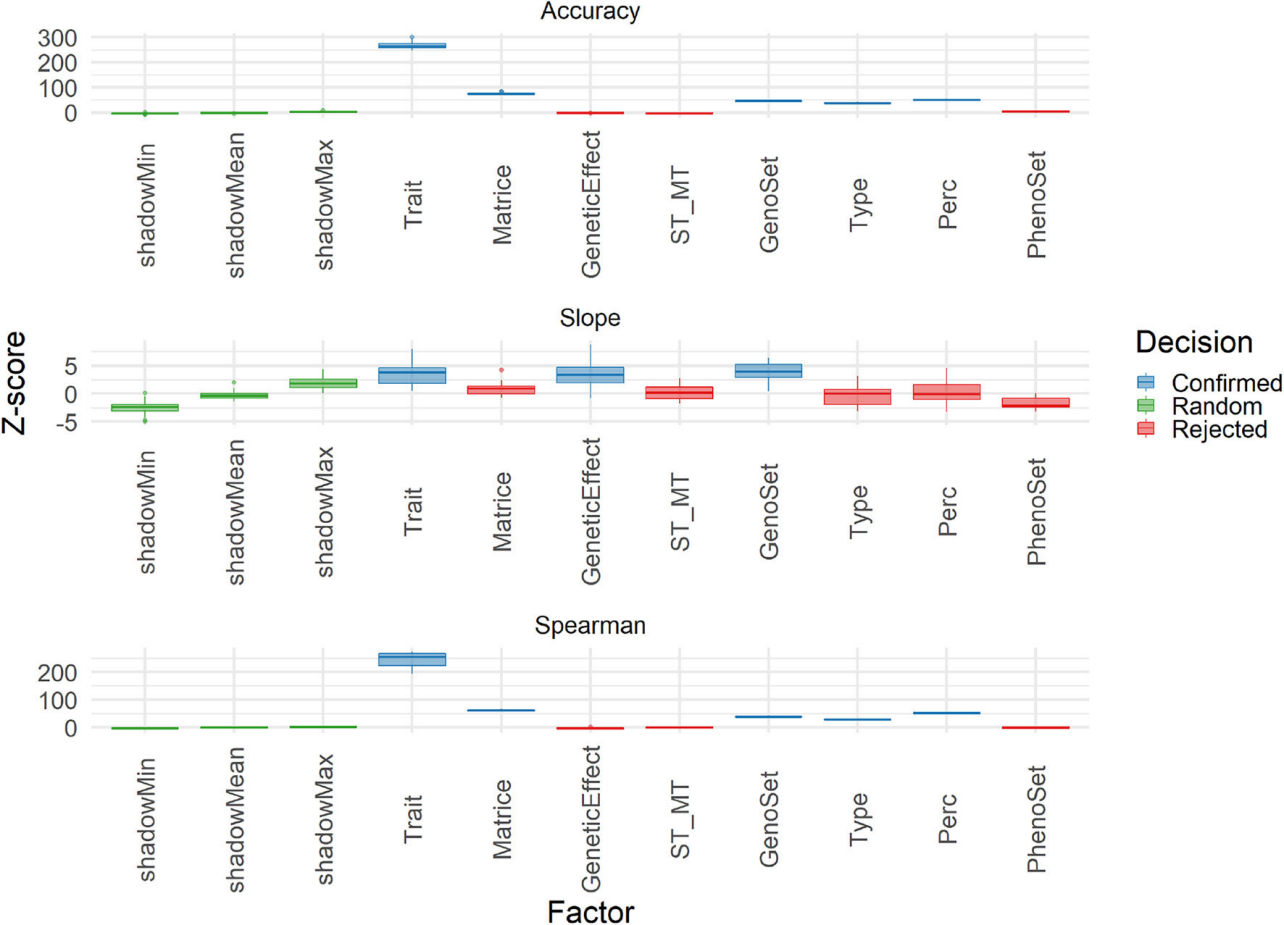
Importance (Z-score) for each features estimated with Boruta algorithm to explain Accuracy, slope, and Spearman correlation (Spearman) variability in the TestSet population. Boruta shadow features were ShadowMin, ShadowMean, and ShadowMax. The test factors were Trait (rust1, rust2, height1, height2, circ2, budburst, angbranch), Matrix (A, Acor, G, Gw1, Gw2, Gw3, D), GeneticEffect (Additive, Additive and Dominance), ST_MT (Single-Trait, Multiple-Trait), GenoSet (none, 7K,7K_homo, 50K, 250K), Type (Individual, Family), and Perc (T50, T25). Algorithm decision for each factor, based on the significativity of the difference between factors and the shadow features are: in color: Green: shadow features (green), confirmed (blue), and rejected (red).

The Boruta algorithm was used to evaluate the different features explaining the variability of three performance parameters: accuracy, Spearman correlation and slope. Results in terms of Z-score for all features in the cross-validation are shown in Figure 9. Both correlation-based performance parameters, accuracy and Spearman, led to similar ranking of features, with Type (Individual vs. Family), trait, matrix (A and G matrices), and Perc (T50 vs. T25) being the factors explaining the most in performances. Thus, the A vs. G comparison, although important, was not the one at the top. For slope, however, the features related to modeling and integrating information were the most important ones, with those related to training and validation characteristics being negligible. A similar analysis was conducted on the results obtained with the TestSet (Figure 10). Results show patterns for accuracy and Spearman correlation similar to those of cross-validation, except for the fact that the impact of size and composition of validation was negligible in TestSet conditions. For slope, the effects of the different features were very small, again with features related to modeling and integrating information showing the most important roles. The main feature explaining variability of prediction within family is the trait variation (Supplementary Figure 14).

## 4. Discussion

### 4.1. Genomics Does Not Improve Substantially Prediction Accuracy Over Pedigree in Standard Conditions

This study was conceived as a proof-of-concept of the genomic evaluation in the black poplar breeding program in order to evaluate feasibility and performance in a situation close to operational conditions for the species. Several main messages could be drawn from this study. Firstly, genome-based models captured higher heritabilities and higher additive variances than their pedigree equivalents, although this did not lead to a systematic advantage in terms of prediction accuracy for the former over the latter. Although G-BLUP obtained in general the best prediction accuracies, it was very closely followed by the evaluation based on a genomically corrected pedigree. Secondly, the benefit of densification of the marker panel for the prediction quality was not obvious, with results dependent on traits and treatment of the G matrix. Finally, the most clear advantages of genome-based methods and of marker densification were found in more challenging validation situations, when observing the ranking among the best 5% elite individuals or when importance was given to selection within families.

The genomic evaluation captured generally more genetic variance than pedigree evaluation, regardless of the trait. The number of markers fitted in the model generally increased the proportion of genetic variance explained by the model, but this occurred mostly under G-BLUP. When using a weighted GBLUP variant, the proportion of genetic variance explained by the model decreased with the cycles of weighting and selection of relevant markers. Without variable selection, plain G-BLUP, increasing the number of markers favored a better coverage of all genomic regions, including those close or inside relevant QTLs. Variable selection in weighted GBLUP could have eroded relevant variation, affecting the proportion of captured variation. This type of behavior could reflect an underlying infinitesimal-like trait architecture of the traits studied rather than a few underlying QTLs with a substantial effect (Zhang et al., 2016).

Capturing more genetic variance with marker-based models did not result necessarily in a better prediction of the phenotype than using plain A models. Our prediction accuracy was already relatively high under pedigree evaluation, probably due to the fact of using a good evaluation design with enough repetitions and spatial adjustments at individual level. Markers did not help to improve this scenario or very little. Globally, when there was a difference between pedigree-based and genomic predictions, this occurred with G or Gw1 matrices. Using several weighting cycles (Gw2 and Gw3) did not show in any case better results. Comparable results with decreasing efficiency of several cycles of weighting were found in other recent studies (Teissier et al., 2018). Our results show little or no gain by increasing marker density, even when combining densification with a variable selection method, such as Gw. This lack of gain in accuracy may suggest that we have reached a plateau and that 7K markers are sufficient for this population. Some authors have already reported plateaus in performance when increasing the number of markers: in cocoa (Romero Navarro et al., 2017), wheat (Norman et al., 2018) and eucalyptus (Kainer et al., 2018). For eucalyptus, the plateau in correlation was still not reached at 500K, while for cocoa and wheat it was reached after thousands or tens of thousands of markers. Together with the fact that pedigree evaluations already obtained high levels of prediction accuracy, there is also the point that correcting pedigrees generally had a beneficial effect, making the resulting model truly competitive in some situations and with some traits compared to genome-based models. This is not new in forest assessments, given the fact that controlled crosses are cumbersome and prone to errors. In loblolly pine (Munoz et al., 2014) and in maritime pine (Bartholomé et al., 2016), pedigree errors led to decreases in predicting ability, and by completing or correcting the pedigree the predicting ability could be increased. In the maritime pine study (Bartholomé et al., 2016), the predicting ability was improved by the completion of the pedigree information in such a way that the genomic evaluation had little extra room for improvement in predicting ability. The error rate in our pedigree was 15%, involving in most cases wrong paternity attribution of complete or partial families, or individuals supposed to be different genetically.

In our study, model complexification using a dominance effect had no effect (positive or negative) on the quality of prediction. Our results are in line with previous studies. Several studies integrated dominance or epistatic effects in the GS. The results on real datasets showed either no improvement in terms of accuracy (Heidaritabar et al., 2014; Gamal El-Dien et al., 2016; Jiang et al., 2017), even if a non-additive proportion of variance was observed for the traits, or a small improvement in prediction accuracy (Aliloo et al., 2016; Moghaddar and van der Werf, 2017; Tan et al., 2018). This so far limited success may be due to the fact that the populations under study were not big enough, nor with an optimal design to reveal the benefits of adding non-additive effects in genomic prediction. Despite a few strong genetic correlations in our population, the same observation can be drawn for the multi-trait approach, which did not bring a clear advantage to the quality of the predictions. One of the possible explanations could be found in the small difference in missing values between traits in our dataset. This has already been pinpointed as a cause of lack of performance by other authors working with a multi-trait approach (Jia and Jannink, 2012; Dos Santos et al., 2016; Lyra et al., 2017; Rambolarimanana et al., 2018). Multi-trait evaluation can help the prediction by compensating missing values in different traits and poor heritabilities (Calus and Veerkamp, 2011; Jia and Jannink, 2012; Marchal et al., 2016; Schulthess et al., 2016). It could also reduce prediction bias (Kadarmideen et al., 2003). An interesting and promising approach called “Trait-assisted genomic prediction” by Ben-Sadoun et al. (2020) allows to optimize the phenotyping cost by using a multiple-trait approach.

Apart from the general trends between pedigree vs. genomic models, results of prediction accuracy were fundamentally trait-dependent and mostly driven by the kind of training scenario being applied. This is clearly shown by the results of the Boruta algorithm, which found trait and training scenarios to be key features in explaining predicting accuracies. Similarly to other authors (Norman et al., 2018), we observed that prediction accuracy resulted in higher levels when the training and validation populations were closely related, as when the split between the two occurred at within family levels. On the contrary, prediction accuracy could be greatly affected when resulting from distant, independent validation sets. In our study, the cross-validation with individual sampling performed better than with family sampling, and this somehow limited the use of genomic evaluations to predict unobserved crosses in our population with current approaches. The size of the training set used to develop prediction calibration is often cited as an important factor (Nakaya and Isobe, 2012). Curiously, the differences between our T50 and T25 schemes (50 and 25% of individuals to construct the calibration model, respectively) was not as large as one could expect and their performances overlapping to a large degree, making sometimes the differences between the two alternative training negligible. This is presumably very dependent on the properties of the populations being used for training.

### 4.2. Genomic Prediction Advantages Are Mostly Observed in Challenging Conditions

The choice of the training and validation sets is known to have a non-negligible impact on the prediction accuracy (Rincent et al., 2012). In that sense, our results showed that there was a substantial variation around each cross-validation realization, although often the ranking in performance between realizations was preserved across scenarios, notably for the individual sampling. In general, these cross-validation cases corresponded to operational situations where validation contributes with extra selection intensities, for instance, with new crosses from known parents or additional sibs across families to select from. One additional scenario of training that could be considered as especially challenging, corresponded to the validation set of newly obtained crosses from parents that were mostly underrepresented in the cross-validation sets. This could be seen as an operational demand to incorporate comparatively new material for selection. Our results showed that such challenges (represented by the validation in the test set) affected substantially the prediction accuracy across models, although G-BLUP and Gw1 were generally the most robust performers and pedigree-based evaluations the ones with the greatest loss overall. In the cross-validation scheme, the factorial design had a relatively large influence in demographic terms in the training set. Being a system that creates a well-interconnected network of families (Sørensen et al., 2005), the factorial design seemingly favored pedigree predictions to a level that made it competitive compared to genomic predictions in the cross-validation. However, the new testing set posed a challenging prediction problem to pedigree-based models, as the relatedness between training and validation was certainly weak to support quality predictions solely from a sparse A matrix. Despite that, the situation was not always a clear-cut difference between pedigree and genome-based evaluations, as shown by traits like height1.

If the extent of relatedness thanks partly to the factorial design could have facilitated the competitiveness of pedigree-based predictions, the fact of using a high quality adjusted phenotype involving 6 repetitions was another element that could have a role in diminishing the differences between pedigree and genome-based performances in prediction terms. Actually, our results showed that downgrading the quality of clonal means used as phenotypes clearly had no differential effect between pedigree and genome-based predictions, with the latter retaining prediction quality at a level without replicate reduction. This evaluation simplification has also important operational implications for field evaluation, which need to be balanced with the genomic investments.

### 4.3. Genomic Prediction Enables the Ranking of Candidates to Selection

One of the main objectives of genetic evaluation is ultimately to rank individuals according to their breeding values, in order to use subsequently final selections as reproductors for the next generation. In that sense, identifying accurately the highest breeding values is a key element in genetic progress, and the use of predicting abilities based on a parametric correlation between predictions and true breeding values is one of the most common means of quality assessment (Daetwyler et al., 2013). This latter correlation shows a linear relationship with the genetic response (Falconer, 1981). For the poplar breeding program, however, the stress is given to the selection of genotypes for clonal dissemination at the production stage directly, rather than for gametic dispersion in seed orchards. This essential difference leads to the importance of ranking in selection decisions for poplars, as for any other domesticated species with clonal selection. When assessing the potential of genomic evaluations, it is essential to take into account the way predictions will be used for. Thus, we used alternative measures of prediction quality, like the slope of the regression of “true” breeding values on estimated breeding values. This slope represents a way to assess departures due to bias in predictions, generally caused by unequal representations of lineages in the training (Patry and Ducrocq, 2011), unbalanced data (Blair and Pollak, 1984), or the use of wrong variance estimation (Sorensen and Kennedy, 1984). Bias can lead eventually to wrong selection decisions when involving differently biased candidates. Our results suggest that G-BLUP was particularly affected by biases, with large departures toward greater slopes, i.e., best phenotypes gave proportionally higher predictions than worst phenotypes. To a lesser extent, the best weighted G-BLUP (Gw1) also presented departures in slope. Comparatively, pedigree-based predictions were perfectly unbiased with slopes of one.

This result casted some doubts on the relevance of rankings derived from G-BLUP genomic predictions. We added an alternative measure of prediction quality, the Spearman correlation between predictions and true breeding values, which is a non-parametric estimate measuring the variation of the ranking. Moreover, this focus on ranking appeared as an appealing feature in the context of poplar breeding. Although less frequent in the literature than Pearson-based predicting abilities, a few authors used Spearman correlation to evaluate the prediction quality and to serve as criterion to select evaluation approaches (González-Recio et al., 2009; Mota et al., 2018). Some other authors suggest that individual ranking strategies could be more efficient (Blondel et al., 2015).

Our comparison of Spearman vs. Pearson correlations revealed that their differences in behavior were dependent on the selected tier in the distribution used for calculations, with Spearman magnifying the advantages of G-based models and high marker densities over pedigree for the best 5–10% tiers, the tail of the distribution that is usually relevant for selection. Pearson, on the other hand, attained its maximum correlation when considering the whole population. Such a difference in behavior could be of relevance when considering different levels of selection intensities, or weights given to each trait in a selection index. Usually, the interesting part of the distribution is the top percentiles, where Spearman could be a criterion of choice. However, in some cases the interest lies at intermediate values, like for budburst. The goal here is to have trees that do not budburst too early to avoid late frosts, nor too late to avoid shortening the growing season. For those central tiers, both correlations showed similar performances.

**Figure 11:**
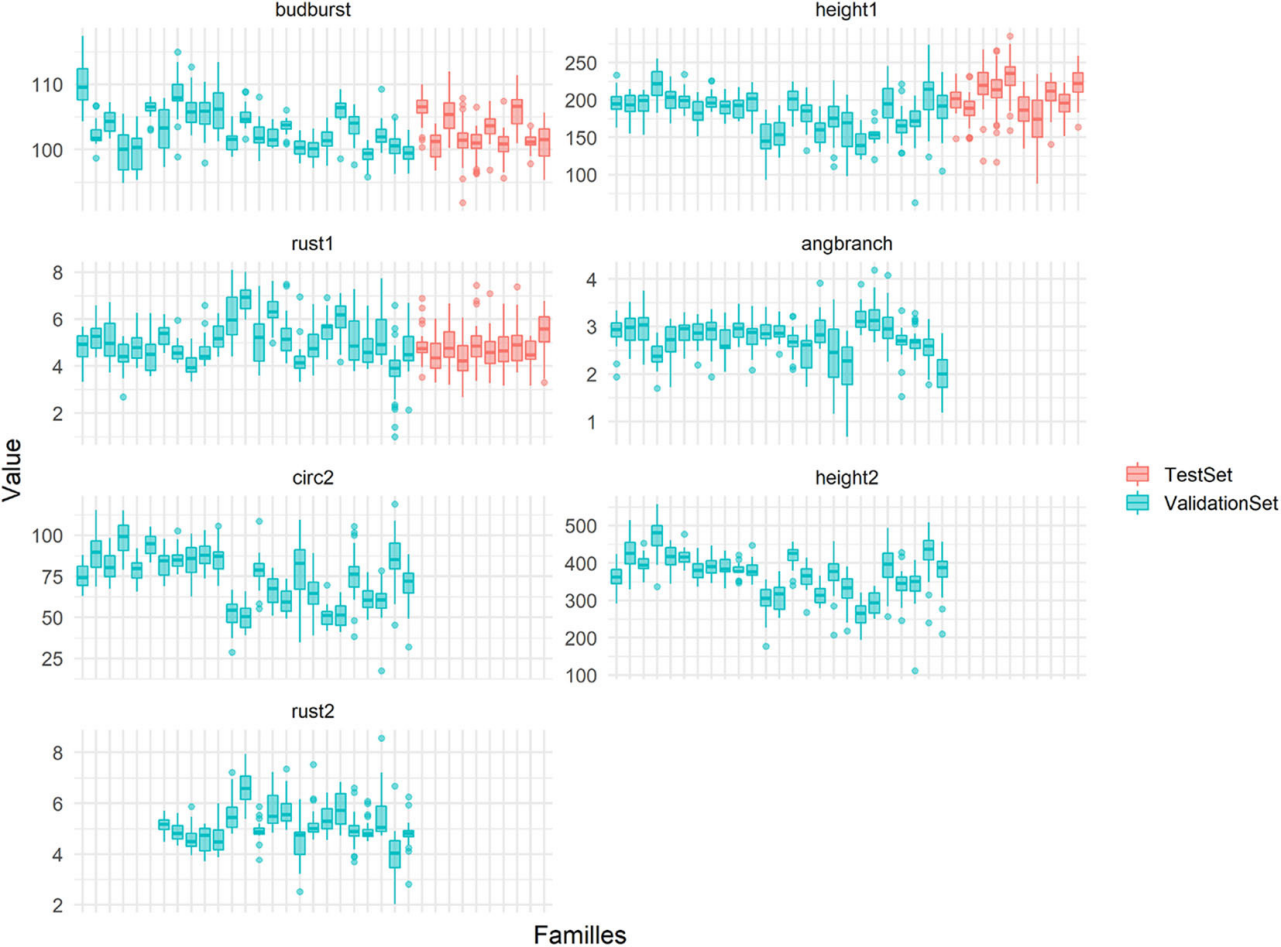
Micro-environmentally adjusted phenotypic variability by full-sibs families (in x-axis) for the seven traits used in this study. In color the families includes in the TS/VS (in blue) or in the TestSet (in pink).

We have already pinpointed the fact that the population used for training, given the level of parental factorization in their mating, presented favorable conditions for pedigree-based evaluation. One condition where genome-based evaluation is expected to outperform a pedigree counterpart is when selecting at within-family levels. Our results showed that only genome-based evaluations were able to rank sibs with some degree of accuracy within family cohorts, where pedigrees do not bring any extra information. Although such advantage over the pedigree was not clear for all the families, a majority of them showed some potential for gain over several traits. The fact that this ability did not translate into larger differences in our population could result from families of reduced size and/or from segregational variances too narrow to feed gain in a substantial way. While family sizes were not specially large for what is usual in breeding programs (on average 26 sibs per family), the variation at within-family level appeared indeed as notably reduced when compared with between-family differences (shown in Figure 11), and that for most of the traits in the analysis. This could be the result of a narrow parental variation in the training, but also from crossings between genetically similar parents, all characteristics of a reduced effective population size. Our initial estimates of effective population size (12) already pinpointed this narrow genetic diversity. A small effective size could explain to some extent the small difference that was found between our four training set scenarios, as well as the low impact of the densification in the number of markers. In that sense, it is clear that there is a need to expand this proof-of-concept approach with extra diversity.

### 4.4. Is There a Better Place in the Selection Scheme for Genomic Evaluation?

**Figure 12:**
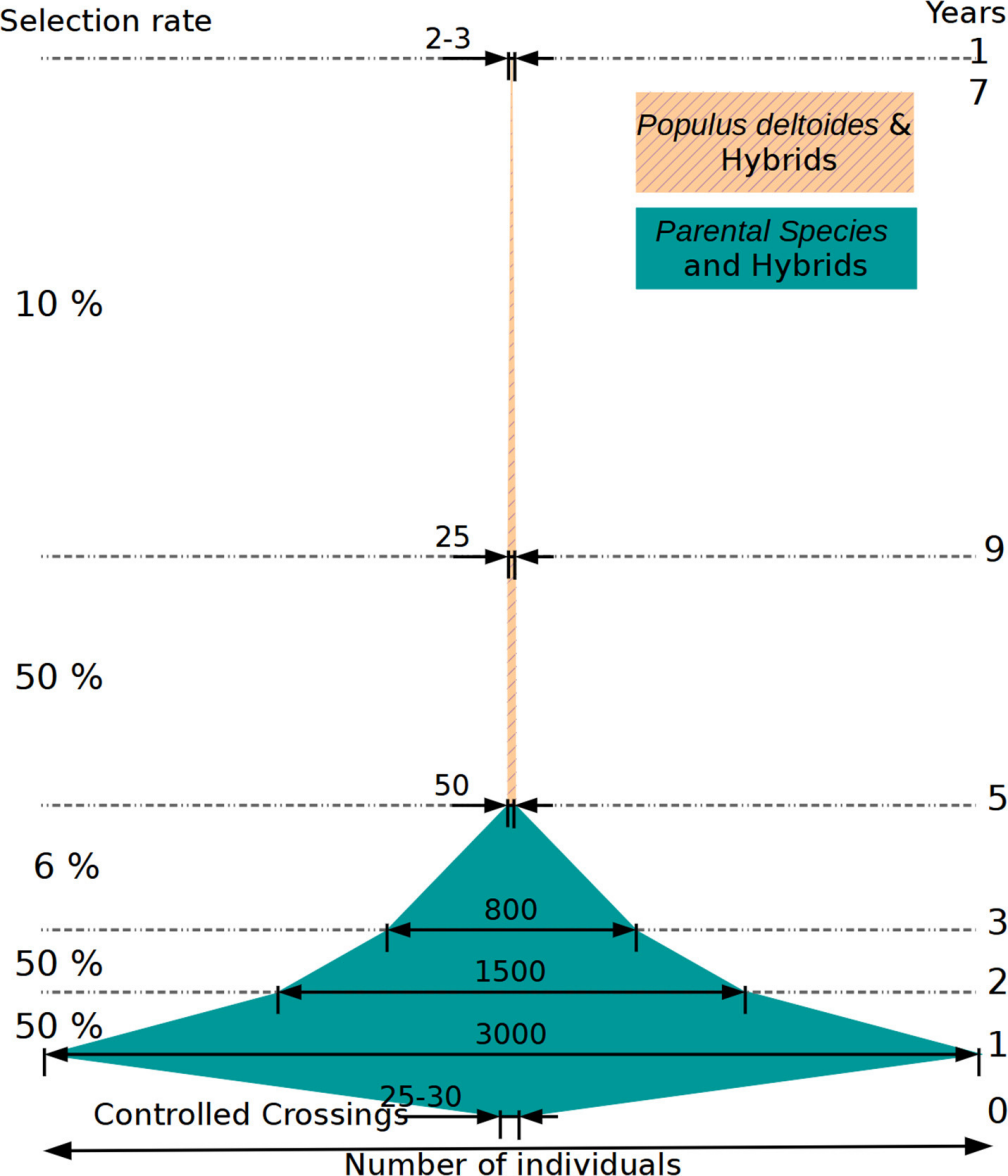
Schematic representation of a breeding cycle in poplar, with the evolution in the number of individuals and the selection rate during the different steps of selection after crossings (year 0). Numbers correspond to one cycle of selection. Selection rate values correspond to a rate relative to the previous step. The place where the genome-based evaluation test was carried out is identified by a red circle.

The present study took place at a particular step in the poplar breeding program, as illustrated in (Figure 12), specifically when evaluating selected candidates on juvenile traits in the nursery. The current selection scheme was the result of optimizing for many constraints derived from the phenotypic evaluation and operational factors over the years. It comprises several steps of selection conducted at the greenhouse, at the nursery, in the laboratory via *in vitro* tests and later in field trials, with each step implying different selection intensities and notably different selection accuracies. It is important to note that each selection step is done sequentially and conditionally onto the precedent (i.e., independent culling levels), instead of jointly and simultaneously, leading to inefficiencies with the risk of losing in the first steps important variation for subsequent steps. First steps of selection at the greenhouse and nursery are the less accurate, but the ones that screen most of the variation. Due to a limited field evaluation surface, a small number of individuals per family is kept for the next steps, reducing the phenotypic variance within each family. Conversely, later steps at the lab and in the fields are relatively accurate but screen through a subsample of original variation. Therefore, accuracy and genetic variation do not meet in a single same step for maximum efficiency in the current scheme.

Our test of genomic selection was performed with moderate to high heritability traits, well-evaluated in field trials, and on a relatively reduced set of individuals (with low effective population size) that were the result of two previous steps of selection conducted typically with a low precision and at a relatively high selection intensity (see Figure 12, with the red circle indicating where genomic evaluation was tested). These conditions are often the ones encountered in late stages in breeding program cycles, when the implementation of genomic evaluation is typically devised, and where the precious genomic and phenotypic resources that are required are to be found. This is the case, probably, of other species undergoing domestication, with elites concentrating most of the evaluation resources, and founder bases only lightly evaluated. Theoretically, there is room for improvement in the way genomic evaluation is integrated in this kind of scheme, where extra precision is specially required: at the first stages of selection. Such a scenario would involve automatically larger effective population sizes than those used here. The only drawback of such an early implementation would certainly be the costs associated with a mass genotyping, involving thousands of candidates at the greenhouse. However, with current prices attaining record low levels every year, notably with custom SNP arrays shared between species (Silva-Junior et al., 2015; Gutierrez et al., 2017), such a possibility appears now within the reach of breeding program budgets. In the case of our study, sequencing had an average cost of 400*e* per individual, although with large variations due to techniques and depths, while genotyping experienced gradual reductions during data gathering from a starting 94*e* to late 46*e* per sample (not including chip design costs).

### 4.5. Recommendations for Future Studies in Genomic Evaluation in Poplar

One of the main limitations of the study was probably the use of a training population with a design that did not correspond necessarily to what is routinely done in poplar breeding. Indeed, the factorial mating design, although potentially interesting in terms of the parental variability, was more oriented for cognitive or mapping studies. This was partially overcome by the addition of extra families and crosses, well-connected to the breeding program. In that sense, a training population truly representative of the base population for the breeding program could have made more easily generalizable the results of the study. Resampling in the existing population is a good way to improve the training population and increase the prediction accuracy. For instance, to include 6–8 trees per family and evaluation site appears to be sufficient to guarantee an accurate estimation of genetic parameters for wood density and growth in an open pollinated test of black spruce (Perron et al., 2013). For some species (Cros et al., 2015; Tayeh et al., 2015), CDmeans has given good results in optimizing the training population (Rincent et al., 2012). Some preliminary work not shown in this study, however, suggested that there is no clear advantage for such an optimal procedure, and one of the reasons could be the lack of differentiation within the population to derive truly different training sets. The optimal procedure could also be tried with a denser SNP set, like the 50K. Another strategy to optimize the training step would be to integrate existing information in the pedigree and from genetic association studies in the way proposed by Cericola et al. (2017).

Further investigations are still necessary to improve the model prediction in terms of accuracy, but also to reduce systematic and overdispersion biases. The slope bias seemed to be positively correlated with the number of markers, while the use of variable selection models like wGBLUP was able to reduce the slope bias as density was allowed to increase. Density and marker distribution of the original 7K chip did not allow GS to get a clear advantage over the pedigree-based counterpart. Marker densities lower than 7K did not appear to be of interest here, given already the slight advantage at 7K. Marker selection could be optimized to select the best repartition. Our trial of an alternative SNP set with 7K being homogeneously distributed along the genome did not lead to gains in accuracy. The original 7K array was somehow enriched for markers in some genomic regions relevant for economically important traits (Faivre-Rampant et al., 2016). Alternatively, marker repartition could follow recombination rate maps obtained from a pedigreed population, enriching in SNPs around recombination hotspots. Such distributions could be combined with haplotypic approaches based on LD information. Some studies show that haplotypic approaches could increase the reliability of predictions because of the extra capture of linkage disequilibrium with respect to single SNPs (Hess et al., 2017).

Multi-trait and multi-environment evaluations are essential in plant and tree breeding programs, although performing single-step analyses in these circumstances could be methodologically and computationally challenging. In that sense, Montesinos-Lopez et al. (2018) have proposed efficient heuristic methods based on multi-trait deep learning (MTDL), which appear to be well-adapted when data is highly unbalanced, contain missing values data and there is a need for accommodating different design factors.

GS can contribute to accelerate genetic gain by increasing the individual selection accuracy at early stages, thus shortening the generation interval, and by increasing the selection intensity. We propose to implement GS sooner in the cycle, at the seedling stage, than what was assessed in this study. In the short term, a genomic selection scheme at the seedling stage, when there is a great number of individuals taking up the least space, would be of great benefit to the breeding program. Such an early scheme combined with a multi-trait approach with a selection index can increase the genetic gain in the short term for most traits simultaneously, even for those phenotyped at maturity like wood properties. For now, only the *P. nigra* parents could be selected with such early genome-based approach, and in order to identify the best black poplar parents at the same year as the controlled-crosses to produce both pure species descendants and hybrids with other species. Time-consuming and resource-intensive evaluations could then take place only on those genomically preselected parents, with the possibility to enlarge the panel of pre-selections. In the longer term, GS can be implemented in the other parental species, *P. deltoides*, and even at the hybrid progeny (Tan et al., 2017), depending on the breeding strategy for hybrids. In this case, in addition to the step at the nursery evaluation, new steps at the laboratory can focus on other targeted traits, like interaction genotype × rust strain and woolly aphid resistance for hybrids, increasing the accuracy of prediction for costly traits related to resistance. Such propositions could save eventually from 5 up to 9 years in the breeding program. One of the evaluations for which time gains are expected is that related to wood quality, with the interesting possibility of predicting potential uses at the individual level according to the wood properties.

However, there are limits to the rapid advancements of the cycle, and we can cite here two main ones: one is regulatory and the other is of biological nature. Even if accurate genomic evaluation is available at very early stages, the release of varieties under current regulations will require carrying out evaluations under production conditions in several environments, which usually takes 10 years. Biological constraints are related to sexual maturity. Indeed, if we want to use a selected individual from a parental species for hybridization, it is necessary to wait until sexual maturity at around 7 years of age. Another added problem when dealing with sex and early selection is the sex determination, which cannot be predicted accurately from markers (Müller et al., 2020). Sex prediction at early stages could indeed save resources among the selected candidates while waiting for sexual maturity for mating.

## 5. Conclusions and Perspectives

Our proof-of-concept study shows that genomic evaluation advantages are context-dependent. Its performance could be comparable to the already well-optimized pedigree-based evaluation under certain standard conditions and with access to low to medium SNP density panels. Genomic evaluation appeared to be advantageous under less standard scenarios with a certain degree of challenge which have been pinpointed in our present work. Our study focused on a fairly advanced stage of the evaluation in the breeding program, where a substantial part of the variation has already been let aside by using pragmatic but less efficient early selections at the nursery (based on early growth, rooting ability …). We believe that genomic selection could be an interesting option at that early stage, where selection precision is typically poor and genetic variability abundant. Our study also showed that it is important to assess performances by looking at other alternative criteria, like those related to ranking, notably when these criteria respond to the operational context of the breeding program under scrutiny.

## Data Availability Statement

The datasets generated for this study can be found in online repositories. The names of the repository/repositories and accession number(s) can be found at https://data.inrae.fr/dataset.xhtml?persistentId=doi%3A10.15454%2F4FWANJ&version=DRAFT (doi: 10.15454/4FWANJ).

## Author Contributions

MP performed the analyses and drafted the manuscript. FM developed the scripts for spatial adjustment of phenotypes. VS contributed to the discussion on analytical models and data preparation, providing as well valuable scripts. CB provided the access to plant material and contributed to the view of the breeding program and ways of optimization as the scientist responsible for the *Populus nigra* breeding program. VJ and LS designed the study, discussed the analyses, assisted in drafting the manuscript and obtained funding. All authors significantly contributed to the present study, and read and approved the final manuscript.

## Conflict of Interest

The authors declare that the research was conducted in the absence of any commercial or financial relationships that could be construed as a potential conflict of interest.
