# Supplementary material for: Favorable Conditions for Genomic Evaluation to Outperform Classical Pedigree Evaluation Highlighted by a Proof-of-Concept Study in Poplar
Source: Front Plant Sci. 2020 Oct 28;11:581954. doi: 10.3389/fpls.2020.581954 (PMC7655903; doi:10.3389/fpls.2020.581954)
Supplement: Supplementary file 1 [file Data_Sheet_4.PDF]

Supplementary Material: Conditions under which genomic evaluation outperforms classical pedigree evaluation are highlighted by a proof-of-concept study in Poplar

1 SUPPLEMENTARY TABLES AND FIGURES

1.1 Tables

Table S1: Number of common individual between the four experimental trials.

	2000/2001	2012/2013	2014/2016	2017/2018
2000/2001	X	1	6	25
2012/2013	1	X	17	20
2014/2016	6	17	X	22
2017/2018	25	20	22	X

Table S2: Akaike Information Criterion (AIC) and nerrow sense heritabilities by Traits, Matrices, Models, Single or Multiple trait (ST_MT),PhenoSet, and GenoSet

Trait	Matrice	GenoSet	Model	AIC	h2	PhenoSet	Model2	Variance	Value
angbranch	G	50K	ADD	7239.6	0.9984	3Blocks	ST	varg	1915
budburst	G	50K	ADD	9007.76	0.8989	3Blocks	ST	varg	667
circ2	G	50K	ADD	8658.76	0.9984	3Blocks	ST	varg	18150
height1	G	50K	ADD	9441.46	0.9959	3Blocks	ST	varg	820.7
height2	G	50K	ADD	7430.51	0.9986	3Blocks	ST	varg	2594
rust1	G	50K	ADD	11823.45	0.9949	3Blocks	ST	varg	9045
rust2	G	50K	ADD	6819.72	0.9992	3Blocks	ST	varg	5630
angbranch	Gw1	50K	ADD	6554.8	0.997	3Blocks	ST	varg	689.3
budburst	Gw1	50K	ADD	8044.66	0.9285	3Blocks	ST	varg	297.1
circ2	Gw1	50K	ADD	7968.13	0.9966	3Blocks	ST	varg	6731
height1	Gw1	50K	ADD	8388.06	0.9906	3Blocks	ST	varg	305.3
height2	Gw1	50K	ADD	6736.35	0.997	3Blocks	ST	varg	959.8
rust1	Gw1	50K	ADD	10746.6	0.996	3Blocks	ST	varg	3337
rust2	Gw1	50K	ADD	6255.47	0.9956	3Blocks	ST	varg	2029
angbranch	Gw2	50K	ADD	6171.77	0.9953	3Blocks	ST	varg	569.3
budburst	Gw2	50K	ADD	7438.13	0.9975	3Blocks	ST	varg	254.1
circ2	Gw2	50K	ADD	7594.62	0.9884	3Blocks	ST	varg	5356
height1	Gw2	50K	ADD	7793.7	0.9918	3Blocks	ST	varg	251
height2	Gw2	50K	ADD	6371.13	0.9879	3Blocks	ST	varg	781.6
rust1	Gw2	50K	ADD	10166.72	0.9924	3Blocks	ST	varg	2902

Table S2 continued from previous page

Trait	Matrice	GenoSet	Model	AIC	h2	PhenoSet	Model2	Variance	Value
rust2	Gw2	50K	ADD	5934.26	0.9956	3Blocks	ST	varg	1523
angbranch	Gw3	50K	ADD	5894.41	0.9996	3Blocks	ST	varg	641.2
budburst	Gw3	50K	ADD	6967.14	0.9996	3Blocks	ST	varg	266.3
circ2	Gw3	50K	ADD	7309.21	0.9996	3Blocks	ST	varg	6360
height1	Gw3	50K	ADD	7343.13	0.9995	3Blocks	ST	varg	287.4
height2	Gw3	50K	ADD	6116.06	0.9995	3Blocks	ST	varg	910
rust1	Gw3	50K	ADD	9711.95	0.9995	3Blocks	ST	varg	3265
rust2	Gw3	50K	ADD	5691.51	0.9996	3Blocks	ST	varg	1718
angbranch	G	7K_homo	ADD	7232.71	0.653	3Blocks	ST	varg	1414
budburst	G	7K_homo	ADD	8919.04	0.6313	3Blocks	ST	varg	507.7
circ2	G	7K_homo	ADD	8630.32	0.6698	3Blocks	ST	varg	13420
height1	G	7K_homo	ADD	9424.86	0.4819	3Blocks	ST	varg	414.4
height2	G	7K_homo	ADD	7411.63	0.658	3Blocks	ST	varg	1897
rust1	G	7K_homo	ADD	11795.22	0.638	3Blocks	ST	varg	6594
rust2	G	7K_homo	ADD	6803.24	0.6818	3Blocks	ST	varg	4281
angbranch	Gw1	7K_homo	ADD	6751.54	0.9996	3Blocks	ST	varg	1779
budburst	Gw1	7K_homo	ADD	8366.64	0.8768	3Blocks	ST	varg	602.6
circ2	Gw1	7K_homo	ADD	8176.69	0.9996	3Blocks	ST	varg	16750
height1	Gw1	7K_homo	ADD	8960.71	0.845	3Blocks	ST	varg	711.5
height2	Gw1	7K_homo	ADD	6967.38	0.9995	3Blocks	ST	varg	2469
rust1	Gw1	7K_homo	ADD	11167.04	0.9404	3Blocks	ST	varg	8761
rust2	Gw1	7K_homo	ADD	6400.79	0.9995	3Blocks	ST	varg	4735
angbranch	Gw2	7K_homo	ADD	6267.53	0.9994	3Blocks	ST	varg	1423
budburst	Gw2	7K_homo	ADD	7961.71	0.9481	3Blocks	ST	varg	722.1
circ2	Gw2	7K_homo	ADD	7711.36	0.9995	3Blocks	ST	varg	12370
height1	Gw2	7K_homo	ADD	8528.7	0.92	3Blocks	ST	varg	761.2
height2	Gw2	7K_homo	ADD	6511.41	0.9997	3Blocks	ST	varg	1870
rust1	Gw2	7K_homo	ADD	10596.22	0.9819	3Blocks	ST	varg	8655
rust2	Gw2	7K_homo	ADD	6034.25	0.9998	3Blocks	ST	varg	3871
angbranch	Gw3	7K_homo	ADD	5969.17	0.9999	3Blocks	ST	varg	1446
budburst	Gw3	7K_homo	ADD	7668.7	0.9626	3Blocks	ST	varg	713.9
circ2	Gw3	7K_homo	ADD	7451.1	0.9998	3Blocks	ST	varg	12180
height1	Gw3	7K_homo	ADD	8273.43	0.939	3Blocks	ST	varg	766.7
height2	Gw3	7K_homo	ADD	6230.91	0.9998	3Blocks	ST	varg	1791
rust1	Gw3	7K_homo	ADD	10222.24	0.9882	3Blocks	ST	varg	8235
rust2	Gw3	7K_homo	ADD	5800.04	1	3Blocks	ST	varg	3891
angbranch	A	Ped	ADD	7251.04	0.5505	3Blocks	ST	varg	1249
budburst	A	Ped	ADD	9035.06	0.5898	3Blocks	ST	varg	551.9
circ2	A	Ped	ADD	8642.3	0.7303	3Blocks	ST	varg	17080
height1	A	Ped	ADD	9440.63	0.6096	3Blocks	ST	varg	609
height2	A	Ped	ADD	7431.25	0.7264	3Blocks	ST	varg	2485
rust1	A	Ped	ADD	11848.3	0.5409	3Blocks	ST	varg	5841
rust2	A	Ped	ADD	6812.5	0.7642	3Blocks	ST	varg	5768

Table S2 continued from previous page

Trait	Matrice	GenoSet	Model	AIC	h2	PhenoSet	Model2	Variance	Value
angbranch	Acor	Ped	ADD	7246.86	0.5457	3Blocks	ST	varg	1231
budburst	Acor	Ped	ADD	8977.2	0.5811	3Blocks	ST	varg	509.6
circ2	Acor	Ped	ADD	8641.08	0.747	3Blocks	ST	varg	17750
height1	Acor	Ped	ADD	9432.84	0.6523	3Blocks	ST	varg	668.1
height2	Acor	Ped	ADD	7427.49	0.7382	3Blocks	ST	varg	2545
rust1	Acor	Ped	ADD	11835.47	0.5724	3Blocks	ST	varg	6244
rust2	Acor	Ped	ADD	6807.11	0.7967	3Blocks	ST	varg	6157
angbranch	G	7K	ADD	7235.51	0.4541	3Blocks	ST	varg	991.4
budburst	G	7K	ADD	8890.78	0.5951	3Blocks	ST	varg	547.4
circ2	G	7K	ADD	8627.68	0.5134	3Blocks	ST	varg	10740
height1	G	7K	ADD	9409.86	0.3987	3Blocks	ST	varg	356.5
height2	G	7K	ADD	7403.67	0.5103	3Blocks	ST	varg	1530
rust1	G	7K	ADD	11779.5	0.5196	3Blocks	ST	varg	5693
rust2	G	7K	ADD	6803.73	0.4807	3Blocks	ST	varg	3062
angbranch	Gw1	7K	ADD	6959.27	0.721	3Blocks	ST	varg	1380
budburst	Gw1	7K	ADD	8528.86	0.704	3Blocks	ST	varg	512.2
circ2	Gw1	7K	ADD	8394.29	0.6748	3Blocks	ST	varg	11620
height1	Gw1	7K	ADD	9161.51	0.5525	3Blocks	ST	varg	448.1
height2	Gw1	7K	ADD	7168.52	0.6826	3Blocks	ST	varg	1700
rust1	Gw1	7K	ADD	11451.84	0.6309	3Blocks	ST	varg	5691
rust2	Gw1	7K	ADD	6595.57	0.6427	3Blocks	ST	varg	3202
angbranch	Gw2	7K	ADD	6760.27	0.8542	3Blocks	ST	varg	2174
budburst	Gw2	7K	ADD	8321.29	0.7784	3Blocks	ST	varg	602.9
circ2	Gw2	7K	ADD	8233.62	0.7686	3Blocks	ST	varg	13890
height1	Gw2	7K	ADD	8996.43	0.6533	3Blocks	ST	varg	569.1
height2	Gw2	7K	ADD	6999.76	0.78	3Blocks	ST	varg	2026
rust1	Gw2	7K	ADD	11269.7	0.7171	3Blocks	ST	varg	7053
rust2	Gw2	7K	ADD	6470.09	0.7351	3Blocks	ST	varg	3804
angbranch	Gw3	7K	ADD	6660.21	0.8912	3Blocks	ST	varg	2557
budburst	Gw3	7K	ADD	8245.59	0.8254	3Blocks	ST	varg	757.7
circ2	Gw3	7K	ADD	8151.84	0.8241	3Blocks	ST	varg	17130
height1	Gw3	7K	ADD	8922.83	0.7251	3Blocks	ST	varg	742.6
height2	Gw3	7K	ADD	6917.38	0.8403	3Blocks	ST	varg	2636
rust1	Gw3	7K	ADD	11195.62	0.7591	3Blocks	ST	varg	8283
rust2	Gw3	7K	ADD	6410.86	0.7917	3Blocks	ST	varg	4680
angbranch	G	250K	ADD	7191.25	0.9987	6Blocks	ST	varg	1632
budburst	G	250K	ADD	9104.5	0.9928	6Blocks	ST	varg	764.9
circ2	G	250K	ADD	8682.62	0.9906	6Blocks	ST	varg	17060
height1	G	250K	ADD	9314.56	0.8516	6Blocks	ST	varg	570
height2	G	250K	ADD	7434.61	0.9962	6Blocks	ST	varg	2385
rust1	G	250K	ADD	11762.95	0.9964	6Blocks	ST	varg	7828
rust2	G	250K	ADD	6820.24	0.9983	6Blocks	ST	varg	5229
angbranch	Gw1	250K	ADD	6462.08	0.9967	6Blocks	ST	varg	568.9

Table S2 continued from previous page

Trait	Matrice	GenoSet	Model	AIC	h2	PhenoSet	Model2	Variance	Value
budburst	Gw1	250K	ADD	7967.07	0.9891	6Blocks	ST	varg	269.5
circ2	Gw1	250K	ADD	7891.64	0.9969	6Blocks	ST	varg	5934
height1	Gw1	250K	ADD	8164.51	0.9896	6Blocks	ST	varg	241.5
height2	Gw1	250K	ADD	6658.75	0.9948	6Blocks	ST	varg	823.7
rust1	Gw1	250K	ADD	10625.4	0.9958	6Blocks	ST	varg	2723
rust2	Gw1	250K	ADD	6202.78	0.9948	6Blocks	ST	varg	1773
angbranch	Gw2	250K	ADD	6028.59	0.9866	6Blocks	ST	varg	397
budburst	Gw2	250K	ADD	7326.53	0.993	6Blocks	ST	varg	192.8
circ2	Gw2	250K	ADD	7456.64	0.9952	6Blocks	ST	varg	4225
height1	Gw2	250K	ADD	7469.71	0.9972	6Blocks	ST	varg	157
height2	Gw2	250K	ADD	6206.76	0.9951	6Blocks	ST	varg	537.3
rust1	Gw2	250K	ADD	9972.4	0.9972	6Blocks	ST	varg	1955
rust2	Gw2	250K	ADD	5826.7	0.9952	6Blocks	ST	varg	1130
angbranch	Gw3	250K	ADD	5721.72	0.9995	6Blocks	ST	varg	463.4
budburst	Gw3	250K	ADD	6891.37	0.9993	6Blocks	ST	varg	209.2
circ2	Gw3	250K	ADD	7158.87	0.9987	6Blocks	ST	varg	4252
height1	Gw3	250K	ADD	7054.08	0.9993	6Blocks	ST	varg	189.5
height2	Gw3	250K	ADD	5931.04	0.9995	6Blocks	ST	varg	586.9
rust1	Gw3	250K	ADD	9545.09	0.9994	6Blocks	ST	varg	2155
rust2	Gw3	250K	ADD	5601.46	0.9994	6Blocks	ST	varg	1297
angbranch	G	50K	ADD	7111.71	0.9987	6Blocks	ST	varg	1546
budburst	G	50K	ADD	8934.42	0.9156	6Blocks	ST	varg	630.6
circ2	G	50K	ADD	8518.67	0.9983	6Blocks	ST	varg	14300
height1	G	50K	ADD	9161.61	0.9974	6Blocks	ST	varg	621
height2	G	50K	ADD	7285.82	0.9989	6Blocks	ST	varg	2037
rust1	G	50K	ADD	11665.46	0.9973	6Blocks	ST	varg	7748
rust2	G	50K	ADD	6728.51	0.9957	6Blocks	ST	varg	4671
angbranch	Gw1	50K	ADD	6411.31	0.997	6Blocks	ST	varg	550
budburst	Gw1	50K	ADD	7952.5	0.9425	6Blocks	ST	varg	273.6
circ2	Gw1	50K	ADD	7803.77	0.9969	6Blocks	ST	varg	5337
height1	Gw1	50K	ADD	8091.23	0.9964	6Blocks	ST	varg	230.8
height2	Gw1	50K	ADD	6586.62	0.9971	6Blocks	ST	varg	764.6
rust1	Gw1	50K	ADD	10587.21	0.9909	6Blocks	ST	varg	2872
rust2	Gw1	50K	ADD	6153.06	0.9954	6Blocks	ST	varg	1689
angbranch	Gw2	50K	ADD	6020.07	0.9895	6Blocks	ST	varg	499.8
budburst	Gw2	50K	ADD	7385.67	0.9659	6Blocks	ST	varg	227.6
circ2	Gw2	50K	ADD	7438.16	0.9868	6Blocks	ST	varg	4406
height1	Gw2	50K	ADD	7486.42	0.9938	6Blocks	ST	varg	190.1
height2	Gw2	50K	ADD	6218.73	0.9883	6Blocks	ST	varg	608.5
rust1	Gw2	50K	ADD	10010.6	0.98	6Blocks	ST	varg	2573
rust2	Gw2	50K	ADD	5831.55	0.989	6Blocks	ST	varg	1321
angbranch	Gw3	50K	ADD	5796.11	0.9743	6Blocks	ST	varg	551.5
budburst	Gw3	50K	ADD	6948.46	0.9993	6Blocks	ST	varg	271.7

Table S2 continued from previous page

Trait	Matrice	GenoSet	Model	AIC	h2	PhenoSet	Model2	Variance	Value
circ2	Gw3	50K	ADD	7160.87	0.9996	6Blocks	ST	varg	5036
height1	Gw3	50K	ADD	7109.33	0.975	6Blocks	ST	varg	212
height2	Gw3	50K	ADD	5961.46	0.9995	6Blocks	ST	varg	709.1
rust1	Gw3	50K	ADD	9550.5	0.9995	6Blocks	ST	varg	3056
rust2	Gw3	50K	ADD	5577.39	0.9997	6Blocks	ST	varg	1573
angbranch	G	7K_homo	ADD	7092.31	0.735	6Blocks	ST	varg	1325
budburst	G	7K_homo	ADD	8842.08	0.6414	6Blocks	ST	varg	478.6
circ2	G	7K_homo	ADD	8472.27	0.754	6Blocks	ST	varg	12220
height1	G	7K_homo	ADD	9148.32	0.5498	6Blocks	ST	varg	372.2
height2	G	7K_homo	ADD	7260.14	0.6818	6Blocks	ST	varg	1548
rust1	G	7K_homo	ADD	11617.91	0.6942	6Blocks	ST	varg	6245
rust2	G	7K_homo	ADD	6690.41	0.8349	6Blocks	ST	varg	4685
angbranch	Gw1	7K_homo	ADD	6527.16	0.9996	6Blocks	ST	varg	1218
budburst	Gw1	7K_homo	ADD	8282	0.882	6Blocks	ST	varg	561
circ2	Gw1	7K_homo	ADD	7973.03	0.9995	6Blocks	ST	varg	11920
height1	Gw1	7K_homo	ADD	8604.69	0.9019	6Blocks	ST	varg	584.2
height2	Gw1	7K_homo	ADD	6827.22	0.976	6Blocks	ST	varg	1792
rust1	Gw1	7K_homo	ADD	10922.78	0.96	6Blocks	ST	varg	7437
rust2	Gw1	7K_homo	ADD	6201.85	0.9992	6Blocks	ST	varg	3172
angbranch	Gw2	7K_homo	ADD	6075.62	0.9997	6Blocks	ST	varg	1063
budburst	Gw2	7K_homo	ADD	7897.71	0.9506	6Blocks	ST	varg	692.2
circ2	Gw2	7K_homo	ADD	7553.23	0.9997	6Blocks	ST	varg	9867
height1	Gw2	7K_homo	ADD	8086.45	0.9584	6Blocks	ST	varg	599.4
height2	Gw2	7K_homo	ADD	6407.07	0.9997	6Blocks	ST	varg	1574
rust1	Gw2	7K_homo	ADD	10364.86	0.9862	6Blocks	ST	varg	7149
rust2	Gw2	7K_homo	ADD	5895.27	0.9994	6Blocks	ST	varg	3001
angbranch	Gw3	7K_homo	ADD	5785.27	0.9999	6Blocks	ST	varg	1045
budburst	Gw3	7K_homo	ADD	7592.22	0.9677	6Blocks	ST	varg	690.2
circ2	Gw3	7K_homo	ADD	7280.74	0.9999	6Blocks	ST	varg	9666
height1	Gw3	7K_homo	ADD	7747.73	0.9716	6Blocks	ST	varg	587.7
height2	Gw3	7K_homo	ADD	6155.38	0.9855	6Blocks	ST	varg	1315
rust1	Gw3	7K_homo	ADD	9994.92	0.9932	6Blocks	ST	varg	6912
rust2	Gw3	7K_homo	ADD	5679.46	0.9998	6Blocks	ST	varg	3018
angbranch	A	Ped	ADD	7113.63	0.6465	6Blocks	ST	varg	1244
budburst	A	Ped	ADD	8961.26	0.606	6Blocks	ST	varg	530.4
circ2	A	Ped	ADD	8480.9	0.7986	6Blocks	ST	varg	15030
height1	A	Ped	ADD	9175.13	0.672	6Blocks	ST	varg	537.3
height2	A	Ped	ADD	7270.52	0.7433	6Blocks	ST	varg	1971
rust1	A	Ped	ADD	11698.41	0.594	6Blocks	ST	varg	5716
rust2	A	Ped	ADD	6715.81	0.9217	6Blocks	ST	varg	6659
angbranch	Acor	Ped	ADD	7109.71	0.6572	6Blocks	ST	varg	1271
budburst	Acor	Ped	ADD	8899.47	0.5946	6Blocks	ST	varg	484.8
circ2	Acor	Ped	ADD	8479.19	0.8093	6Blocks	ST	varg	15410

Table S2 continued from previous page

Trait	Matrice	GenoSet	Model	AIC	h2	PhenoSet	Model2	Variance	Value
height1	Acor	Ped	ADD	9164.54	0.7148	6Blocks	ST	varg	585.4
height2	Acor	Ped	ADD	7266.34	0.7507	6Blocks	ST	varg	1998
rust1	Acor	Ped	ADD	11679.32	0.6355	6Blocks	ST	varg	6190
rust2	Acor	Ped	ADD	6709.44	0.9388	6Blocks	ST	varg	6889
angbranch	G	7K	ADD	7101.78	0.4891	6Blocks	ST	varg	879.5
budburst	G	7K	ADD	8805.66	0.6204	6Blocks	ST	varg	538.5
circ2	G	7K	ADD	8468.77	0.5894	6Blocks	ST	varg	10180
height1	G	7K	ADD	9139.3	0.4379	6Blocks	ST	varg	308.8
height2	G	7K	ADD	7251.78	0.5371	6Blocks	ST	varg	1281
rust1	G	7K	ADD	11587.63	0.6007	6Blocks	ST	varg	5960
rust2	G	7K	ADD	6697.58	0.5711	6Blocks	ST	varg	3233
angbranch	Gw1	7K	ADD	6785.52	0.7664	6Blocks	ST	varg	1195
budburst	Gw1	7K	ADD	8425.28	0.7238	6Blocks	ST	varg	487.6
circ2	Gw1	7K	ADD	8204.91	0.7001	6Blocks	ST	varg	9099
height1	Gw1	7K	ADD	8869.61	0.5837	6Blocks	ST	varg	366.3
height2	Gw1	7K	ADD	7015.74	0.6668	6Blocks	ST	varg	1260
rust1	Gw1	7K	ADD	11187.37	0.7005	6Blocks	ST	varg	5375
rust2	Gw1	7K	ADD	6435.88	0.7139	6Blocks	ST	varg	2874
angbranch	Gw2	7K	ADD	6555.81	0.8925	6Blocks	ST	varg	1989
budburst	Gw2	7K	ADD	8215	0.7923	6Blocks	ST	varg	572.6
circ2	Gw2	7K	ADD	8032.93	0.7944	6Blocks	ST	varg	11030
height1	Gw2	7K	ADD	8679.73	0.6933	6Blocks	ST	varg	472.9
height2	Gw2	7K	ADD	6858.89	0.7531	6Blocks	ST	varg	1457
rust1	Gw2	7K	ADD	10982.71	0.7839	6Blocks	ST	varg	6809
rust2	Gw2	7K	ADD	6304.13	0.7873	6Blocks	ST	varg	3371
angbranch	Gw3	7K	ADD	6438.42	0.9202	6Blocks	ST	varg	2354
budburst	Gw3	7K	ADD	8138.07	0.8333	6Blocks	ST	varg	706.9
circ2	Gw3	7K	ADD	7949.29	0.8509	6Blocks	ST	varg	14260
height1	Gw3	7K	ADD	8593.27	0.7578	6Blocks	ST	varg	606.6
height2	Gw3	7K	ADD	6781.64	0.8273	6Blocks	ST	varg	1990
rust1	Gw3	7K	ADD	10890.69	0.8159	6Blocks	ST	varg	7769
rust2	Gw3	7K	ADD	6246.2	0.8274	6Blocks	ST	varg	3983
angbranch	G	250K	ADD_DOM	7205.1	0.4724	6Blocks	ST	varg	780.2
budburst	G	250K	ADD_DOM	9106.38	0.4967	6Blocks	ST	varg	382.6
circ2	G	250K	ADD_DOM	8683.45	0.4968	6Blocks	ST	varg	8541
height1	G	250K	ADD_DOM	9288.4	0.4987	6Blocks	ST	varg	322.9
height2	G	250K	ADD_DOM	7438	0.4968	6Blocks	ST	varg	1190
rust1	G	250K	ADD_DOM	11764.64	0.4984	6Blocks	ST	varg	3915
rust2	G	250K	ADD_DOM	6823.68	0.4983	6Blocks	ST	varg	2611
angbranch	G	50K	ADD_DOM	7114.19	0.4992	6Blocks	ST	varg	772.3
budburst	G	50K	ADD_DOM	8936.42	0.4583	6Blocks	ST	varg	315.6
circ2	G	50K	ADD_DOM	8523.19	0.4975	6Blocks	ST	varg	7129
height1	G	50K	ADD_DOM	9165.18	0.4973	6Blocks	ST	varg	309.4

Table S2 continued from previous page

Trait	Matrice	GenoSet	Model	AIC	h2	PhenoSet	Model2	Variance	Value
height2	G	50K	ADD_DOM	7290.76	0.4978	6Blocks	ST	varg	1015
rust1	G	50K	ADD_DOM	11667.79	0.4984	6Blocks	ST	varg	3871
rust2	G	50K	ADD_DOM	6734.09	0.491	6Blocks	ST	varg	2304
angbranch	G	7K_homo	ADD_DOM	7094.31	0.369	6Blocks	ST	varg	666.4
budburst	G	7K_homo	ADD_DOM	8844.08	0.3207	6Blocks	ST	varg	239.4
circ2	G	7K_homo	ADD_DOM	8474.27	0.3774	6Blocks	ST	varg	6123
height1	G	7K_homo	ADD_DOM	9150.32	0.2749	6Blocks	ST	varg	186.1
height2	G	7K_homo	ADD_DOM	7262.14	0.3407	6Blocks	ST	varg	773.6
rust1	G	7K_homo	ADD_DOM	11619.91	0.3471	6Blocks	ST	varg	3124
rust2	G	7K_homo	ADD_DOM	6692.42	0.4194	6Blocks	ST	varg	2361
angbranch	A	Ped	ADD_DOM	7115.63	0.3235	6Blocks	ST	varg	622.5
budburst	A	Ped	ADD_DOM	8963.26	0.3029	6Blocks	ST	varg	265.1
circ2	A	Ped	ADD_DOM	8482.9	0.3994	6Blocks	ST	varg	7520
height1	A	Ped	ADD_DOM	9177.13	0.3356	6Blocks	ST	varg	268.2
height2	A	Ped	ADD_DOM	7272.52	0.3713	6Blocks	ST	varg	984
rust1	A	Ped	ADD_DOM	11700.41	0.2974	6Blocks	ST	varg	2863
rust2	A	Ped	ADD_DOM	6717.85	0.4662	6Blocks	ST	varg	3403
angbranch	Acor	Ped	ADD_DOM	7111.72	0.3301	6Blocks	ST	varg	639.8
budburst	Acor	Ped	ADD_DOM	8901.47	0.2972	6Blocks	ST	varg	242.3
circ2	Acor	Ped	ADD_DOM	8481.19	0.4041	6Blocks	ST	varg	7686
height1	Acor	Ped	ADD_DOM	9166.54	0.3588	6Blocks	ST	varg	294.5
height2	Acor	Ped	ADD_DOM	7268.34	0.3747	6Blocks	ST	varg	996.4
rust1	Acor	Ped	ADD_DOM	11681.32	0.3173	6Blocks	ST	varg	3089
rust2	Acor	Ped	ADD_DOM	6711.51	0.474	6Blocks	ST	varg	3509
angbranch	G	7K	ADD_DOM	7104.62	0.2424	6Blocks	ST	varg	434.3
budburst	G	7K	ADD_DOM	8806.77	0.3123	6Blocks	ST	varg	271.6
circ2	G	7K	ADD_DOM	8470.65	0.2943	6Blocks	ST	varg	5068
height1	G	7K	ADD_DOM	9141.99	0.2176	6Blocks	ST	varg	153
height2	G	7K	ADD_DOM	7253.57	0.2685	6Blocks	ST	varg	639.1
rust1	G	7K	ADD_DOM	11589.85	0.3006	6Blocks	ST	varg	2979
rust2	G	7K	ADD_DOM	6699.45	0.2847	6Blocks	ST	varg	1605
rust1	G	50K	ADD	45920.8	0.944	6Blocks	MT	varg	7246
rust2	G	50K	ADD	45920.8	0.8158	6Blocks	MT	varg	3666
circ2	G	50K	ADD	45920.8	0.9326	6Blocks	MT	varg	13760
height1	G	50K	ADD	45920.8	0.9285	6Blocks	MT	varg	569.7
height2	G	50K	ADD	45920.8	0.9461	6Blocks	MT	varg	2045
angbranch	G	50K	ADD	45920.8	0.9633	6Blocks	MT	varg	1482
budburst	G	50K	ADD	45920.8	0.8393	6Blocks	MT	varg	567.1
rust1	G	7K_homo	ADD	45685.92	0.6758	6Blocks	MT	varg	5994
rust2	G	7K_homo	ADD	45685.92	0.5608	6Blocks	MT	varg	2724
circ2	G	7K_homo	ADD	45685.92	0.6605	6Blocks	MT	varg	10810
height1	G	7K_homo	ADD	45685.92	0.5204	6Blocks	MT	varg	346.3
height2	G	7K_homo	ADD	45685.92	0.6211	6Blocks	MT	varg	1477

Table S2 continued from previous page

Trait	Matrice	GenoSet	Model	AIC	h2	PhenoSet	Model2	Variance	Value
angbranch	G	7K_homo	ADD	45685.92	0.7337	6Blocks	MT	varg	1319
budburst	G	7K_homo	ADD	45685.92	0.6373	6Blocks	MT	varg	474.9
rust1	A	Ped	ADD	45934.44	0.6422	6Blocks	MT	varg	6372
rust2	A	Ped	ADD	45934.44	0.5984	6Blocks	MT	varg	3290
circ2	A	Ped	ADD	45934.44	0.7467	6Blocks	MT	varg	14170
height1	A	Ped	ADD	45934.44	0.5921	6Blocks	MT	varg	448.2
height2	A	Ped	ADD	45934.44	0.6831	6Blocks	MT	varg	1861
angbranch	A	Ped	ADD	45934.44	0.6797	6Blocks	MT	varg	1329
budburst	A	Ped	ADD	45934.44	0.5843	6Blocks	MT	varg	503.7
rust1	Acor	Ped	ADD	45823.36	0.7244	6Blocks	MT	varg	7514
rust2	Acor	Ped	ADD	45823.36	0.604	6Blocks	MT	varg	3312
circ2	Acor	Ped	ADD	45823.36	0.7628	6Blocks	MT	varg	14660
height1	Acor	Ped	ADD	45823.36	0.6296	6Blocks	MT	varg	485.1
height2	Acor	Ped	ADD	45823.36	0.6941	6Blocks	MT	varg	1892
angbranch	Acor	Ped	ADD	45823.36	0.7186	6Blocks	MT	varg	1449
budburst	Acor	Ped	ADD	45823.36	0.5895	6Blocks	MT	varg	478
rust1	G	7K	ADD	45626.29	0.5987	6Blocks	MT	varg	5922
rust2	G	7K	ADD	45626.29	0.4757	6Blocks	MT	varg	2524
circ2	G	7K	ADD	45626.29	0.533	6Blocks	MT	varg	9412
height1	G	7K	ADD	45626.29	0.4207	6Blocks	MT	varg	292.2
height2	G	7K	ADD	45626.29	0.4836	6Blocks	MT	varg	1211
angbranch	G	7K	ADD	45626.29	0.4816	6Blocks	MT	varg	861.3
budburst	G	7K	ADD	45626.29	0.6087	6Blocks	MT	varg	521.2
angbranch	G	50K	ADD	7239.6	0.9984	3Blocks	ST	vard	0
budburst	G	50K	ADD	9007.76	0.8989	3Blocks	ST	vard	0
circ2	G	50K	ADD	8658.76	0.9984	3Blocks	ST	vard	0
height1	G	50K	ADD	9441.46	0.9959	3Blocks	ST	vard	0
height2	G	50K	ADD	7430.51	0.9986	3Blocks	ST	vard	0
rust1	G	50K	ADD	11823.45	0.9949	3Blocks	ST	vard	0
rust2	G	50K	ADD	6819.72	0.9992	3Blocks	ST	vard	0
angbranch	Gw1	50K	ADD	6554.8	0.997	3Blocks	ST	vard	0
budburst	Gw1	50K	ADD	8044.66	0.9285	3Blocks	ST	vard	0
circ2	Gw1	50K	ADD	7968.13	0.9966	3Blocks	ST	vard	0
height1	Gw1	50K	ADD	8388.06	0.9906	3Blocks	ST	vard	0
height2	Gw1	50K	ADD	6736.35	0.997	3Blocks	ST	vard	0
rust1	Gw1	50K	ADD	10746.6	0.996	3Blocks	ST	vard	0
rust2	Gw1	50K	ADD	6255.47	0.9956	3Blocks	ST	vard	0
angbranch	Gw2	50K	ADD	6171.77	0.9953	3Blocks	ST	vard	0
budburst	Gw2	50K	ADD	7438.13	0.9975	3Blocks	ST	vard	0
circ2	Gw2	50K	ADD	7594.62	0.9884	3Blocks	ST	vard	0
height1	Gw2	50K	ADD	7793.7	0.9918	3Blocks	ST	vard	0
height2	Gw2	50K	ADD	6371.13	0.9879	3Blocks	ST	vard	0
rust1	Gw2	50K	ADD	10166.72	0.9924	3Blocks	ST	vard	0

Table S2 continued from previous page

Trait	Matrice	GenoSet	Model	AIC	h2	PhenoSet	Model2	Variance	Value
rust2	Gw2	50K	ADD	5934.26	0.9956	3Blocks	ST	vard	0
angbranch	Gw3	50K	ADD	5894.41	0.9996	3Blocks	ST	vard	0
budburst	Gw3	50K	ADD	6967.14	0.9996	3Blocks	ST	vard	0
circ2	Gw3	50K	ADD	7309.21	0.9996	3Blocks	ST	vard	0
height1	Gw3	50K	ADD	7343.13	0.9995	3Blocks	ST	vard	0
height2	Gw3	50K	ADD	6116.06	0.9995	3Blocks	ST	vard	0
rust1	Gw3	50K	ADD	9711.95	0.9995	3Blocks	ST	vard	0
rust2	Gw3	50K	ADD	5691.51	0.9996	3Blocks	ST	vard	0
angbranch	G	7K_homo	ADD	7232.71	0.653	3Blocks	ST	vard	0
budburst	G	7K_homo	ADD	8919.04	0.6313	3Blocks	ST	vard	0
circ2	G	7K_homo	ADD	8630.32	0.6698	3Blocks	ST	vard	0
height1	G	7K_homo	ADD	9424.86	0.4819	3Blocks	ST	vard	0
height2	G	7K_homo	ADD	7411.63	0.658	3Blocks	ST	vard	0
rust1	G	7K_homo	ADD	11795.22	0.638	3Blocks	ST	vard	0
rust2	G	7K_homo	ADD	6803.24	0.6818	3Blocks	ST	vard	0
angbranch	Gw1	7K_homo	ADD	6751.54	0.9996	3Blocks	ST	vard	0
budburst	Gw1	7K_homo	ADD	8366.64	0.8768	3Blocks	ST	vard	0
circ2	Gw1	7K_homo	ADD	8176.69	0.9996	3Blocks	ST	vard	0
height1	Gw1	7K_homo	ADD	8960.71	0.845	3Blocks	ST	vard	0
height2	Gw1	7K_homo	ADD	6967.38	0.9995	3Blocks	ST	vard	0
rust1	Gw1	7K_homo	ADD	11167.04	0.9404	3Blocks	ST	vard	0
rust2	Gw1	7K_homo	ADD	6400.79	0.9995	3Blocks	ST	vard	0
angbranch	Gw2	7K_homo	ADD	6267.53	0.9994	3Blocks	ST	vard	0
budburst	Gw2	7K_homo	ADD	7961.71	0.9481	3Blocks	ST	vard	0
circ2	Gw2	7K_homo	ADD	7711.36	0.9995	3Blocks	ST	vard	0
height1	Gw2	7K_homo	ADD	8528.7	0.92	3Blocks	ST	vard	0
height2	Gw2	7K_homo	ADD	6511.41	0.9997	3Blocks	ST	vard	0
rust1	Gw2	7K_homo	ADD	10596.22	0.9819	3Blocks	ST	vard	0
rust2	Gw2	7K_homo	ADD	6034.25	0.9998	3Blocks	ST	vard	0
angbranch	Gw3	7K_homo	ADD	5969.17	0.9999	3Blocks	ST	vard	0
budburst	Gw3	7K_homo	ADD	7668.7	0.9626	3Blocks	ST	vard	0
circ2	Gw3	7K_homo	ADD	7451.1	0.9998	3Blocks	ST	vard	0
height1	Gw3	7K_homo	ADD	8273.43	0.939	3Blocks	ST	vard	0
height2	Gw3	7K_homo	ADD	6230.91	0.9998	3Blocks	ST	vard	0
rust1	Gw3	7K_homo	ADD	10222.24	0.9882	3Blocks	ST	vard	0
rust2	Gw3	7K_homo	ADD	5800.04	1	3Blocks	ST	vard	0
angbranch	A	Ped	ADD	7251.04	0.5505	3Blocks	ST	vard	0
budburst	A	Ped	ADD	9035.06	0.5898	3Blocks	ST	vard	0
circ2	A	Ped	ADD	8642.3	0.7303	3Blocks	ST	vard	0
height1	A	Ped	ADD	9440.63	0.6096	3Blocks	ST	vard	0
height2	A	Ped	ADD	7431.25	0.7264	3Blocks	ST	vard	0
rust1	A	Ped	ADD	11848.3	0.5409	3Blocks	ST	vard	0
rust2	A	Ped	ADD	6812.5	0.7642	3Blocks	ST	vard	0

Table S2 continued from previous page

Trait	Matrice	GenoSet	Model	AIC	h2	PhenoSet	Model2	Variance	Value
angbranch	Acor	Ped	ADD	7246.86	0.5457	3Blocks	ST	vard	0
budburst	Acor	Ped	ADD	8977.2	0.5811	3Blocks	ST	vard	0
circ2	Acor	Ped	ADD	8641.08	0.747	3Blocks	ST	vard	0
height1	Acor	Ped	ADD	9432.84	0.6523	3Blocks	ST	vard	0
height2	Acor	Ped	ADD	7427.49	0.7382	3Blocks	ST	vard	0
rust1	Acor	Ped	ADD	11835.47	0.5724	3Blocks	ST	vard	0
rust2	Acor	Ped	ADD	6807.11	0.7967	3Blocks	ST	vard	0
angbranch	G	7K	ADD	7235.51	0.4541	3Blocks	ST	vard	0
budburst	G	7K	ADD	8890.78	0.5951	3Blocks	ST	vard	0
circ2	G	7K	ADD	8627.68	0.5134	3Blocks	ST	vard	0
height1	G	7K	ADD	9409.86	0.3987	3Blocks	ST	vard	0
height2	G	7K	ADD	7403.67	0.5103	3Blocks	ST	vard	0
rust1	G	7K	ADD	11779.5	0.5196	3Blocks	ST	vard	0
rust2	G	7K	ADD	6803.73	0.4807	3Blocks	ST	vard	0
angbranch	Gw1	7K	ADD	6959.27	0.721	3Blocks	ST	vard	0
budburst	Gw1	7K	ADD	8528.86	0.704	3Blocks	ST	vard	0
circ2	Gw1	7K	ADD	8394.29	0.6748	3Blocks	ST	vard	0
height1	Gw1	7K	ADD	9161.51	0.5525	3Blocks	ST	vard	0
height2	Gw1	7K	ADD	7168.52	0.6826	3Blocks	ST	vard	0
rust1	Gw1	7K	ADD	11451.84	0.6309	3Blocks	ST	vard	0
rust2	Gw1	7K	ADD	6595.57	0.6427	3Blocks	ST	vard	0
angbranch	Gw2	7K	ADD	6760.27	0.8542	3Blocks	ST	vard	0
budburst	Gw2	7K	ADD	8321.29	0.7784	3Blocks	ST	vard	0
circ2	Gw2	7K	ADD	8233.62	0.7686	3Blocks	ST	vard	0
height1	Gw2	7K	ADD	8996.43	0.6533	3Blocks	ST	vard	0
height2	Gw2	7K	ADD	6999.76	0.78	3Blocks	ST	vard	0
rust1	Gw2	7K	ADD	11269.7	0.7171	3Blocks	ST	vard	0
rust2	Gw2	7K	ADD	6470.09	0.7351	3Blocks	ST	vard	0
angbranch	Gw3	7K	ADD	6660.21	0.8912	3Blocks	ST	vard	0
budburst	Gw3	7K	ADD	8245.59	0.8254	3Blocks	ST	vard	0
circ2	Gw3	7K	ADD	8151.84	0.8241	3Blocks	ST	vard	0
height1	Gw3	7K	ADD	8922.83	0.7251	3Blocks	ST	vard	0
height2	Gw3	7K	ADD	6917.38	0.8403	3Blocks	ST	vard	0
rust1	Gw3	7K	ADD	11195.62	0.7591	3Blocks	ST	vard	0
rust2	Gw3	7K	ADD	6410.86	0.7917	3Blocks	ST	vard	0
angbranch	G	250K	ADD	7191.25	0.9987	6Blocks	ST	vard	0
budburst	G	250K	ADD	9104.5	0.9928	6Blocks	ST	vard	0
circ2	G	250K	ADD	8682.62	0.9906	6Blocks	ST	vard	0
height1	G	250K	ADD	9314.56	0.8516	6Blocks	ST	vard	0
height2	G	250K	ADD	7434.61	0.9962	6Blocks	ST	vard	0
rust1	G	250K	ADD	11762.95	0.9964	6Blocks	ST	vard	0
rust2	G	250K	ADD	6820.24	0.9983	6Blocks	ST	vard	0
angbranch	Gw1	250K	ADD	6462.08	0.9967	6Blocks	ST	vard	0

Table S2 continued from previous page

Trait	Matrice	GenoSet	Model	AIC	h2	PhenoSet	Model2	Variance	Value
budburst	Gw1	250K	ADD	7967.07	0.9891	6Blocks	ST	vard	0
circ2	Gw1	250K	ADD	7891.64	0.9969	6Blocks	ST	vard	0
height1	Gw1	250K	ADD	8164.51	0.9896	6Blocks	ST	vard	0
height2	Gw1	250K	ADD	6658.75	0.9948	6Blocks	ST	vard	0
rust1	Gw1	250K	ADD	10625.4	0.9958	6Blocks	ST	vard	0
rust2	Gw1	250K	ADD	6202.78	0.9948	6Blocks	ST	vard	0
angbranch	Gw2	250K	ADD	6028.59	0.9866	6Blocks	ST	vard	0
budburst	Gw2	250K	ADD	7326.53	0.993	6Blocks	ST	vard	0
circ2	Gw2	250K	ADD	7456.64	0.9952	6Blocks	ST	vard	0
height1	Gw2	250K	ADD	7469.71	0.9972	6Blocks	ST	vard	0
height2	Gw2	250K	ADD	6206.76	0.9951	6Blocks	ST	vard	0
rust1	Gw2	250K	ADD	9972.4	0.9972	6Blocks	ST	vard	0
rust2	Gw2	250K	ADD	5826.7	0.9952	6Blocks	ST	vard	0
angbranch	Gw3	250K	ADD	5721.72	0.9995	6Blocks	ST	vard	0
budburst	Gw3	250K	ADD	6891.37	0.9993	6Blocks	ST	vard	0
circ2	Gw3	250K	ADD	7158.87	0.9987	6Blocks	ST	vard	0
height1	Gw3	250K	ADD	7054.08	0.9993	6Blocks	ST	vard	0
height2	Gw3	250K	ADD	5931.04	0.9995	6Blocks	ST	vard	0
rust1	Gw3	250K	ADD	9545.09	0.9994	6Blocks	ST	vard	0
rust2	Gw3	250K	ADD	5601.46	0.9994	6Blocks	ST	vard	0
angbranch	G	50K	ADD	7111.71	0.9987	6Blocks	ST	vard	0
budburst	G	50K	ADD	8934.42	0.9156	6Blocks	ST	vard	0
circ2	G	50K	ADD	8518.67	0.9983	6Blocks	ST	vard	0
height1	G	50K	ADD	9161.61	0.9974	6Blocks	ST	vard	0
height2	G	50K	ADD	7285.82	0.9989	6Blocks	ST	vard	0
rust1	G	50K	ADD	11665.46	0.9973	6Blocks	ST	vard	0
rust2	G	50K	ADD	6728.51	0.9957	6Blocks	ST	vard	0
angbranch	Gw1	50K	ADD	6411.31	0.997	6Blocks	ST	vard	0
budburst	Gw1	50K	ADD	7952.5	0.9425	6Blocks	ST	vard	0
circ2	Gw1	50K	ADD	7803.77	0.9969	6Blocks	ST	vard	0
height1	Gw1	50K	ADD	8091.23	0.9964	6Blocks	ST	vard	0
height2	Gw1	50K	ADD	6586.62	0.9971	6Blocks	ST	vard	0
rust1	Gw1	50K	ADD	10587.21	0.9909	6Blocks	ST	vard	0
rust2	Gw1	50K	ADD	6153.06	0.9954	6Blocks	ST	vard	0
angbranch	Gw2	50K	ADD	6020.07	0.9895	6Blocks	ST	vard	0
budburst	Gw2	50K	ADD	7385.67	0.9659	6Blocks	ST	vard	0
circ2	Gw2	50K	ADD	7438.16	0.9868	6Blocks	ST	vard	0
height1	Gw2	50K	ADD	7486.42	0.9938	6Blocks	ST	vard	0
height2	Gw2	50K	ADD	6218.73	0.9883	6Blocks	ST	vard	0
rust1	Gw2	50K	ADD	10010.6	0.98	6Blocks	ST	vard	0
rust2	Gw2	50K	ADD	5831.55	0.989	6Blocks	ST	vard	0
angbranch	Gw3	50K	ADD	5796.11	0.9743	6Blocks	ST	vard	0
budburst	Gw3	50K	ADD	6948.46	0.9993	6Blocks	ST	vard	0

Table S2 continued from previous page

Trait	Matrice	GenoSet	Model	AIC	h2	PhenoSet	Model2	Variance	Value
circ2	Gw3	50K	ADD	7160.87	0.9996	6Blocks	ST	vard	0
height1	Gw3	50K	ADD	7109.33	0.975	6Blocks	ST	vard	0
height2	Gw3	50K	ADD	5961.46	0.9995	6Blocks	ST	vard	0
rust1	Gw3	50K	ADD	9550.5	0.9995	6Blocks	ST	vard	0
rust2	Gw3	50K	ADD	5577.39	0.9997	6Blocks	ST	vard	0
angbranch	G	7K_homo	ADD	7092.31	0.735	6Blocks	ST	vard	0
budburst	G	7K_homo	ADD	8842.08	0.6414	6Blocks	ST	vard	0
circ2	G	7K_homo	ADD	8472.27	0.754	6Blocks	ST	vard	0
height1	G	7K_homo	ADD	9148.32	0.5498	6Blocks	ST	vard	0
height2	G	7K_homo	ADD	7260.14	0.6818	6Blocks	ST	vard	0
rust1	G	7K_homo	ADD	11617.91	0.6942	6Blocks	ST	vard	0
rust2	G	7K_homo	ADD	6690.41	0.8349	6Blocks	ST	vard	0
angbranch	Gw1	7K_homo	ADD	6527.16	0.9996	6Blocks	ST	vard	0
budburst	Gw1	7K_homo	ADD	8282	0.882	6Blocks	ST	vard	0
circ2	Gw1	7K_homo	ADD	7973.03	0.9995	6Blocks	ST	vard	0
height1	Gw1	7K_homo	ADD	8604.69	0.9019	6Blocks	ST	vard	0
height2	Gw1	7K_homo	ADD	6827.22	0.976	6Blocks	ST	vard	0
rust1	Gw1	7K_homo	ADD	10922.78	0.96	6Blocks	ST	vard	0
rust2	Gw1	7K_homo	ADD	6201.85	0.9992	6Blocks	ST	vard	0
angbranch	Gw2	7K_homo	ADD	6075.62	0.9997	6Blocks	ST	vard	0
budburst	Gw2	7K_homo	ADD	7897.71	0.9506	6Blocks	ST	vard	0
circ2	Gw2	7K_homo	ADD	7553.23	0.9997	6Blocks	ST	vard	0
height1	Gw2	7K_homo	ADD	8086.45	0.9584	6Blocks	ST	vard	0
height2	Gw2	7K_homo	ADD	6407.07	0.9997	6Blocks	ST	vard	0
rust1	Gw2	7K_homo	ADD	10364.86	0.9862	6Blocks	ST	vard	0
rust2	Gw2	7K_homo	ADD	5895.27	0.9994	6Blocks	ST	vard	0
angbranch	Gw3	7K_homo	ADD	5785.27	0.9999	6Blocks	ST	vard	0
budburst	Gw3	7K_homo	ADD	7592.22	0.9677	6Blocks	ST	vard	0
circ2	Gw3	7K_homo	ADD	7280.74	0.9999	6Blocks	ST	vard	0
height1	Gw3	7K_homo	ADD	7747.73	0.9716	6Blocks	ST	vard	0
height2	Gw3	7K_homo	ADD	6155.38	0.9855	6Blocks	ST	vard	0
rust1	Gw3	7K_homo	ADD	9994.92	0.9932	6Blocks	ST	vard	0
rust2	Gw3	7K_homo	ADD	5679.46	0.9998	6Blocks	ST	vard	0
angbranch	A	Ped	ADD	7113.63	0.6465	6Blocks	ST	vard	0
budburst	A	Ped	ADD	8961.26	0.606	6Blocks	ST	vard	0
circ2	A	Ped	ADD	8480.9	0.7986	6Blocks	ST	vard	0
height1	A	Ped	ADD	9175.13	0.672	6Blocks	ST	vard	0
height2	A	Ped	ADD	7270.52	0.7433	6Blocks	ST	vard	0
rust1	A	Ped	ADD	11698.41	0.594	6Blocks	ST	vard	0
rust2	A	Ped	ADD	6715.81	0.9217	6Blocks	ST	vard	0
angbranch	Acor	Ped	ADD	7109.71	0.6572	6Blocks	ST	vard	0
budburst	Acor	Ped	ADD	8899.47	0.5946	6Blocks	ST	vard	0
circ2	Acor	Ped	ADD	8479.19	0.8093	6Blocks	ST	vard	0

Table S2 continued from previous page

Trait	Matrice	GenoSet	Model	AIC	h2	PhenoSet	Model2	Variance	Value
height1	Acor	Ped	ADD	9164.54	0.7148	6Blocks	ST	vard	0
height2	Acor	Ped	ADD	7266.34	0.7507	6Blocks	ST	vard	0
rust1	Acor	Ped	ADD	11679.32	0.6355	6Blocks	ST	vard	0
rust2	Acor	Ped	ADD	6709.44	0.9388	6Blocks	ST	vard	0
angbranch	G	7K	ADD	7101.78	0.4891	6Blocks	ST	vard	0
budburst	G	7K	ADD	8805.66	0.6204	6Blocks	ST	vard	0
circ2	G	7K	ADD	8468.77	0.5894	6Blocks	ST	vard	0
height1	G	7K	ADD	9139.3	0.4379	6Blocks	ST	vard	0
height2	G	7K	ADD	7251.78	0.5371	6Blocks	ST	vard	0
rust1	G	7K	ADD	11587.63	0.6007	6Blocks	ST	vard	0
rust2	G	7K	ADD	6697.58	0.5711	6Blocks	ST	vard	0
angbranch	Gw1	7K	ADD	6785.52	0.7664	6Blocks	ST	vard	0
budburst	Gw1	7K	ADD	8425.28	0.7238	6Blocks	ST	vard	0
circ2	Gw1	7K	ADD	8204.91	0.7001	6Blocks	ST	vard	0
height1	Gw1	7K	ADD	8869.61	0.5837	6Blocks	ST	vard	0
height2	Gw1	7K	ADD	7015.74	0.6668	6Blocks	ST	vard	0
rust1	Gw1	7K	ADD	11187.37	0.7005	6Blocks	ST	vard	0
rust2	Gw1	7K	ADD	6435.88	0.7139	6Blocks	ST	vard	0
angbranch	Gw2	7K	ADD	6555.81	0.8925	6Blocks	ST	vard	0
budburst	Gw2	7K	ADD	8215	0.7923	6Blocks	ST	vard	0
circ2	Gw2	7K	ADD	8032.93	0.7944	6Blocks	ST	vard	0
height1	Gw2	7K	ADD	8679.73	0.6933	6Blocks	ST	vard	0
height2	Gw2	7K	ADD	6858.89	0.7531	6Blocks	ST	vard	0
rust1	Gw2	7K	ADD	10982.71	0.7839	6Blocks	ST	vard	0
rust2	Gw2	7K	ADD	6304.13	0.7873	6Blocks	ST	vard	0
angbranch	Gw3	7K	ADD	6438.42	0.9202	6Blocks	ST	vard	0
budburst	Gw3	7K	ADD	8138.07	0.8333	6Blocks	ST	vard	0
circ2	Gw3	7K	ADD	7949.29	0.8509	6Blocks	ST	vard	0
height1	Gw3	7K	ADD	8593.27	0.7578	6Blocks	ST	vard	0
height2	Gw3	7K	ADD	6781.64	0.8273	6Blocks	ST	vard	0
rust1	Gw3	7K	ADD	10890.69	0.8159	6Blocks	ST	vard	0
rust2	Gw3	7K	ADD	6246.2	0.8274	6Blocks	ST	vard	0
angbranch	G	250K	ADD_DOM	7205.1	0.4724	6Blocks	ST	vard	780.2
budburst	G	250K	ADD_DOM	9106.38	0.4967	6Blocks	ST	vard	382.6
circ2	G	250K	ADD_DOM	8683.45	0.4968	6Blocks	ST	vard	8541
height1	G	250K	ADD_DOM	9288.4	0.4987	6Blocks	ST	vard	322.9
height2	G	250K	ADD_DOM	7438	0.4968	6Blocks	ST	vard	1190
rust1	G	250K	ADD_DOM	11764.64	0.4984	6Blocks	ST	vard	3915
rust2	G	250K	ADD_DOM	6823.68	0.4983	6Blocks	ST	vard	2611
angbranch	G	50K	ADD_DOM	7114.19	0.4992	6Blocks	ST	vard	772.3
budburst	G	50K	ADD_DOM	8936.42	0.4583	6Blocks	ST	vard	315.6
circ2	G	50K	ADD_DOM	8523.19	0.4975	6Blocks	ST	vard	7129
height1	G	50K	ADD_DOM	9165.18	0.4973	6Blocks	ST	vard	309.4

Table S2 continued from previous page

Trait	Matrice	GenoSet	Model	AIC	h2	PhenoSet	Model2	Variance	Value
height2	G	50K	ADD_DOM	7290.76	0.4978	6Blocks	ST	vard	1015
rust1	G	50K	ADD_DOM	11667.79	0.4984	6Blocks	ST	vard	3871
rust2	G	50K	ADD_DOM	6734.09	0.491	6Blocks	ST	vard	2304
angbranch	G	7K_homo	ADD_DOM	7094.31	0.369	6Blocks	ST	vard	666.4
budburst	G	7K_homo	ADD_DOM	8844.08	0.3207	6Blocks	ST	vard	239.4
circ2	G	7K_homo	ADD_DOM	8474.27	0.3774	6Blocks	ST	vard	6123
height1	G	7K_homo	ADD_DOM	9150.32	0.2749	6Blocks	ST	vard	186.1
height2	G	7K_homo	ADD_DOM	7262.14	0.3407	6Blocks	ST	vard	773.6
rust1	G	7K_homo	ADD_DOM	11619.91	0.3471	6Blocks	ST	vard	3124
rust2	G	7K_homo	ADD_DOM	6692.42	0.4194	6Blocks	ST	vard	2361
angbranch	A	Ped	ADD_DOM	7115.63	0.3235	6Blocks	ST	vard	622.5
budburst	A	Ped	ADD_DOM	8963.26	0.3029	6Blocks	ST	vard	265.1
circ2	A	Ped	ADD_DOM	8482.9	0.3994	6Blocks	ST	vard	7520
height1	A	Ped	ADD_DOM	9177.13	0.3356	6Blocks	ST	vard	268.2
height2	A	Ped	ADD_DOM	7272.52	0.3713	6Blocks	ST	vard	984
rust1	A	Ped	ADD_DOM	11700.41	0.2974	6Blocks	ST	vard	2863
rust2	A	Ped	ADD_DOM	6717.85	0.4662	6Blocks	ST	vard	3403
angbranch	Acor	Ped	ADD_DOM	7111.72	0.3301	6Blocks	ST	vard	639.8
budburst	Acor	Ped	ADD_DOM	8901.47	0.2972	6Blocks	ST	vard	242.3
circ2	Acor	Ped	ADD_DOM	8481.19	0.4041	6Blocks	ST	vard	7686
height1	Acor	Ped	ADD_DOM	9166.54	0.3588	6Blocks	ST	vard	294.5
height2	Acor	Ped	ADD_DOM	7268.34	0.3747	6Blocks	ST	vard	996.4
rust1	Acor	Ped	ADD_DOM	11681.32	0.3173	6Blocks	ST	vard	3089
rust2	Acor	Ped	ADD_DOM	6711.51	0.474	6Blocks	ST	vard	3509
angbranch	G	7K	ADD_DOM	7104.62	0.2424	6Blocks	ST	vard	434.3
budburst	G	7K	ADD_DOM	8806.77	0.3123	6Blocks	ST	vard	271.6
circ2	G	7K	ADD_DOM	8470.65	0.2943	6Blocks	ST	vard	5068
height1	G	7K	ADD_DOM	9141.99	0.2176	6Blocks	ST	vard	153
height2	G	7K	ADD_DOM	7253.57	0.2685	6Blocks	ST	vard	639.1
rust1	G	7K	ADD_DOM	11589.85	0.3006	6Blocks	ST	vard	2979
rust2	G	7K	ADD_DOM	6699.45	0.2847	6Blocks	ST	vard	1605
rust1	G	50K	ADD	45920.8	0.944	6Blocks	MT	vard	0
rust2	G	50K	ADD	45920.8	0.8158	6Blocks	MT	vard	0
circ2	G	50K	ADD	45920.8	0.9326	6Blocks	MT	vard	0
height1	G	50K	ADD	45920.8	0.9285	6Blocks	MT	vard	0
height2	G	50K	ADD	45920.8	0.9461	6Blocks	MT	vard	0
angbranch	G	50K	ADD	45920.8	0.9633	6Blocks	MT	vard	0
budburst	G	50K	ADD	45920.8	0.8393	6Blocks	MT	vard	0
rust1	G	7K_homo	ADD	45685.92	0.6758	6Blocks	MT	vard	0
rust2	G	7K_homo	ADD	45685.92	0.5608	6Blocks	MT	vard	0
circ2	G	7K_homo	ADD	45685.92	0.6605	6Blocks	MT	vard	0
height1	G	7K_homo	ADD	45685.92	0.5204	6Blocks	MT	vard	0
height2	G	7K_homo	ADD	45685.92	0.6211	6Blocks	MT	vard	0

Table S2 continued from previous page

Trait	Matrice	GenoSet	Model	AIC	h2	PhenoSet	Model2	Variance	Value
angbranch	G	7K_homo	ADD	45685.92	0.7337	6Blocks	MT	vard	0
budburst	G	7K_homo	ADD	45685.92	0.6373	6Blocks	MT	vard	0
rust1	A	Ped	ADD	45934.44	0.6422	6Blocks	MT	vard	0
rust2	A	Ped	ADD	45934.44	0.5984	6Blocks	MT	vard	0
circ2	A	Ped	ADD	45934.44	0.7467	6Blocks	MT	vard	0
height1	A	Ped	ADD	45934.44	0.5921	6Blocks	MT	vard	0
height2	A	Ped	ADD	45934.44	0.6831	6Blocks	MT	vard	0
angbranch	A	Ped	ADD	45934.44	0.6797	6Blocks	MT	vard	0
budburst	A	Ped	ADD	45934.44	0.5843	6Blocks	MT	vard	0
rust1	Acor	Ped	ADD	45823.36	0.7244	6Blocks	MT	vard	0
rust2	Acor	Ped	ADD	45823.36	0.604	6Blocks	MT	vard	0
circ2	Acor	Ped	ADD	45823.36	0.7628	6Blocks	MT	vard	0
height1	Acor	Ped	ADD	45823.36	0.6296	6Blocks	MT	vard	0
height2	Acor	Ped	ADD	45823.36	0.6941	6Blocks	MT	vard	0
angbranch	Acor	Ped	ADD	45823.36	0.7186	6Blocks	MT	vard	0
budburst	Acor	Ped	ADD	45823.36	0.5895	6Blocks	MT	vard	0
rust1	G	7K	ADD	45626.29	0.5987	6Blocks	MT	vard	0
rust2	G	7K	ADD	45626.29	0.4757	6Blocks	MT	vard	0
circ2	G	7K	ADD	45626.29	0.533	6Blocks	MT	vard	0
height1	G	7K	ADD	45626.29	0.4207	6Blocks	MT	vard	0
height2	G	7K	ADD	45626.29	0.4836	6Blocks	MT	vard	0
angbranch	G	7K	ADD	45626.29	0.4816	6Blocks	MT	vard	0
budburst	G	7K	ADD	45626.29	0.6087	6Blocks	MT	vard	0
angbranch	G	50K	ADD	7239.6	0.9984	3Blocks	ST	vare	3.095
budburst	G	50K	ADD	9007.76	0.8989	3Blocks	ST	vare	74.98
circ2	G	50K	ADD	8658.76	0.9984	3Blocks	ST	vare	29.09
height1	G	50K	ADD	9441.46	0.9959	3Blocks	ST	vare	3.356
height2	G	50K	ADD	7430.51	0.9986	3Blocks	ST	vare	3.539
rust1	G	50K	ADD	11823.45	0.9949	3Blocks	ST	vare	46.46
rust2	G	50K	ADD	6819.72	0.9992	3Blocks	ST	vare	4.387
angbranch	Gw1	50K	ADD	6554.8	0.997	3Blocks	ST	vare	2.098
budburst	Gw1	50K	ADD	8044.66	0.9285	3Blocks	ST	vare	22.87
circ2	Gw1	50K	ADD	7968.13	0.9966	3Blocks	ST	vare	22.94
height1	Gw1	50K	ADD	8388.06	0.9906	3Blocks	ST	vare	2.887
height2	Gw1	50K	ADD	6736.35	0.997	3Blocks	ST	vare	2.902
rust1	Gw1	50K	ADD	10746.6	0.996	3Blocks	ST	vare	13.52
rust2	Gw1	50K	ADD	6255.47	0.9956	3Blocks	ST	vare	9.025
angbranch	Gw2	50K	ADD	6171.77	0.9953	3Blocks	ST	vare	2.699
budburst	Gw2	50K	ADD	7438.13	0.9975	3Blocks	ST	vare	0.6322
circ2	Gw2	50K	ADD	7594.62	0.9884	3Blocks	ST	vare	62.67
height1	Gw2	50K	ADD	7793.7	0.9918	3Blocks	ST	vare	2.087
height2	Gw2	50K	ADD	6371.13	0.9879	3Blocks	ST	vare	9.546
rust1	Gw2	50K	ADD	10166.72	0.9924	3Blocks	ST	vare	22.35

Table S2 continued from previous page

Trait	Matrice	GenoSet	Model	AIC	h2	PhenoSet	Model2	Variance	Value
rust2	Gw2	50K	ADD	5934.26	0.9956	3Blocks	ST	vare	6.762
angbranch	Gw3	50K	ADD	5894.41	0.9996	3Blocks	ST	vare	0.2292
budburst	Gw3	50K	ADD	6967.14	0.9996	3Blocks	ST	vare	0.1194
circ2	Gw3	50K	ADD	7309.21	0.9996	3Blocks	ST	vare	2.683
height1	Gw3	50K	ADD	7343.13	0.9995	3Blocks	ST	vare	0.1425
height2	Gw3	50K	ADD	6116.06	0.9995	3Blocks	ST	vare	0.4432
rust1	Gw3	50K	ADD	9711.95	0.9995	3Blocks	ST	vare	1.527
rust2	Gw3	50K	ADD	5691.51	0.9996	3Blocks	ST	vare	0.6203
angbranch	G	7K_homo	ADD	7232.71	0.653	3Blocks	ST	vare	751.4
budburst	G	7K_homo	ADD	8919.04	0.6313	3Blocks	ST	vare	296.5
circ2	G	7K_homo	ADD	8630.32	0.6698	3Blocks	ST	vare	6615
height1	G	7K_homo	ADD	9424.86	0.4819	3Blocks	ST	vare	445.6
height2	G	7K_homo	ADD	7411.63	0.658	3Blocks	ST	vare	986.1
rust1	G	7K_homo	ADD	11795.22	0.638	3Blocks	ST	vare	3741
rust2	G	7K_homo	ADD	6803.24	0.6818	3Blocks	ST	vare	1998
angbranch	Gw1	7K_homo	ADD	6751.54	0.9996	3Blocks	ST	vare	0.6939
budburst	Gw1	7K_homo	ADD	8366.64	0.8768	3Blocks	ST	vare	84.66
circ2	Gw1	7K_homo	ADD	8176.69	0.9996	3Blocks	ST	vare	7.419
height1	Gw1	7K_homo	ADD	8960.71	0.845	3Blocks	ST	vare	130.5
height2	Gw1	7K_homo	ADD	6967.38	0.9995	3Blocks	ST	vare	1.186
rust1	Gw1	7K_homo	ADD	11167.04	0.9404	3Blocks	ST	vare	555.6
rust2	Gw1	7K_homo	ADD	6400.79	0.9995	3Blocks	ST	vare	2.276
angbranch	Gw2	7K_homo	ADD	6267.53	0.9994	3Blocks	ST	vare	0.8772
budburst	Gw2	7K_homo	ADD	7961.71	0.9481	3Blocks	ST	vare	39.56
circ2	Gw2	7K_homo	ADD	7711.36	0.9995	3Blocks	ST	vare	6.475
height1	Gw2	7K_homo	ADD	8528.7	0.92	3Blocks	ST	vare	66.18
height2	Gw2	7K_homo	ADD	6511.41	0.9997	3Blocks	ST	vare	0.5283
rust1	Gw2	7K_homo	ADD	10596.22	0.9819	3Blocks	ST	vare	159.3
rust2	Gw2	7K_homo	ADD	6034.25	0.9998	3Blocks	ST	vare	0.682
angbranch	Gw3	7K_homo	ADD	5969.17	0.9999	3Blocks	ST	vare	0.1973
budburst	Gw3	7K_homo	ADD	7668.7	0.9626	3Blocks	ST	vare	27.71
circ2	Gw3	7K_homo	ADD	7451.1	0.9998	3Blocks	ST	vare	1.943
height1	Gw3	7K_homo	ADD	8273.43	0.939	3Blocks	ST	vare	49.81
height2	Gw3	7K_homo	ADD	6230.91	0.9998	3Blocks	ST	vare	0.3117
rust1	Gw3	7K_homo	ADD	10222.24	0.9882	3Blocks	ST	vare	98.03
rust2	Gw3	7K_homo	ADD	5800.04	1	3Blocks	ST	vare	0.1719
angbranch	A	Ped	ADD	7251.04	0.5505	3Blocks	ST	vare	1020
budburst	A	Ped	ADD	9035.06	0.5898	3Blocks	ST	vare	383.8
circ2	A	Ped	ADD	8642.3	0.7303	3Blocks	ST	vare	6309
height1	A	Ped	ADD	9440.63	0.6096	3Blocks	ST	vare	390
height2	A	Ped	ADD	7431.25	0.7264	3Blocks	ST	vare	935.8
rust1	A	Ped	ADD	11848.3	0.5409	3Blocks	ST	vare	4958
rust2	A	Ped	ADD	6812.5	0.7642	3Blocks	ST	vare	1780

Table S2 continued from previous page

Trait	Matrice	GenoSet	Model	AIC	h2	PhenoSet	Model2	Variance	Value
angbranch	Acor	Ped	ADD	7246.86	0.5457	3Blocks	ST	vare	1025
budburst	Acor	Ped	ADD	8977.2	0.5811	3Blocks	ST	vare	367.4
circ2	Acor	Ped	ADD	8641.08	0.747	3Blocks	ST	vare	6011
height1	Acor	Ped	ADD	9432.84	0.6523	3Blocks	ST	vare	356.1
height2	Acor	Ped	ADD	7427.49	0.7382	3Blocks	ST	vare	902.5
rust1	Acor	Ped	ADD	11835.47	0.5724	3Blocks	ST	vare	4664
rust2	Acor	Ped	ADD	6807.11	0.7967	3Blocks	ST	vare	1571
angbranch	G	7K	ADD	7235.51	0.4541	3Blocks	ST	vare	1192
budburst	G	7K	ADD	8890.78	0.5951	3Blocks	ST	vare	372.4
circ2	G	7K	ADD	8627.68	0.5134	3Blocks	ST	vare	10180
height1	G	7K	ADD	9409.86	0.3987	3Blocks	ST	vare	537.7
height2	G	7K	ADD	7403.67	0.5103	3Blocks	ST	vare	1468
rust1	G	7K	ADD	11779.5	0.5196	3Blocks	ST	vare	5264
rust2	G	7K	ADD	6803.73	0.4807	3Blocks	ST	vare	3308
angbranch	Gw1	7K	ADD	6959.27	0.721	3Blocks	ST	vare	534
budburst	Gw1	7K	ADD	8528.86	0.704	3Blocks	ST	vare	215.4
circ2	Gw1	7K	ADD	8394.29	0.6748	3Blocks	ST	vare	5601
height1	Gw1	7K	ADD	9161.51	0.5525	3Blocks	ST	vare	363
height2	Gw1	7K	ADD	7168.52	0.6826	3Blocks	ST	vare	790.6
rust1	Gw1	7K	ADD	11451.84	0.6309	3Blocks	ST	vare	3329
rust2	Gw1	7K	ADD	6595.57	0.6427	3Blocks	ST	vare	1780
angbranch	Gw2	7K	ADD	6760.27	0.8542	3Blocks	ST	vare	371.1
budburst	Gw2	7K	ADD	8321.29	0.7784	3Blocks	ST	vare	171.6
circ2	Gw2	7K	ADD	8233.62	0.7686	3Blocks	ST	vare	4182
height1	Gw2	7K	ADD	8996.43	0.6533	3Blocks	ST	vare	302
height2	Gw2	7K	ADD	6999.76	0.78	3Blocks	ST	vare	571.3
rust1	Gw2	7K	ADD	11269.7	0.7171	3Blocks	ST	vare	2782
rust2	Gw2	7K	ADD	6470.09	0.7351	3Blocks	ST	vare	1371
angbranch	Gw3	7K	ADD	6660.21	0.8912	3Blocks	ST	vare	312.3
budburst	Gw3	7K	ADD	8245.59	0.8254	3Blocks	ST	vare	160.3
circ2	Gw3	7K	ADD	8151.84	0.8241	3Blocks	ST	vare	3656
height1	Gw3	7K	ADD	8922.83	0.7251	3Blocks	ST	vare	281.6
height2	Gw3	7K	ADD	6917.38	0.8403	3Blocks	ST	vare	500.8
rust1	Gw3	7K	ADD	11195.62	0.7591	3Blocks	ST	vare	2629
rust2	Gw3	7K	ADD	6410.86	0.7917	3Blocks	ST	vare	1231
angbranch	G	250K	ADD	7191.25	0.9987	6Blocks	ST	vare	2.134
budburst	G	250K	ADD	9104.5	0.9928	6Blocks	ST	vare	5.546
circ2	G	250K	ADD	8682.62	0.9906	6Blocks	ST	vare	162
height1	G	250K	ADD	9314.56	0.8516	6Blocks	ST	vare	99.34
height2	G	250K	ADD	7434.61	0.9962	6Blocks	ST	vare	8.999
rust1	G	250K	ADD	11762.95	0.9964	6Blocks	ST	vare	28.33
rust2	G	250K	ADD	6820.24	0.9983	6Blocks	ST	vare	9.016
angbranch	Gw1	250K	ADD	6462.08	0.9967	6Blocks	ST	vare	1.866

Table S2 continued from previous page

Trait	Matrice	GenoSet	Model	AIC	h2	PhenoSet	Model2	Variance	Value
budburst	Gw1	250K	ADD	7967.07	0.9891	6Blocks	ST	vare	2.978
circ2	Gw1	250K	ADD	7891.64	0.9969	6Blocks	ST	vare	18.68
height1	Gw1	250K	ADD	8164.51	0.9896	6Blocks	ST	vare	2.539
height2	Gw1	250K	ADD	6658.75	0.9948	6Blocks	ST	vare	4.308
rust1	Gw1	250K	ADD	10625.4	0.9958	6Blocks	ST	vare	11.6
rust2	Gw1	250K	ADD	6202.78	0.9948	6Blocks	ST	vare	9.214
angbranch	Gw2	250K	ADD	6028.59	0.9866	6Blocks	ST	vare	5.379
budburst	Gw2	250K	ADD	7326.53	0.993	6Blocks	ST	vare	1.364
circ2	Gw2	250K	ADD	7456.64	0.9952	6Blocks	ST	vare	20.49
height1	Gw2	250K	ADD	7469.71	0.9972	6Blocks	ST	vare	0.4453
height2	Gw2	250K	ADD	6206.76	0.9951	6Blocks	ST	vare	2.647
rust1	Gw2	250K	ADD	9972.4	0.9972	6Blocks	ST	vare	5.543
rust2	Gw2	250K	ADD	5826.7	0.9952	6Blocks	ST	vare	5.465
angbranch	Gw3	250K	ADD	5721.72	0.9995	6Blocks	ST	vare	0.2091
budburst	Gw3	250K	ADD	6891.37	0.9993	6Blocks	ST	vare	0.1448
circ2	Gw3	250K	ADD	7158.87	0.9987	6Blocks	ST	vare	5.408
height1	Gw3	250K	ADD	7054.08	0.9993	6Blocks	ST	vare	0.1255
height2	Gw3	250K	ADD	5931.04	0.9995	6Blocks	ST	vare	0.281
rust1	Gw3	250K	ADD	9545.09	0.9994	6Blocks	ST	vare	1.39
rust2	Gw3	250K	ADD	5601.46	0.9994	6Blocks	ST	vare	0.8209
angbranch	G	50K	ADD	7111.71	0.9987	6Blocks	ST	vare	2.014
budburst	G	50K	ADD	8934.42	0.9156	6Blocks	ST	vare	58.12
circ2	G	50K	ADD	8518.67	0.9983	6Blocks	ST	vare	24.34
height1	G	50K	ADD	9161.61	0.9974	6Blocks	ST	vare	1.594
height2	G	50K	ADD	7285.82	0.9989	6Blocks	ST	vare	2.32
rust1	G	50K	ADD	11665.46	0.9973	6Blocks	ST	vare	20.66
rust2	G	50K	ADD	6728.51	0.9957	6Blocks	ST	vare	20.23
angbranch	Gw1	50K	ADD	6411.31	0.997	6Blocks	ST	vare	1.645
budburst	Gw1	50K	ADD	7952.5	0.9425	6Blocks	ST	vare	16.68
circ2	Gw1	50K	ADD	7803.77	0.9969	6Blocks	ST	vare	16.37
height1	Gw1	50K	ADD	8091.23	0.9964	6Blocks	ST	vare	0.826
height2	Gw1	50K	ADD	6586.62	0.9971	6Blocks	ST	vare	2.209
rust1	Gw1	50K	ADD	10587.21	0.9909	6Blocks	ST	vare	26.47
rust2	Gw1	50K	ADD	6153.06	0.9954	6Blocks	ST	vare	7.766
angbranch	Gw2	50K	ADD	6020.07	0.9895	6Blocks	ST	vare	5.326
budburst	Gw2	50K	ADD	7385.67	0.9659	6Blocks	ST	vare	8.038
circ2	Gw2	50K	ADD	7438.16	0.9868	6Blocks	ST	vare	58.9
height1	Gw2	50K	ADD	7486.42	0.9938	6Blocks	ST	vare	1.183
height2	Gw2	50K	ADD	6218.73	0.9883	6Blocks	ST	vare	7.219
rust1	Gw2	50K	ADD	10010.6	0.98	6Blocks	ST	vare	52.51
rust2	Gw2	50K	ADD	5831.55	0.989	6Blocks	ST	vare	14.75
angbranch	Gw3	50K	ADD	5796.11	0.9743	6Blocks	ST	vare	14.55
budburst	Gw3	50K	ADD	6948.46	0.9993	6Blocks	ST	vare	0.189

Table S2 continued from previous page

Trait	Matrice	GenoSet	Model	AIC	h2	PhenoSet	Model2	Variance	Value
circ2	Gw3	50K	ADD	7160.87	0.9996	6Blocks	ST	vare	2.11
height1	Gw3	50K	ADD	7109.33	0.975	6Blocks	ST	vare	5.429
height2	Gw3	50K	ADD	5961.46	0.9995	6Blocks	ST	vare	0.3525
rust1	Gw3	50K	ADD	9550.5	0.9995	6Blocks	ST	vare	1.653
rust2	Gw3	50K	ADD	5577.39	0.9997	6Blocks	ST	vare	0.5207
angbranch	G	7K_homo	ADD	7092.31	0.735	6Blocks	ST	vare	477.6
budburst	G	7K_homo	ADD	8842.08	0.6414	6Blocks	ST	vare	267.6
circ2	G	7K_homo	ADD	8472.27	0.754	6Blocks	ST	vare	3987
height1	G	7K_homo	ADD	9148.32	0.5498	6Blocks	ST	vare	304.8
height2	G	7K_homo	ADD	7260.14	0.6818	6Blocks	ST	vare	722.4
rust1	G	7K_homo	ADD	11617.91	0.6942	6Blocks	ST	vare	2751
rust2	G	7K_homo	ADD	6690.41	0.8349	6Blocks	ST	vare	926.5
angbranch	Gw1	7K_homo	ADD	6527.16	0.9996	6Blocks	ST	vare	0.4697
budburst	Gw1	7K_homo	ADD	8282	0.882	6Blocks	ST	vare	75.02
circ2	Gw1	7K_homo	ADD	7973.03	0.9995	6Blocks	ST	vare	5.446
height1	Gw1	7K_homo	ADD	8604.69	0.9019	6Blocks	ST	vare	63.57
height2	Gw1	7K_homo	ADD	6827.22	0.976	6Blocks	ST	vare	44.08
rust1	Gw1	7K_homo	ADD	10922.78	0.96	6Blocks	ST	vare	309.8
rust2	Gw1	7K_homo	ADD	6201.85	0.9992	6Blocks	ST	vare	2.611
angbranch	Gw2	7K_homo	ADD	6075.62	0.9997	6Blocks	ST	vare	0.2892
budburst	Gw2	7K_homo	ADD	7897.71	0.9506	6Blocks	ST	vare	35.98
circ2	Gw2	7K_homo	ADD	7553.23	0.9997	6Blocks	ST	vare	3.129
height1	Gw2	7K_homo	ADD	8086.45	0.9584	6Blocks	ST	vare	25.99
height2	Gw2	7K_homo	ADD	6407.07	0.9997	6Blocks	ST	vare	0.432
rust1	Gw2	7K_homo	ADD	10364.86	0.9862	6Blocks	ST	vare	99.79
rust2	Gw2	7K_homo	ADD	5895.27	0.9994	6Blocks	ST	vare	1.706
angbranch	Gw3	7K_homo	ADD	5785.27	0.9999	6Blocks	ST	vare	0.1467
budburst	Gw3	7K_homo	ADD	7592.22	0.9677	6Blocks	ST	vare	23.01
circ2	Gw3	7K_homo	ADD	7280.74	0.9999	6Blocks	ST	vare	1.32
height1	Gw3	7K_homo	ADD	7747.73	0.9716	6Blocks	ST	vare	17.15
height2	Gw3	7K_homo	ADD	6155.38	0.9855	6Blocks	ST	vare	19.35
rust1	Gw3	7K_homo	ADD	9994.92	0.9932	6Blocks	ST	vare	47.58
rust2	Gw3	7K_homo	ADD	5679.46	0.9998	6Blocks	ST	vare	0.5313
angbranch	A	Ped	ADD	7113.63	0.6465	6Blocks	ST	vare	680.1
budburst	A	Ped	ADD	8961.26	0.606	6Blocks	ST	vare	344.9
circ2	A	Ped	ADD	8480.9	0.7986	6Blocks	ST	vare	3791
height1	A	Ped	ADD	9175.13	0.672	6Blocks	ST	vare	262.3
height2	A	Ped	ADD	7270.52	0.7433	6Blocks	ST	vare	680.6
rust1	A	Ped	ADD	11698.41	0.594	6Blocks	ST	vare	3907
rust2	A	Ped	ADD	6715.81	0.9217	6Blocks	ST	vare	565.6
angbranch	Acor	Ped	ADD	7109.71	0.6572	6Blocks	ST	vare	663.1
budburst	Acor	Ped	ADD	8899.47	0.5946	6Blocks	ST	vare	330.5
circ2	Acor	Ped	ADD	8479.19	0.8093	6Blocks	ST	vare	3630

Table S2 continued from previous page

Trait	Matrice	GenoSet	Model	AIC	h2	PhenoSet	Model2	Variance	Value
height1	Acor	Ped	ADD	9164.54	0.7148	6Blocks	ST	vare	233.6
height2	Acor	Ped	ADD	7266.34	0.7507	6Blocks	ST	vare	663.5
rust1	Acor	Ped	ADD	11679.32	0.6355	6Blocks	ST	vare	3550
rust2	Acor	Ped	ADD	6709.44	0.9388	6Blocks	ST	vare	448.8
angbranch	G	7K	ADD	7101.78	0.4891	6Blocks	ST	vare	918.8
budburst	G	7K	ADD	8805.66	0.6204	6Blocks	ST	vare	329.5
circ2	G	7K	ADD	8468.77	0.5894	6Blocks	ST	vare	7092
height1	G	7K	ADD	9139.3	0.4379	6Blocks	ST	vare	396.4
height2	G	7K	ADD	7251.78	0.5371	6Blocks	ST	vare	1104
rust1	G	7K	ADD	11587.63	0.6007	6Blocks	ST	vare	3962
rust2	G	7K	ADD	6697.58	0.5711	6Blocks	ST	vare	2428
angbranch	Gw1	7K	ADD	6785.52	0.7664	6Blocks	ST	vare	364.2
budburst	Gw1	7K	ADD	8425.28	0.7238	6Blocks	ST	vare	186.1
circ2	Gw1	7K	ADD	8204.91	0.7001	6Blocks	ST	vare	3898
height1	Gw1	7K	ADD	8869.61	0.5837	6Blocks	ST	vare	261.3
height2	Gw1	7K	ADD	7015.74	0.6668	6Blocks	ST	vare	629.7
rust1	Gw1	7K	ADD	11187.37	0.7005	6Blocks	ST	vare	2298
rust2	Gw1	7K	ADD	6435.88	0.7139	6Blocks	ST	vare	1152
angbranch	Gw2	7K	ADD	6555.81	0.8925	6Blocks	ST	vare	239.6
budburst	Gw2	7K	ADD	8215	0.7923	6Blocks	ST	vare	150.1
circ2	Gw2	7K	ADD	8032.93	0.7944	6Blocks	ST	vare	2854
height1	Gw2	7K	ADD	8679.73	0.6933	6Blocks	ST	vare	209.2
height2	Gw2	7K	ADD	6858.89	0.7531	6Blocks	ST	vare	477.6
rust1	Gw2	7K	ADD	10982.71	0.7839	6Blocks	ST	vare	1877
rust2	Gw2	7K	ADD	6304.13	0.7873	6Blocks	ST	vare	910.9
angbranch	Gw3	7K	ADD	6438.42	0.9202	6Blocks	ST	vare	204.1
budburst	Gw3	7K	ADD	8138.07	0.8333	6Blocks	ST	vare	141.4
circ2	Gw3	7K	ADD	7949.29	0.8509	6Blocks	ST	vare	2498
height1	Gw3	7K	ADD	8593.27	0.7578	6Blocks	ST	vare	193.9
height2	Gw3	7K	ADD	6781.64	0.8273	6Blocks	ST	vare	415.3
rust1	Gw3	7K	ADD	10890.69	0.8159	6Blocks	ST	vare	1753
rust2	Gw3	7K	ADD	6246.2	0.8274	6Blocks	ST	vare	830.6
angbranch	G	250K	ADD_DOM	7205.1	0.4724	6Blocks	ST	vare	91.2
budburst	G	250K	ADD_DOM	9106.38	0.4967	6Blocks	ST	vare	5.055
circ2	G	250K	ADD_DOM	8683.45	0.4968	6Blocks	ST	vare	111.2
height1	G	250K	ADD_DOM	9288.4	0.4987	6Blocks	ST	vare	1.637
height2	G	250K	ADD_DOM	7438	0.4968	6Blocks	ST	vare	15.36
rust1	G	250K	ADD_DOM	11764.64	0.4984	6Blocks	ST	vare	24.64
rust2	G	250K	ADD_DOM	6823.68	0.4983	6Blocks	ST	vare	17.48
angbranch	G	50K	ADD_DOM	7114.19	0.4992	6Blocks	ST	vare	2.542
budburst	G	50K	ADD_DOM	8936.42	0.4583	6Blocks	ST	vare	57.47
circ2	G	50K	ADD_DOM	8523.19	0.4975	6Blocks	ST	vare	72.56
height1	G	50K	ADD_DOM	9165.18	0.4973	6Blocks	ST	vare	3.372

Table S2 continued from previous page

Trait	Matrice	GenoSet	Model	AIC	h2	PhenoSet	Model2	Variance	Value
height2	G	50K	ADD_DOM	7290.76	0.4978	6Blocks	ST	vare	8.847
rust1	G	50K	ADD_DOM	11667.79	0.4984	6Blocks	ST	vare	24.27
rust2	G	50K	ADD_DOM	6734.09	0.491	6Blocks	ST	vare	84.19
angbranch	G	7K_homo	ADD_DOM	7094.31	0.369	6Blocks	ST	vare	473.3
budburst	G	7K_homo	ADD_DOM	8844.08	0.3207	6Blocks	ST	vare	267.6
circ2	G	7K_homo	ADD_DOM	8474.27	0.3774	6Blocks	ST	vare	3977
height1	G	7K_homo	ADD_DOM	9150.32	0.2749	6Blocks	ST	vare	304.8
height2	G	7K_homo	ADD_DOM	7262.14	0.3407	6Blocks	ST	vare	723.6
rust1	G	7K_homo	ADD_DOM	11619.91	0.3471	6Blocks	ST	vare	2751
rust2	G	7K_homo	ADD_DOM	6692.42	0.4194	6Blocks	ST	vare	907.6
angbranch	A	Ped	ADD_DOM	7115.63	0.3235	6Blocks	ST	vare	679.3
budburst	A	Ped	ADD_DOM	8963.26	0.3029	6Blocks	ST	vare	345
circ2	A	Ped	ADD_DOM	8482.9	0.3994	6Blocks	ST	vare	3786
height1	A	Ped	ADD_DOM	9177.13	0.3356	6Blocks	ST	vare	262.8
height2	A	Ped	ADD_DOM	7272.52	0.3713	6Blocks	ST	vare	682.1
rust1	A	Ped	ADD_DOM	11700.41	0.2974	6Blocks	ST	vare	3902
rust2	A	Ped	ADD_DOM	6717.85	0.4662	6Blocks	ST	vare	492.8
angbranch	Acor	Ped	ADD_DOM	7111.72	0.3301	6Blocks	ST	vare	658.7
budburst	Acor	Ped	ADD_DOM	8901.47	0.2972	6Blocks	ST	vare	330.7
circ2	Acor	Ped	ADD_DOM	8481.19	0.4041	6Blocks	ST	vare	3650
height1	Acor	Ped	ADD_DOM	9166.54	0.3588	6Blocks	ST	vare	231.7
height2	Acor	Ped	ADD_DOM	7268.34	0.3747	6Blocks	ST	vare	666.2
rust1	Acor	Ped	ADD_DOM	11681.32	0.3173	6Blocks	ST	vare	3557
rust2	Acor	Ped	ADD_DOM	6711.51	0.474	6Blocks	ST	vare	385.7
angbranch	G	7K	ADD_DOM	7104.62	0.2424	6Blocks	ST	vare	923
budburst	G	7K	ADD_DOM	8806.77	0.3123	6Blocks	ST	vare	326.6
circ2	G	7K	ADD_DOM	8470.65	0.2943	6Blocks	ST	vare	7085
height1	G	7K	ADD_DOM	9141.99	0.2176	6Blocks	ST	vare	397.1
height2	G	7K	ADD_DOM	7253.57	0.2685	6Blocks	ST	vare	1102
rust1	G	7K	ADD_DOM	11589.85	0.3006	6Blocks	ST	vare	3953
rust2	G	7K	ADD_DOM	6699.45	0.2847	6Blocks	ST	vare	2428
rust1	G	50K	ADD	45920.8	0.944	6Blocks	MT	vare	429.5
rust2	G	50K	ADD	45920.8	0.8158	6Blocks	MT	vare	827.5
circ2	G	50K	ADD	45920.8	0.9326	6Blocks	MT	vare	994.6
height1	G	50K	ADD	45920.8	0.9285	6Blocks	MT	vare	43.89
height2	G	50K	ADD	45920.8	0.9461	6Blocks	MT	vare	116.6
angbranch	G	50K	ADD	45920.8	0.9633	6Blocks	MT	vare	56.4
budburst	G	50K	ADD	45920.8	0.8393	6Blocks	MT	vare	108.6
rust1	G	7K_homo	ADD	45685.92	0.6758	6Blocks	MT	vare	2875
rust2	G	7K_homo	ADD	45685.92	0.5608	6Blocks	MT	vare	2133
circ2	G	7K_homo	ADD	45685.92	0.6605	6Blocks	MT	vare	5557
height1	G	7K_homo	ADD	45685.92	0.5204	6Blocks	MT	vare	319.2
height2	G	7K_homo	ADD	45685.92	0.6211	6Blocks	MT	vare	900.9

Table S2 continued from previous page

Trait	Matrice	GenoSet	Model	AIC	h2	PhenoSet	Model2	Variance	Value
angbranch	G	7K_homo	ADD	45685.92	0.7337	6Blocks	MT	vare	478.8
budburst	G	7K_homo	ADD	45685.92	0.6373	6Blocks	MT	vare	270.3
rust1	A	Ped	ADD	45934.44	0.6422	6Blocks	MT	vare	3550
rust2	A	Ped	ADD	45934.44	0.5984	6Blocks	MT	vare	2208
circ2	A	Ped	ADD	45934.44	0.7467	6Blocks	MT	vare	4808
height1	A	Ped	ADD	45934.44	0.5921	6Blocks	MT	vare	308.8
height2	A	Ped	ADD	45934.44	0.6831	6Blocks	MT	vare	863.2
angbranch	A	Ped	ADD	45934.44	0.6797	6Blocks	MT	vare	626.2
budburst	A	Ped	ADD	45934.44	0.5843	6Blocks	MT	vare	358.4
rust1	Acor	Ped	ADD	45823.36	0.7244	6Blocks	MT	vare	2859
rust2	Acor	Ped	ADD	45823.36	0.604	6Blocks	MT	vare	2171
circ2	Acor	Ped	ADD	45823.36	0.7628	6Blocks	MT	vare	4558
height1	Acor	Ped	ADD	45823.36	0.6296	6Blocks	MT	vare	285.4
height2	Acor	Ped	ADD	45823.36	0.6941	6Blocks	MT	vare	834
angbranch	Acor	Ped	ADD	45823.36	0.7186	6Blocks	MT	vare	567.4
budburst	Acor	Ped	ADD	45823.36	0.5895	6Blocks	MT	vare	332.9
rust1	G	7K	ADD	45626.29	0.5987	6Blocks	MT	vare	3970
rust2	G	7K	ADD	45626.29	0.4757	6Blocks	MT	vare	2782
circ2	G	7K	ADD	45626.29	0.533	6Blocks	MT	vare	8247
height1	G	7K	ADD	45626.29	0.4207	6Blocks	MT	vare	402.4
height2	G	7K	ADD	45626.29	0.4836	6Blocks	MT	vare	1293
angbranch	G	7K	ADD	45626.29	0.4816	6Blocks	MT	vare	927
budburst	G	7K	ADD	45626.29	0.6087	6Blocks	MT	vare	335

1.2 Figures

Figure S1

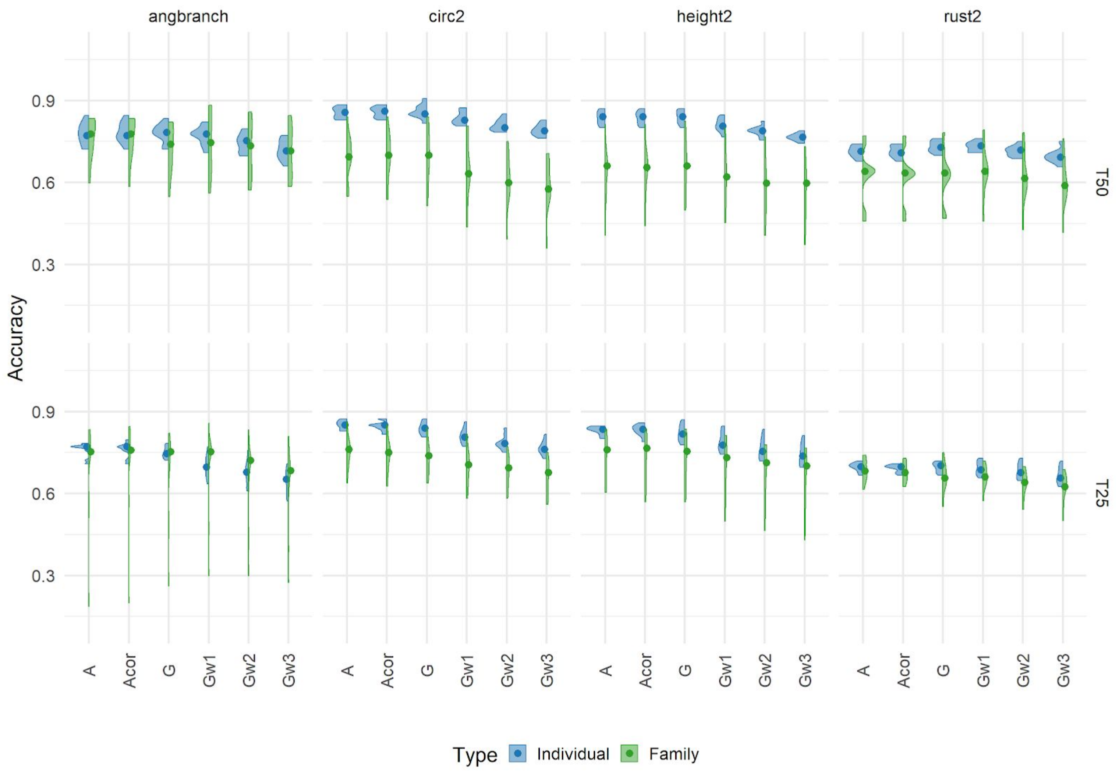

Cross-validation prediction accuracies using an additive model with 7K SNP for five traits grouped by the proportion of individuals (Individual) or families (Family) in training sets 50\% (T50) and 25\% (T25). The color of violinplot showed the sampling strategy: in blue, the individual sampling strategy and in green the family sampling strategy. Each violinplot represented the accuracy of ten repetitions for each relationship matrix. The dot represented the median of each distribution.

Figure S2

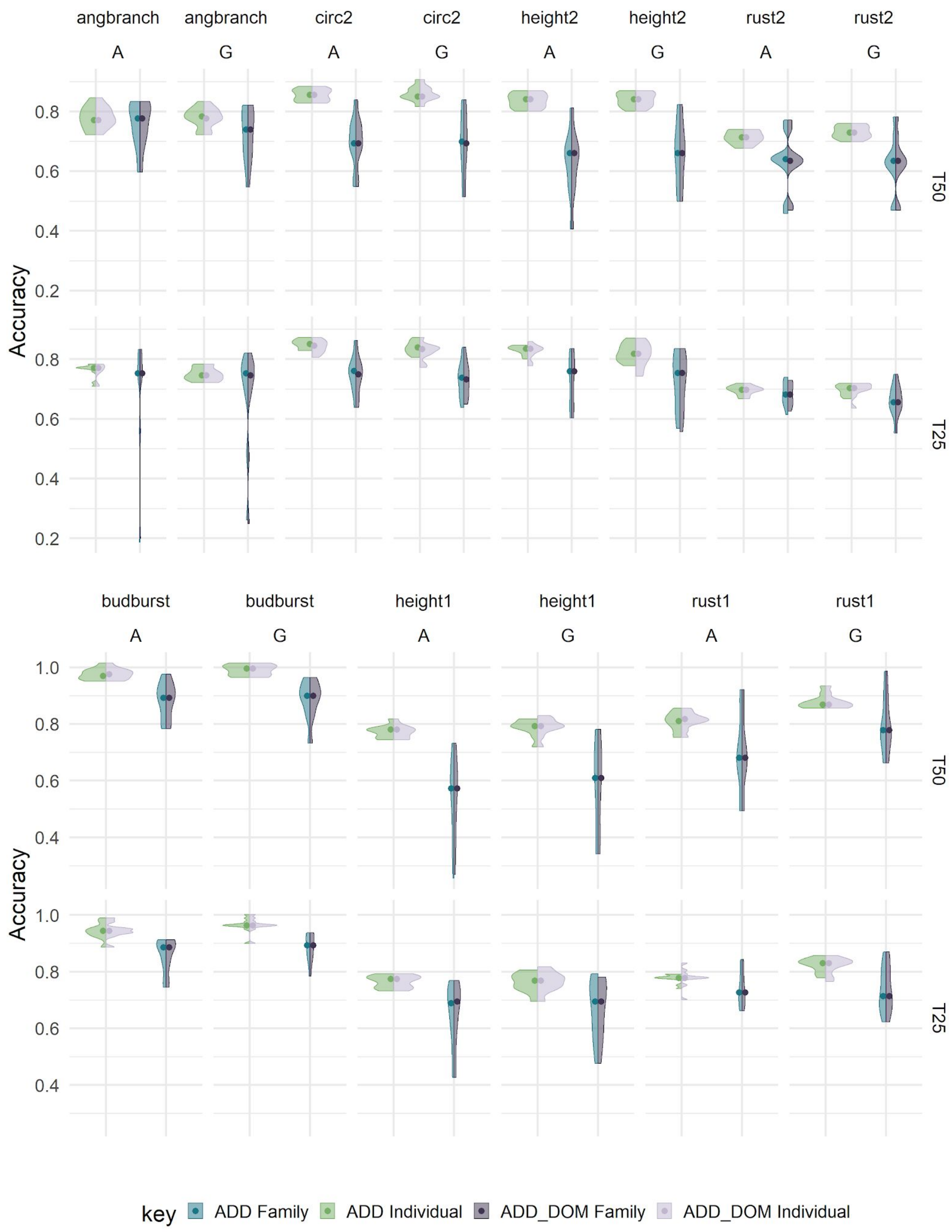

Prediction accuracies using different evaluation models by cross-validation type (horizontal panel) with 7K SNP, and rust1, rust2, angbranch, circ2, budburst, height1 and height2. The violin plots involve additive (ADD) versus additive and dominance (ADD_DOM) single-trait models: with ADD and individual sampling (green), ADD and family sampling (blue), ADD_DOM and individual sampling (pink), ADD_DOM and family sampling (purple).

Figure S3

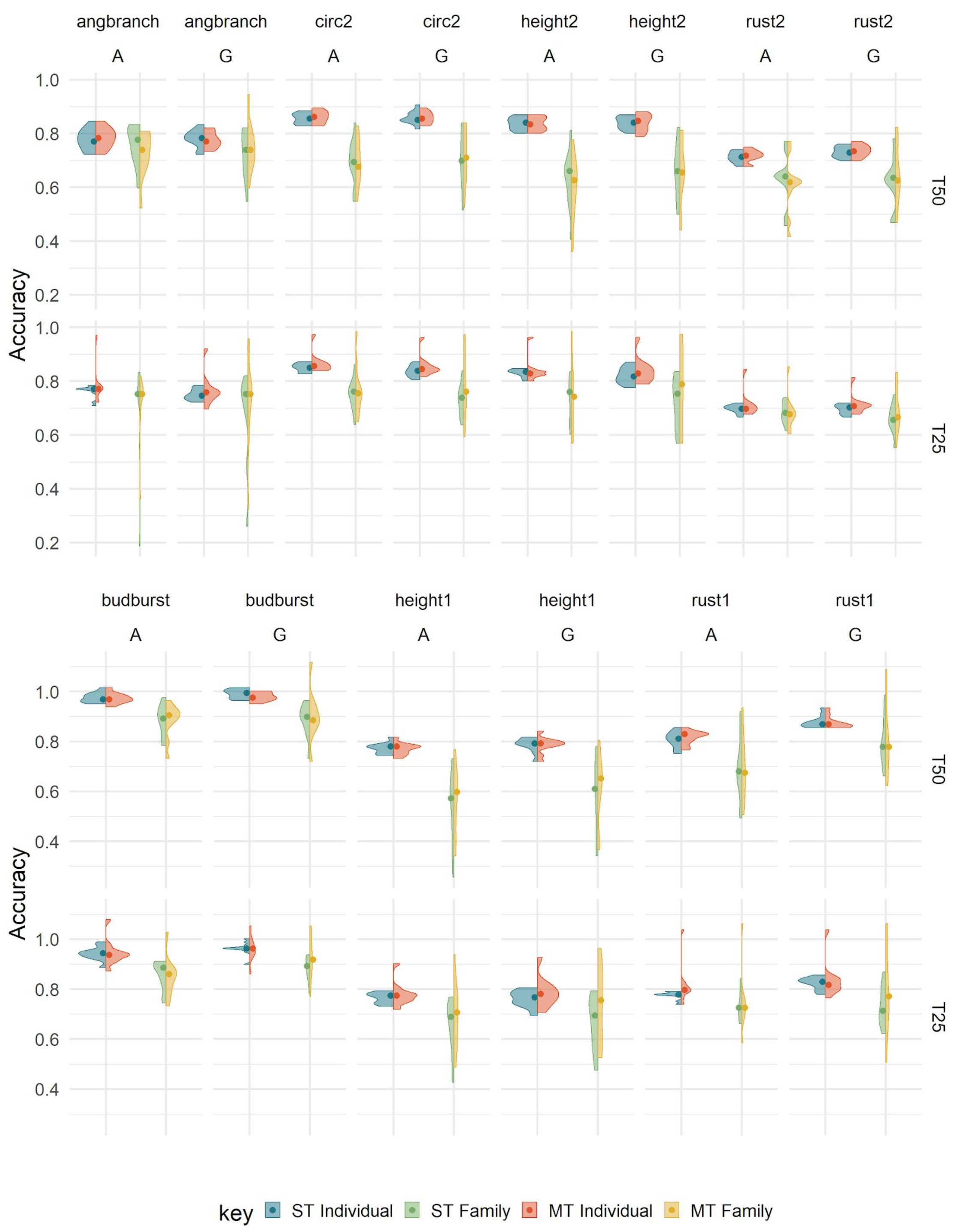

Prediction accuracies using different evaluation models by cross-validation type (horizontal panel) with 7K SNP, and rust1, rust2, angbranch, circ2, budburst, height1 and height2. The violin plots involve single-trait (ST) *versus* multiple-trait (MT) additive models: with ST with individual sampling (blue), ST with family sampling (green), MT with individual sampling (orange), and MT with family sampling (yellow).

Figure S4

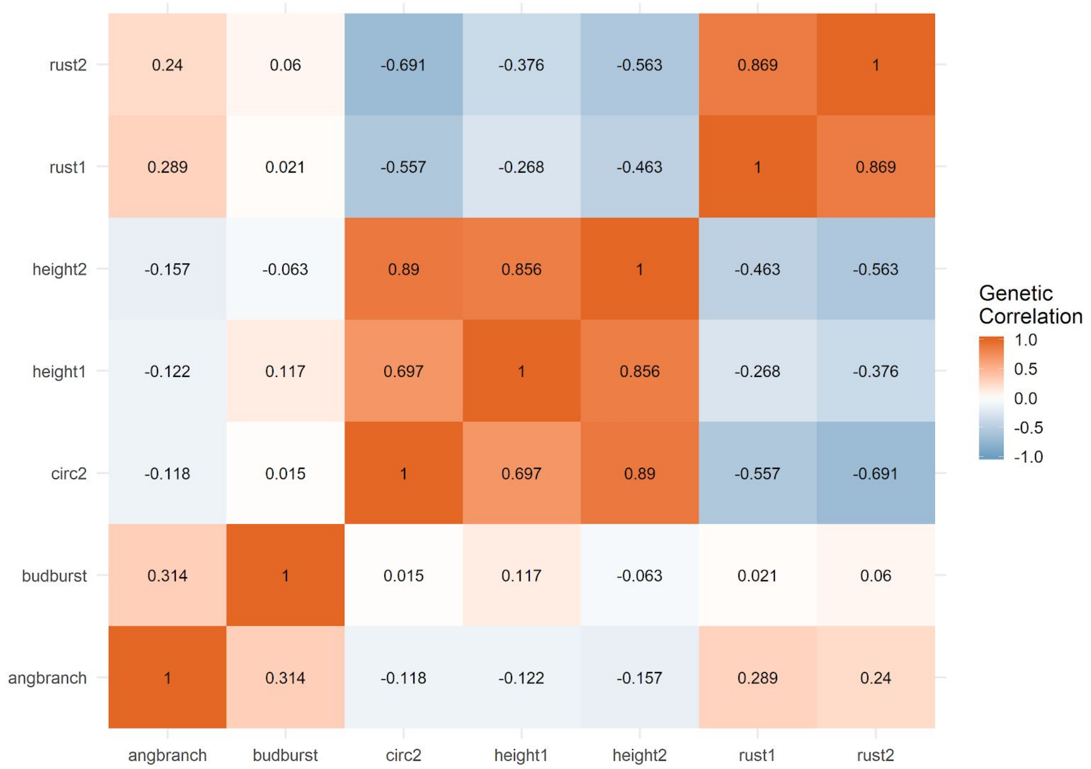

Genetic correlation between traits estimated with all the data set and with an additive model and a genomic relationship matrix build with the 7K chip SNP set.

Figure S5

Marker densification impact on predictive accuracy of a single trait additive model in the TestSet for four genomic relationships matrices (in columns), several training-sampling set in title, and seven different traits organizes in two sets, the first set : height1 (purple), budburst (black) and rust1 (orange), the second set : circ2 (purple), height2 (pink), angbranch (black) and rust2 (orange). The range of accuracies obtained with the pedigree information was represented in each column by the tag Ped. The accuracies distribution in represented by a boxplot.

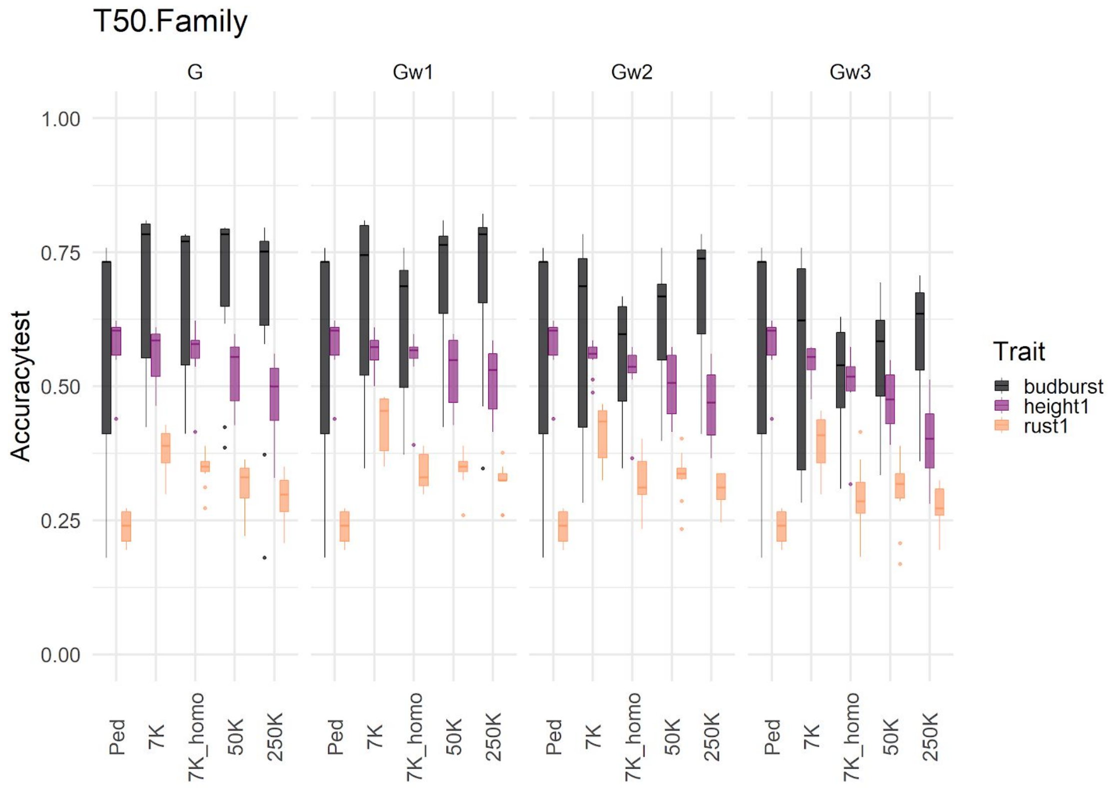

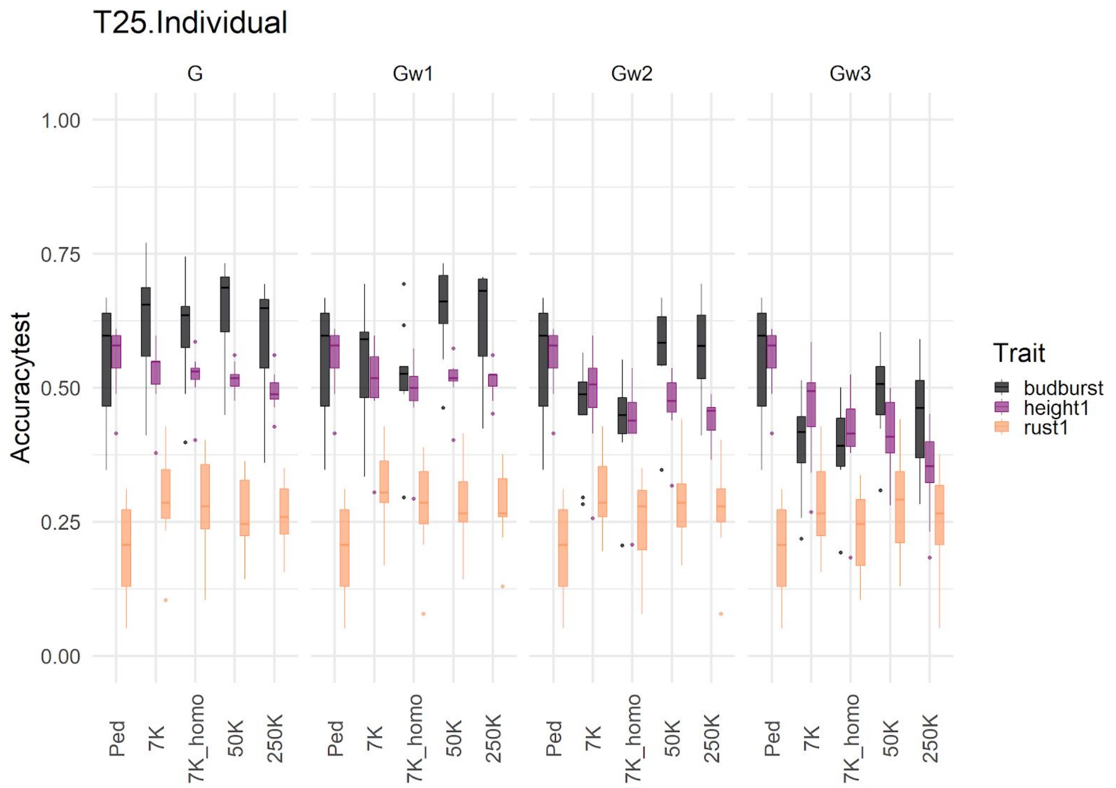
T25.Individual
G
Gw1
Gw2
Gw3
1.00
0.75
0.50
0.25
0.00
Accuracytest
Ped
7K
7K_homo
50K
250K
Trait
budburst
height1
rust1

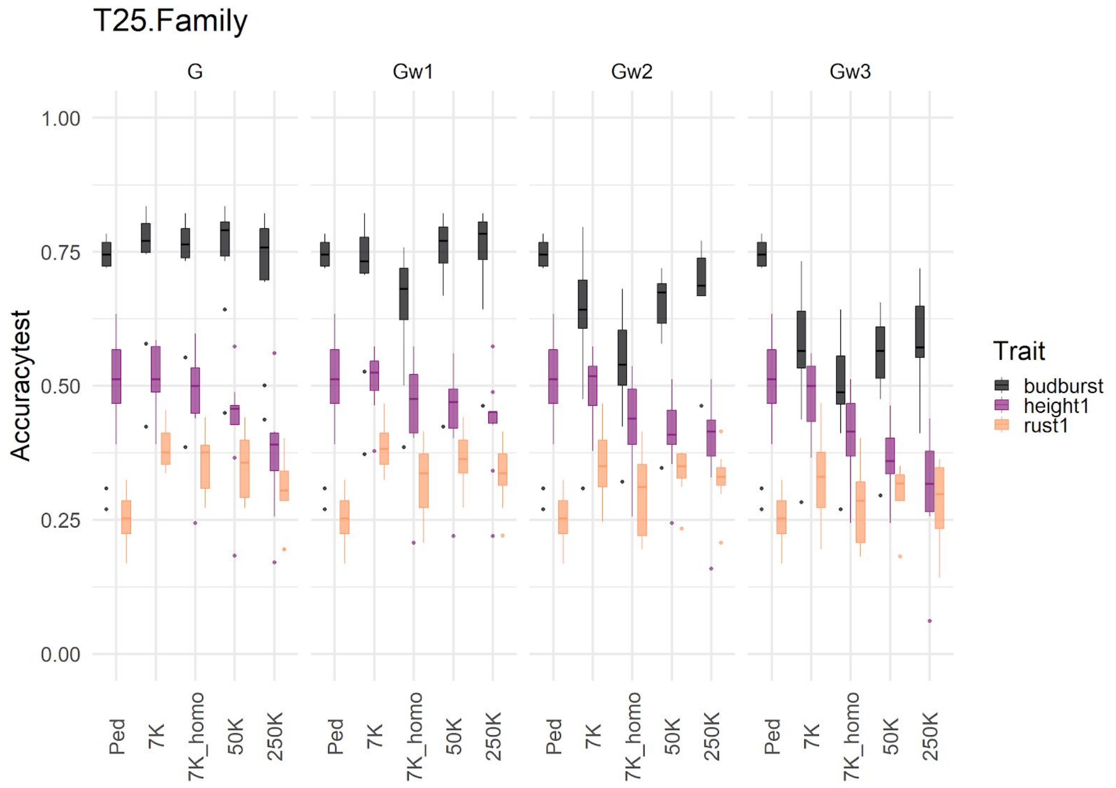
T25.Family
G
Gw1
Gw2
Gw3
1.00
0.75
0.50
0.25
0.00
Accuracytest
Ped
7K
7K_homo
50K
250K
Trait
budburst
height1
rust1

Figure S6

Marker densification impact on predictive accuracy of a single trait additive model in the cross validation for four genomic relationships matrices (in columns), several training-sampling set in title, and seven different traits organizes in two sets, the first set : height1 (purple), budburst (black) and rust1 (orange), the second set : circ2 (purple), height2 (pink), angbranch (black) and rust2 (orange). The range of accuracies obtained with the pedigree information was represented in each column by the tag Ped. The accuracies distribution in represented by a boxplot.

T50.Individual

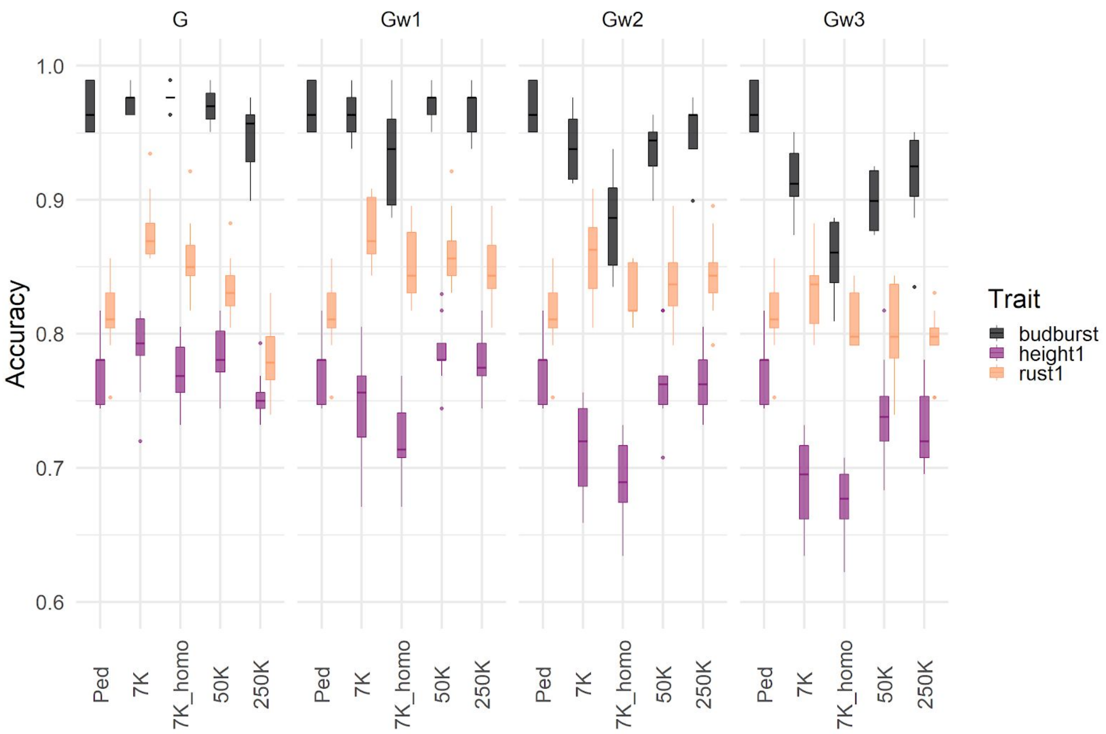

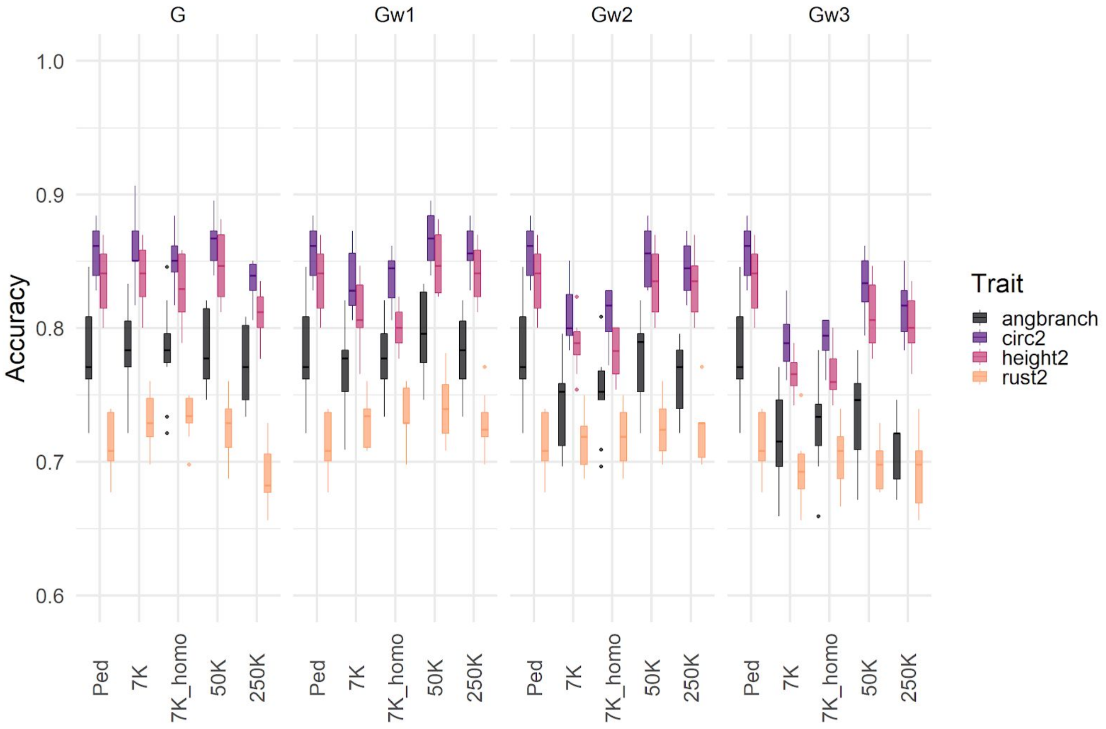

T50.Family

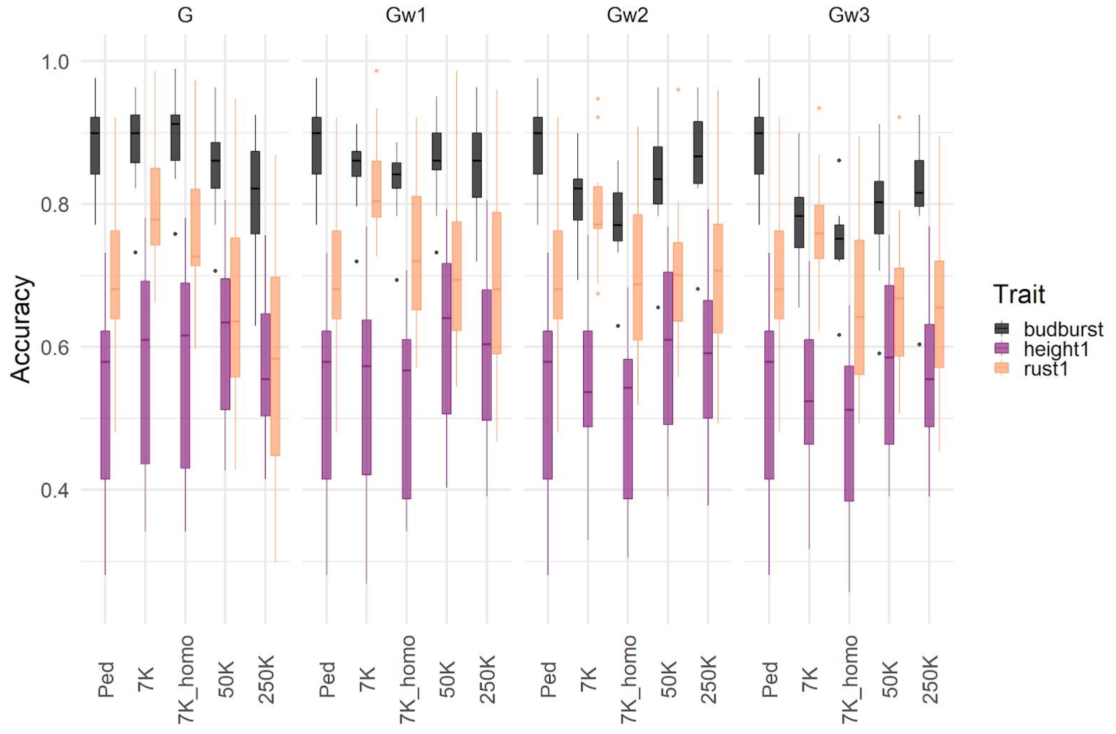
G
Gw1
Gw2
Gw3
1.0
0.8
0.6
0.4
Accuracy
Ped
7K
7K_homo
50K
250K
Trait
budburst
height1
rust1

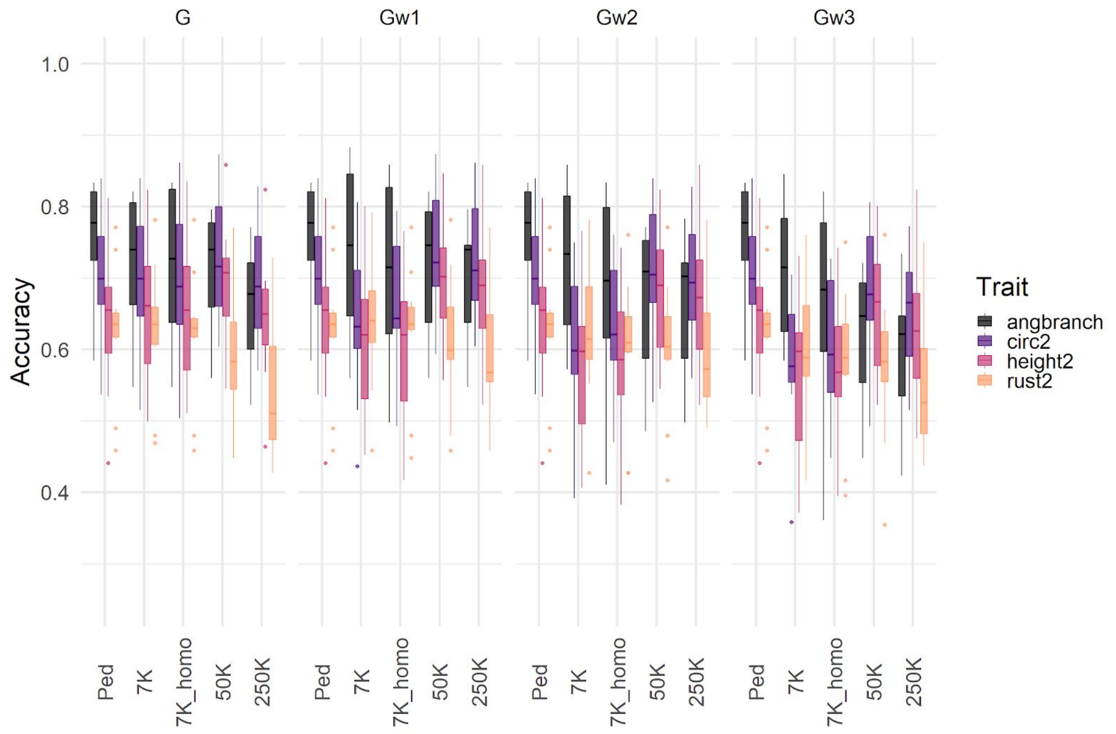
G
Gw1
Gw2
Gw3
1.0
0.8
0.6
0.4
Accuracy
Ped
7K
7K_homo
50K
250K
Trait
angbranch
circ2
height2
rust2

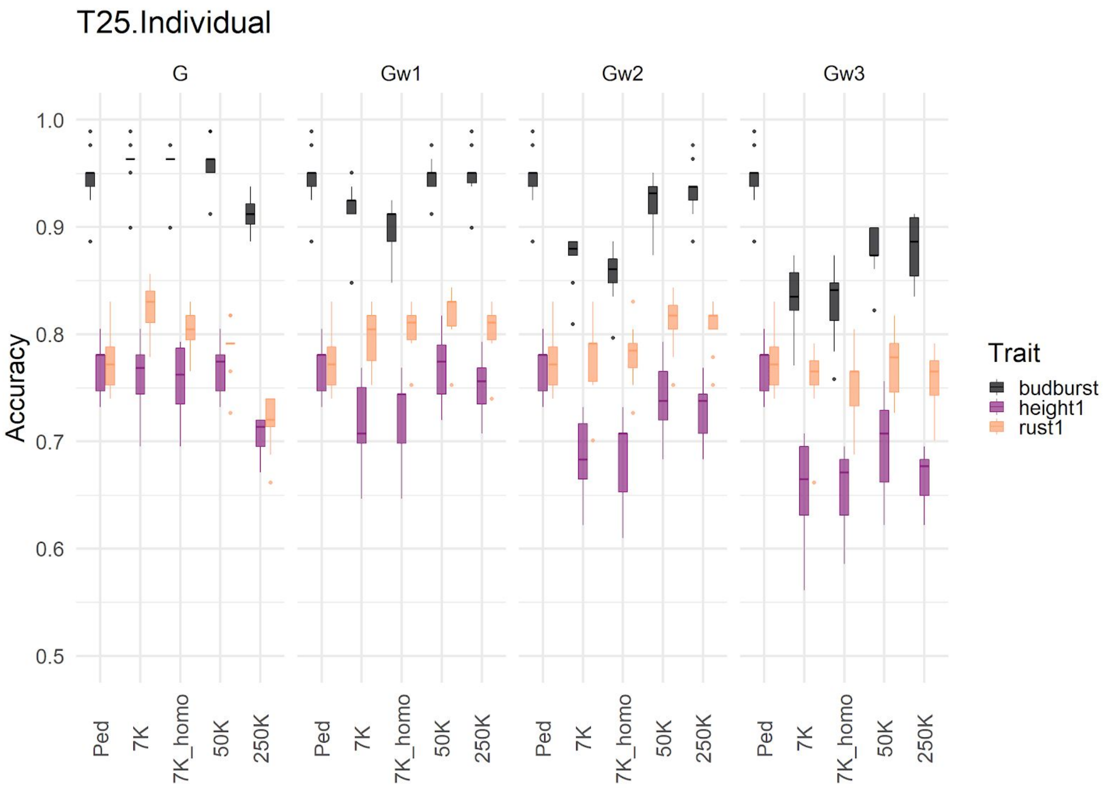
T25.Individual
G
Gw1
Gw2
Gw3
Accuracy
1.0
0.9
0.8
0.7
0.6
0.5
Ped
7K
7K_homo
50K
250K
Trait
budburst
height1
rust1

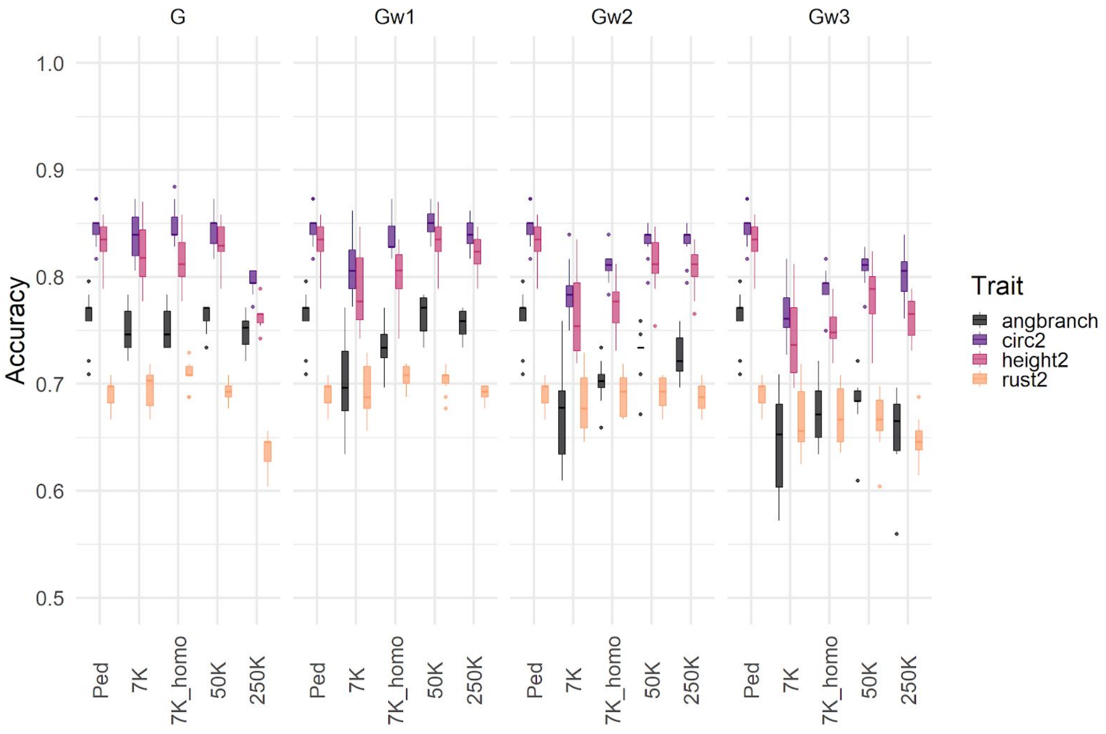
G
Gw1
Gw2
Gw3
Accuracy
1.0
0.9
0.8
0.7
0.6
0.5
Ped
7K
7K_homo
50K
250K
Trait
angbranch
circ2
height2
rust2

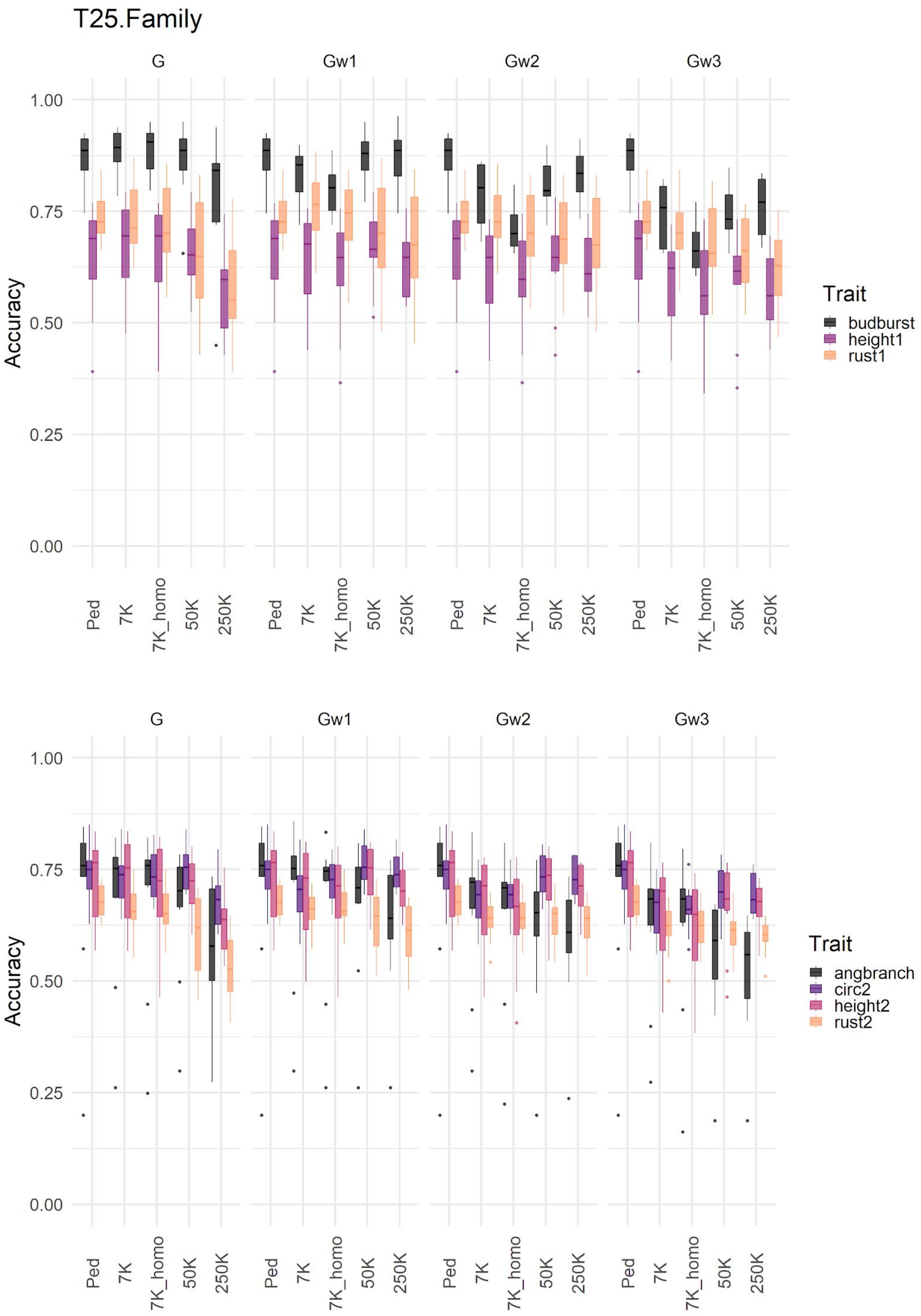
T25.Family
G
Gw1
Gw2
Gw3
Accuracy
1.00
0.75
0.50
0.25
0.00
Ped
7K
7K_homo
50K
250K
Trait
budburst
height1
rust1
angbranch
circ2
height2
rust2

Figure S7

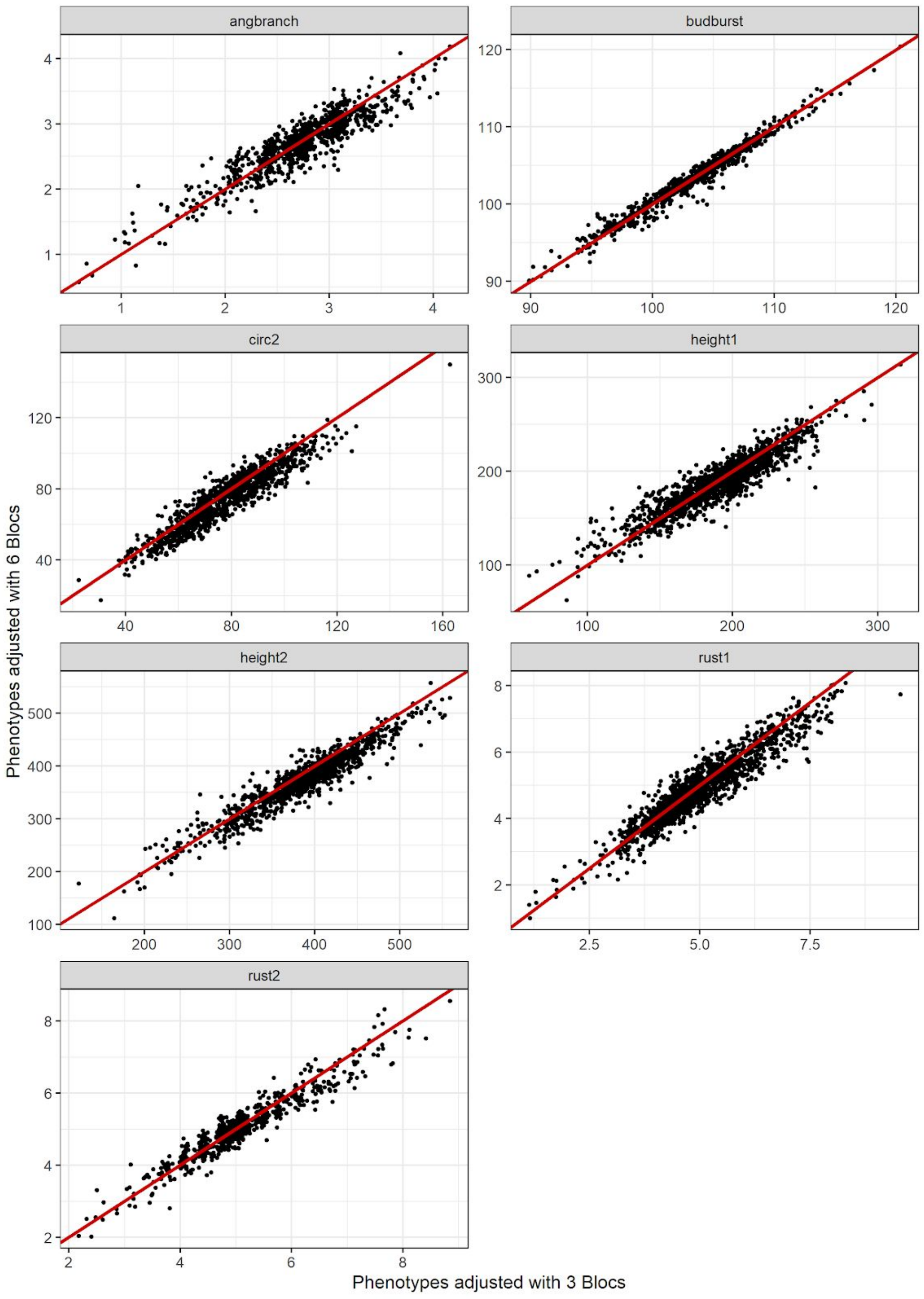

Phenotypes adjusted with 6 Blocks compare to the phenotypes adjusted with 3 Blocks for the seven traits. The read line represent a correlation of 1.

Figure S8

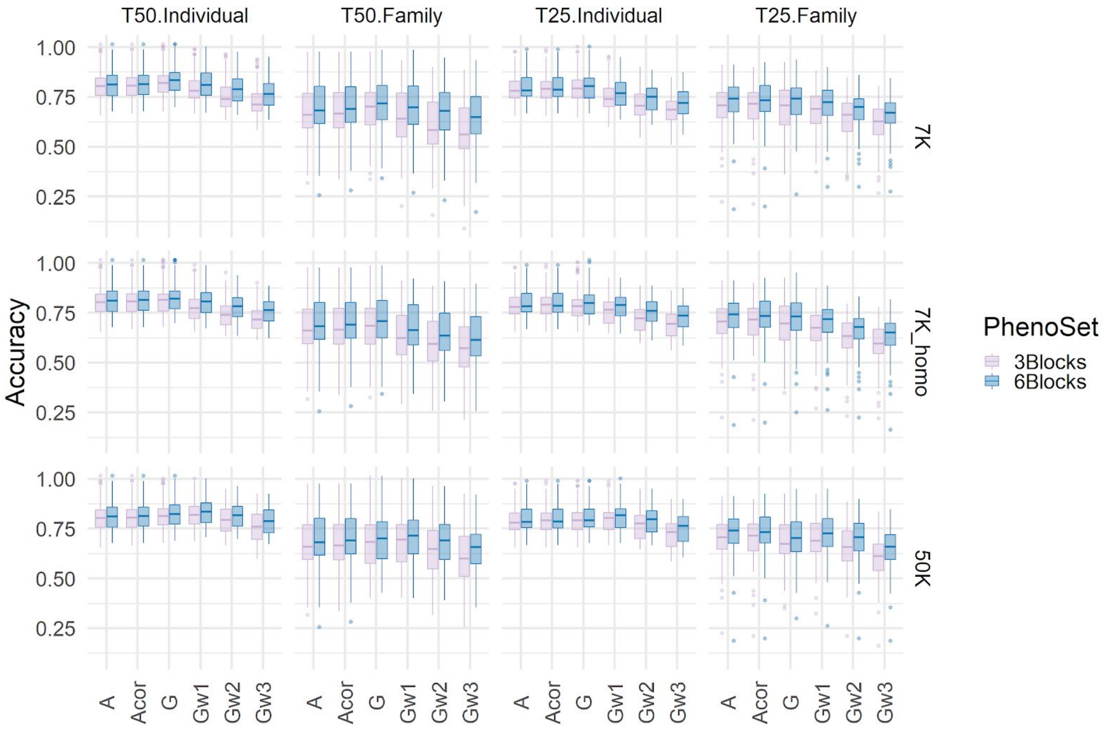

Impact on predictive accuracy of two alternative ways of producing phenotypes, with 3 (pink) and with 6 (blue) replicates in the validation population. Each boxplot represented accuracy values for pooled traits according to relationship matrices used, and were classified by cross-validation strategy in columns and by the number of markers in rows.

Figure S9

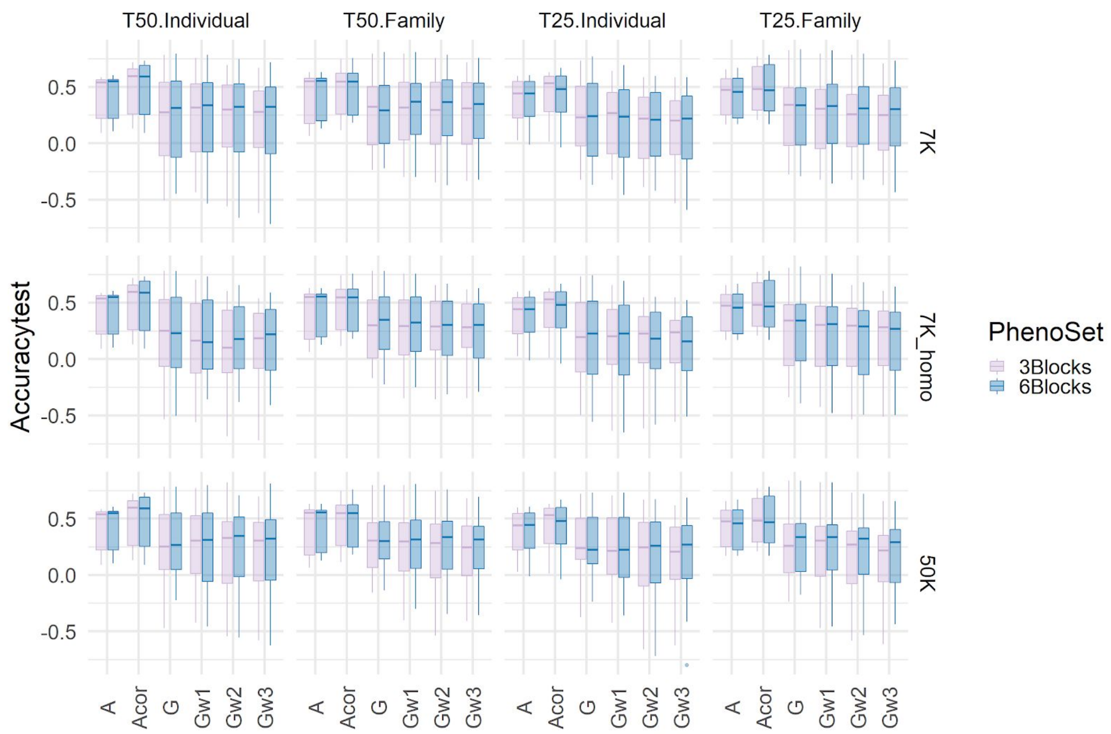

Impact on predictive accuracy of two alternative ways of producing phenotypes, with 3 (pink) and with 6 (blue) replicates in the Test set population. Each boxplot represented accuracy values for pooled traits according to relationship matrices used, and were classified by cross-validation strategy in columns and by the number of markers in rows .

Figure S10

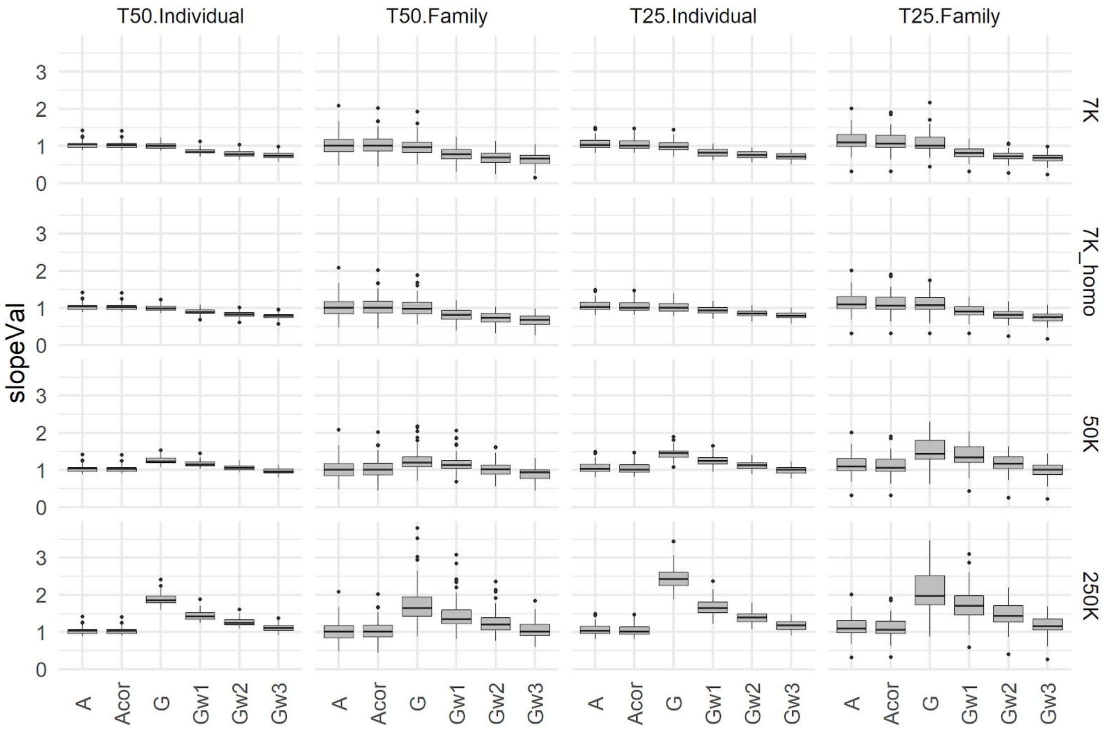

Regression slopes between phenotypes and estimated breeding value in a validation population. Each boxplot represented accuracy values for pooled traits according to relationship matrices used, and were classified by cross-validation strategy in columns and by the number of markers in rows.

Figure S11

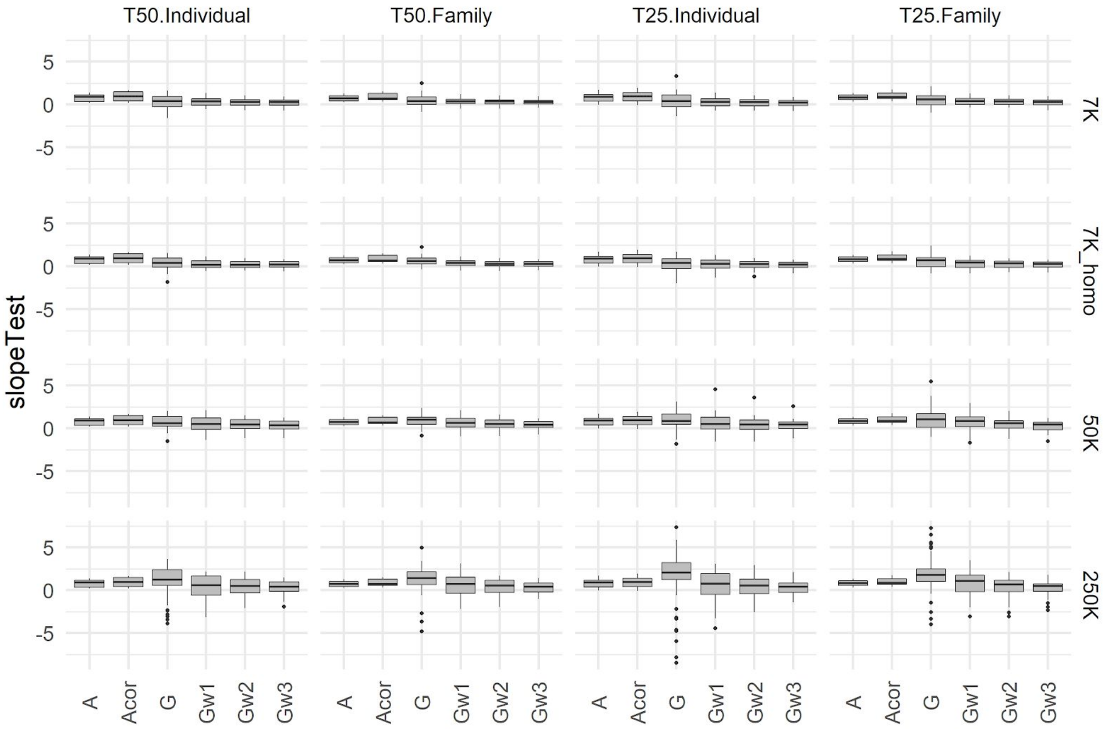

Regression slopes between phenotypes and estimated breeding value in the Test set population. Each boxplot represented accuracy values for pooled traits according to relationship matrices used, and were classified by cross-validation strategy in columns and by the number of markers in rows.

Figure S12

Comparison of Spearman (green) and Pearson (purple) correlations between phenotypes and estimated breeding values for the seven traits in the validation set (T50 individual sampling strategy), for different relationship matrices (within panels abscissas) and SNP densities (across panel columns). Across panel rows represent the tier used for the calculation of correlations: 0-5\% for the 5\% best individuals; 5-10\%, between the 5\% and 10\% best individuals; 10-50\%, between the 10\% and 50\% best individuals, and 100% for the whole validation set.

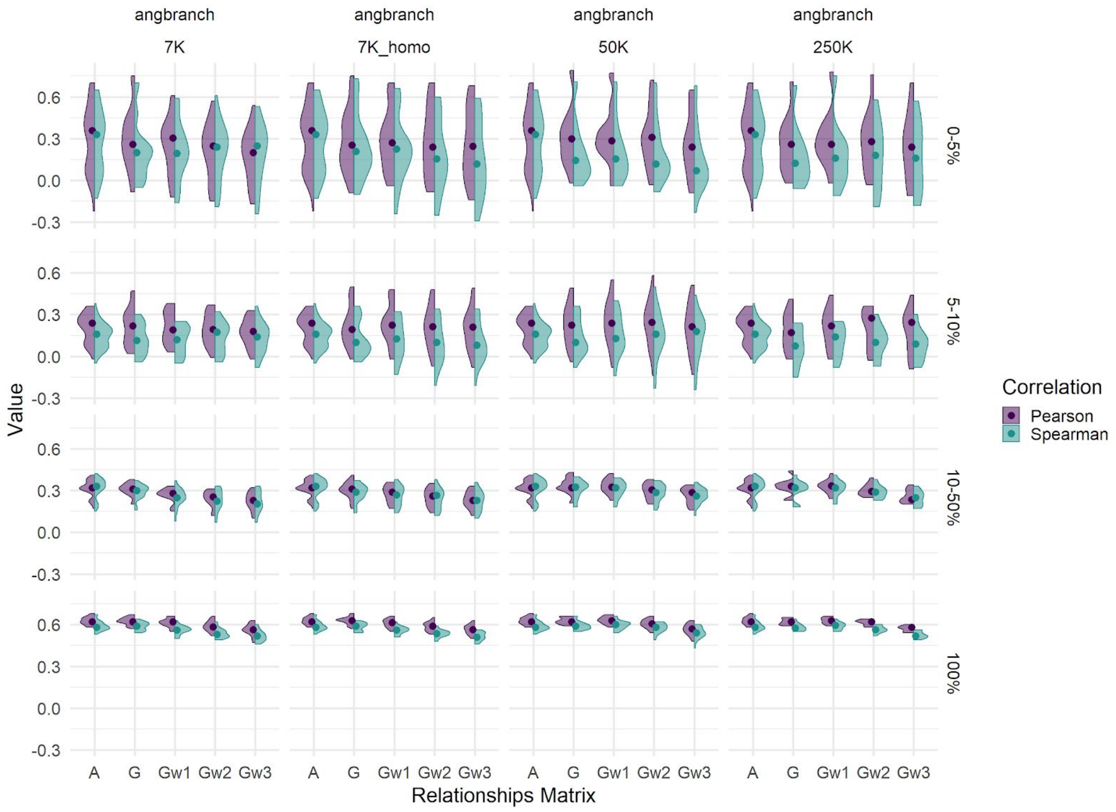

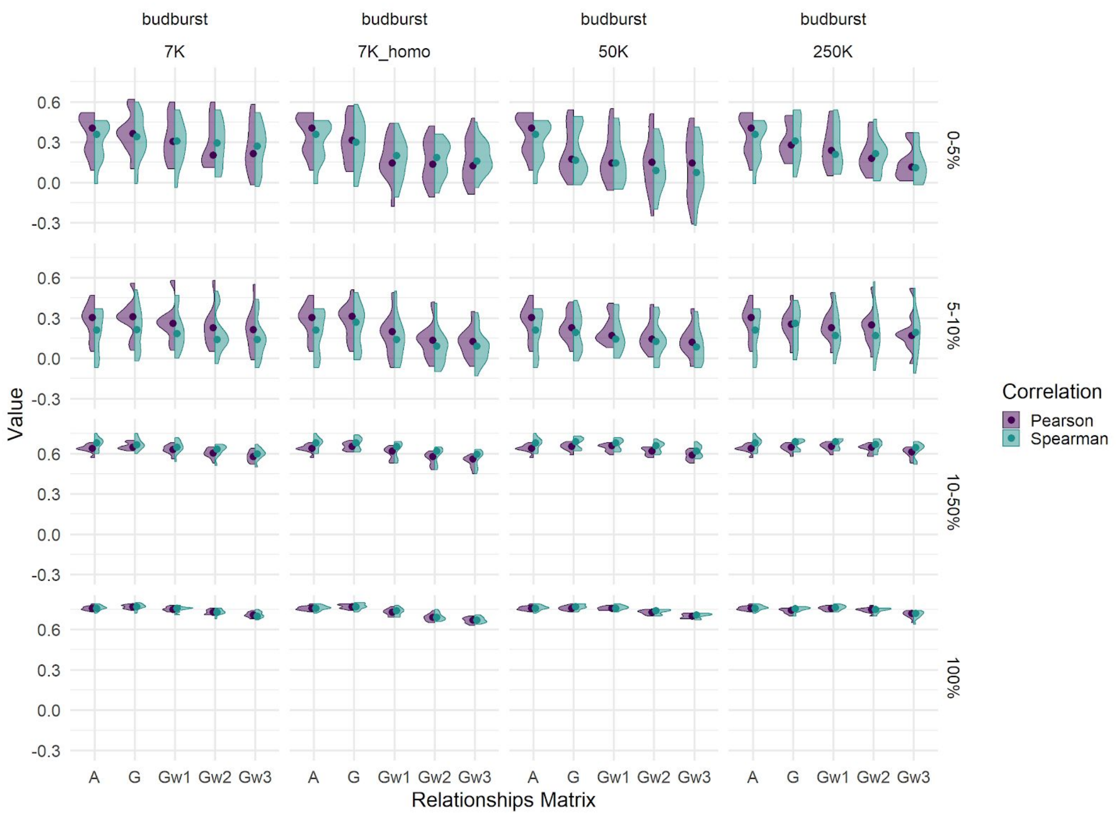
budburst 7K
budburst 7K_homo
budburst 50K
budburst 250K
0-5%
5-10%
10-50%
100%
Value
Relationships Matrix
A G Gw1 Gw2 Gw3
Correlation
Pearson
Spearman

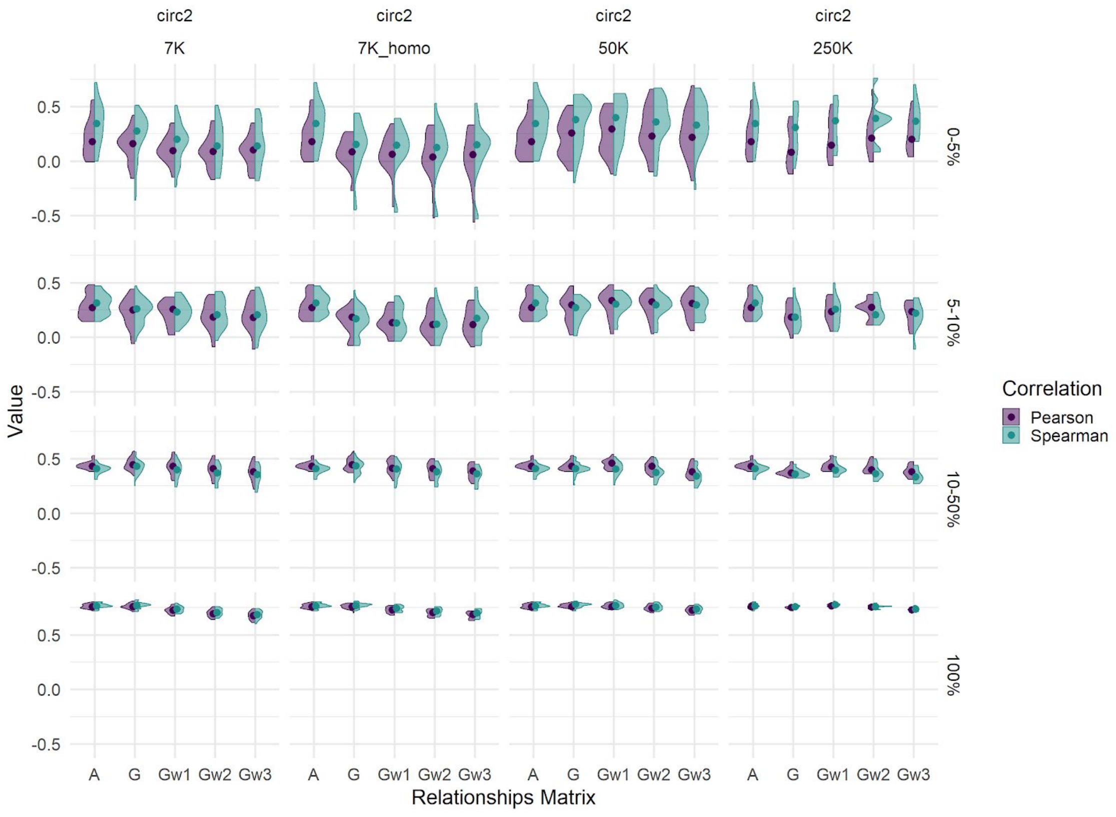
circ2 7K
circ2 7K_homo
circ2 50K
circ2 250K
0-5%
5-10%
10-50%
100%
Value
Relationships Matrix
A G Gw1 Gw2 Gw3
Correlation
Pearson
Spearman

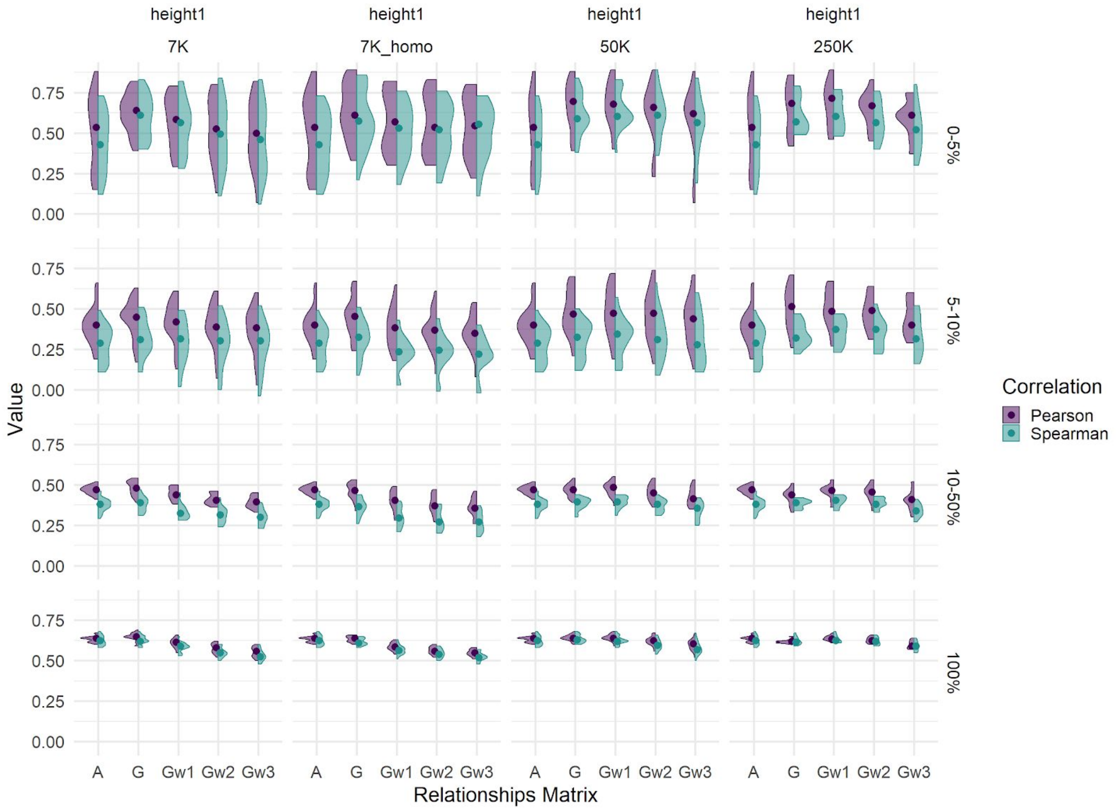

height1
7K
7K_homo
50K
250K
0-5%
5-10%
10-50%
100%
Value
Relationships Matrix
A
G
Gw1
Gw2
Gw3
Correlation
Pearson
Spearman

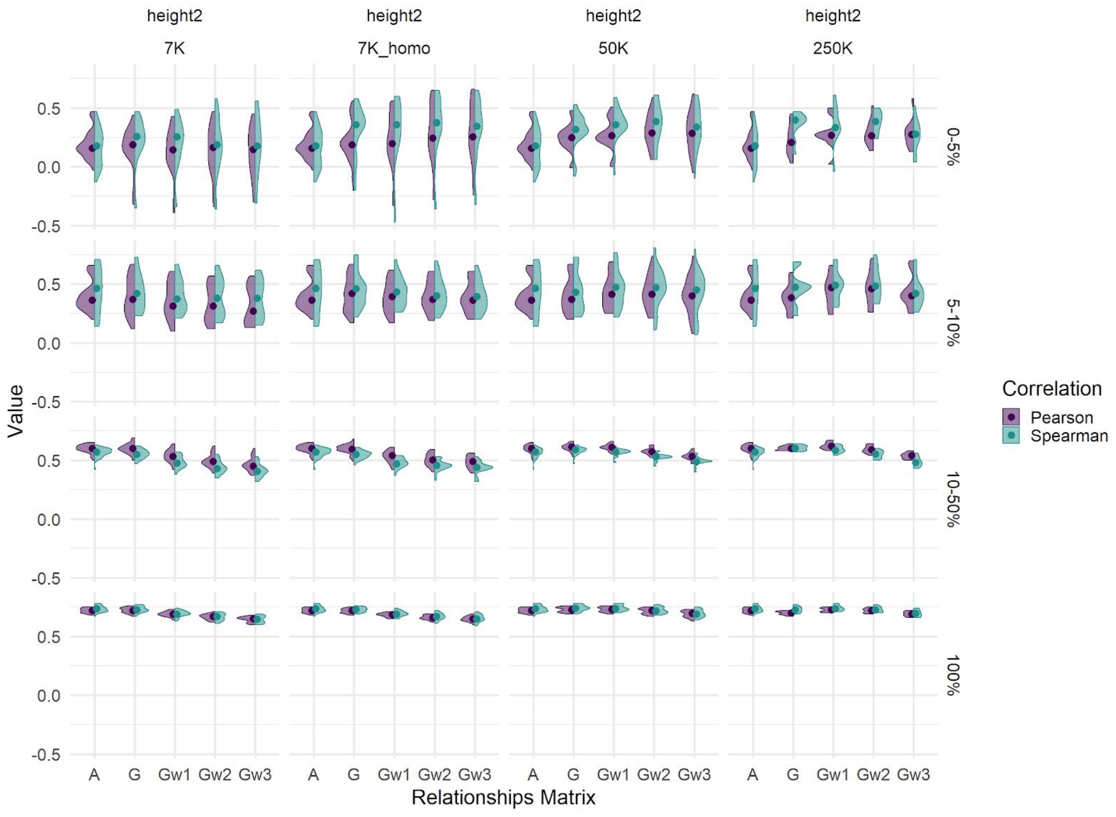

height2
7K
7K_homo
50K
250K
0-5%
5-10%
10-50%
100%
Value
Relationships Matrix
A
G
Gw1
Gw2
Gw3
Correlation
Pearson
Spearman

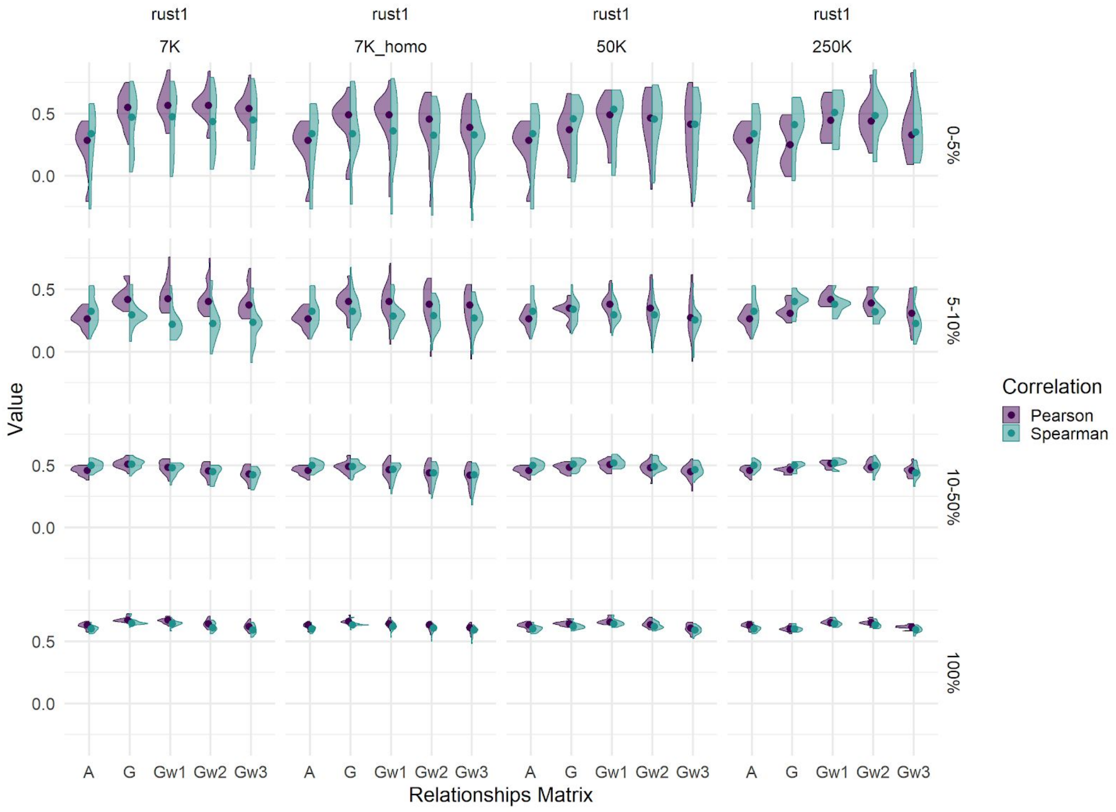

rust1
7K
7K_homo
50K
250K
0-5%
5-10%
10-50%
100%
Value
0.5
0.0
A
G
Gw1
Gw2
Gw3
Relationships Matrix
Correlation
Pearson
Spearman

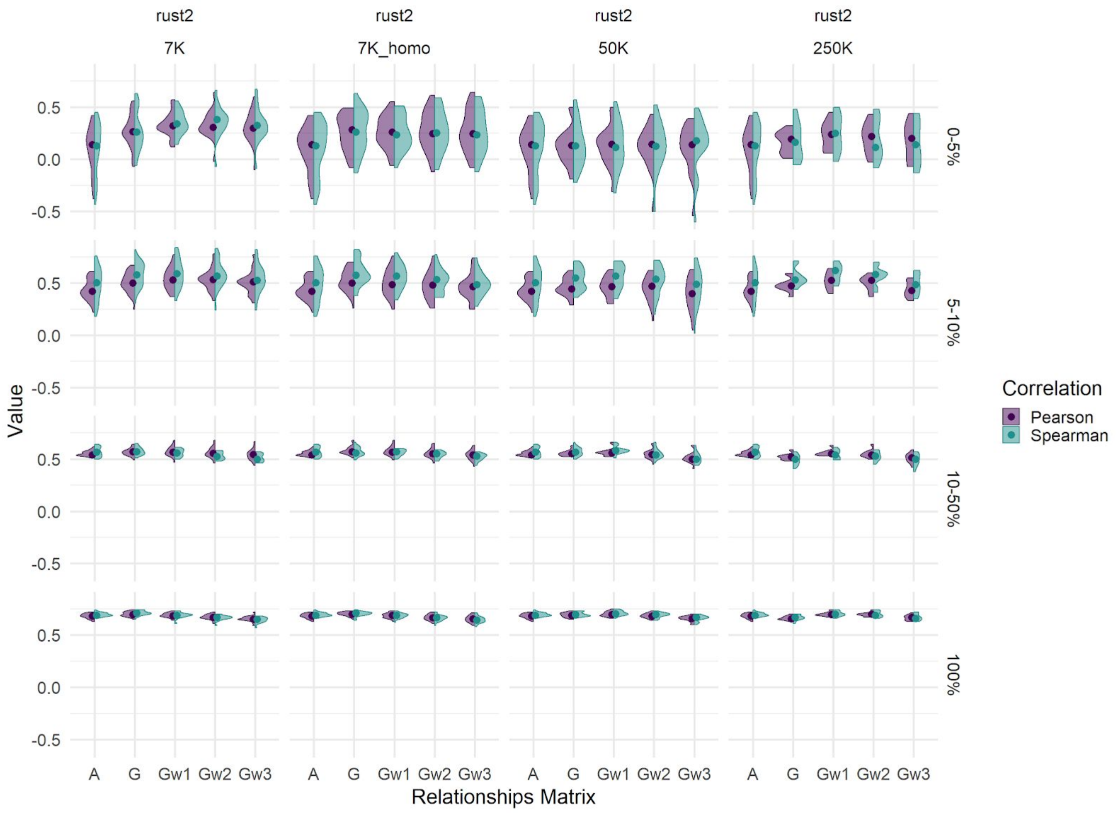

rust2
7K
7K_homo
50K
250K
0-5%
5-10%
10-50%
100%
Value
0.5
0.0
-0.5
A
G
Gw1
Gw2
Gw3
Relationships Matrix
Correlation
Pearson
Spearman

Figure S13

Comparison of Spearman (green) and Pearson (purple) correlations between phenotypes and estimated breeding values for height1 and rust1 in the Test set (T50 individual sampling strategy), for different relationship matrices (within panels abscissas) and SNP densities (across panel columns). Across panel rows represent the tier used for the calculation of correlations: 0-5\% for the 5\% best individuals; 5-10\%, between the 5\% and 10\% best individuals; 10-50\%, between the 10\% and 50\% best individuals, and 100% for the whole Test set.

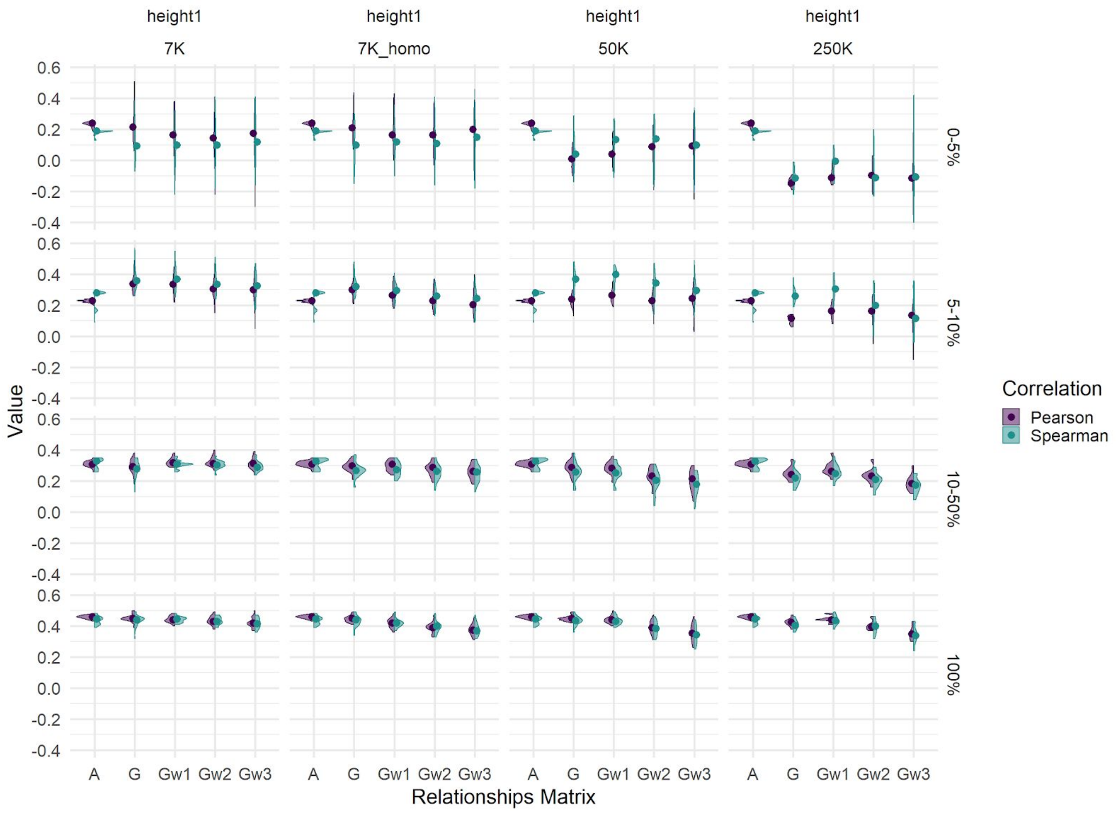

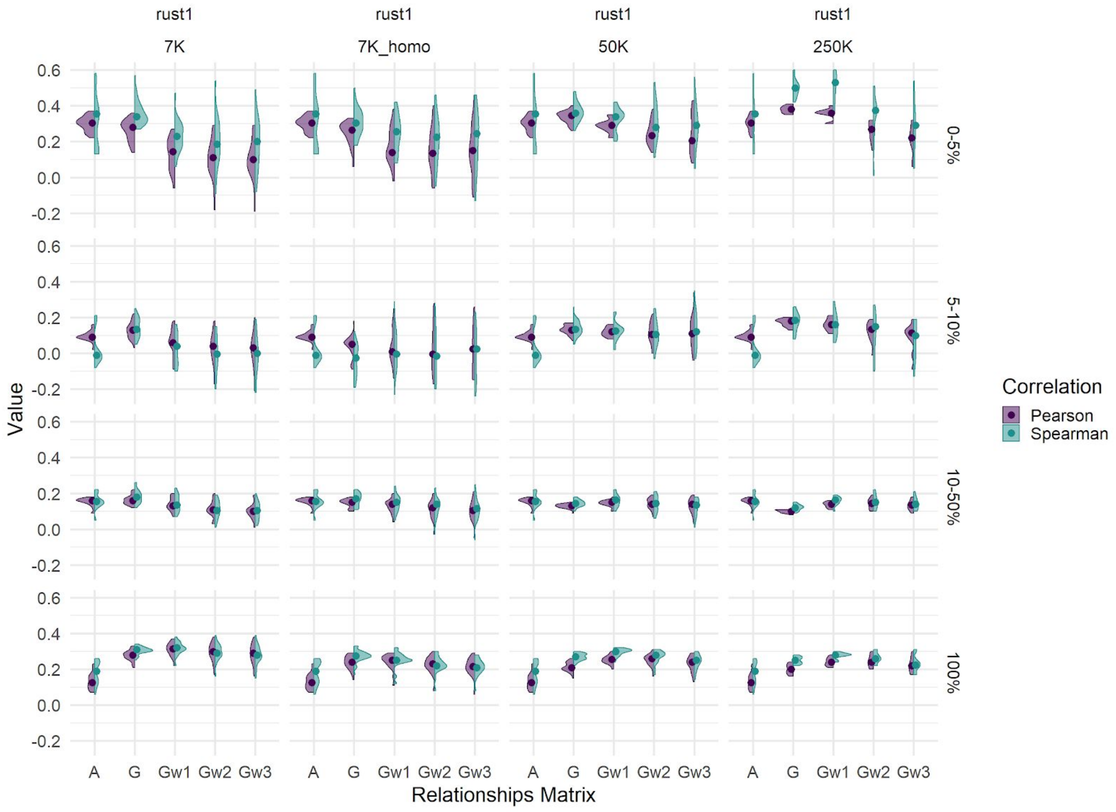

rust1
7K
rust1
7K_homo
rust1
50K
rust1
250K
0-5%
5-10%
10-50%
100%
Value
0.6
0.4
0.2
0.0
-0.2
A
G
Gw1
Gw2
Gw3
Relationships Matrix
Correlation
Pearson
Spearman

Figure S14

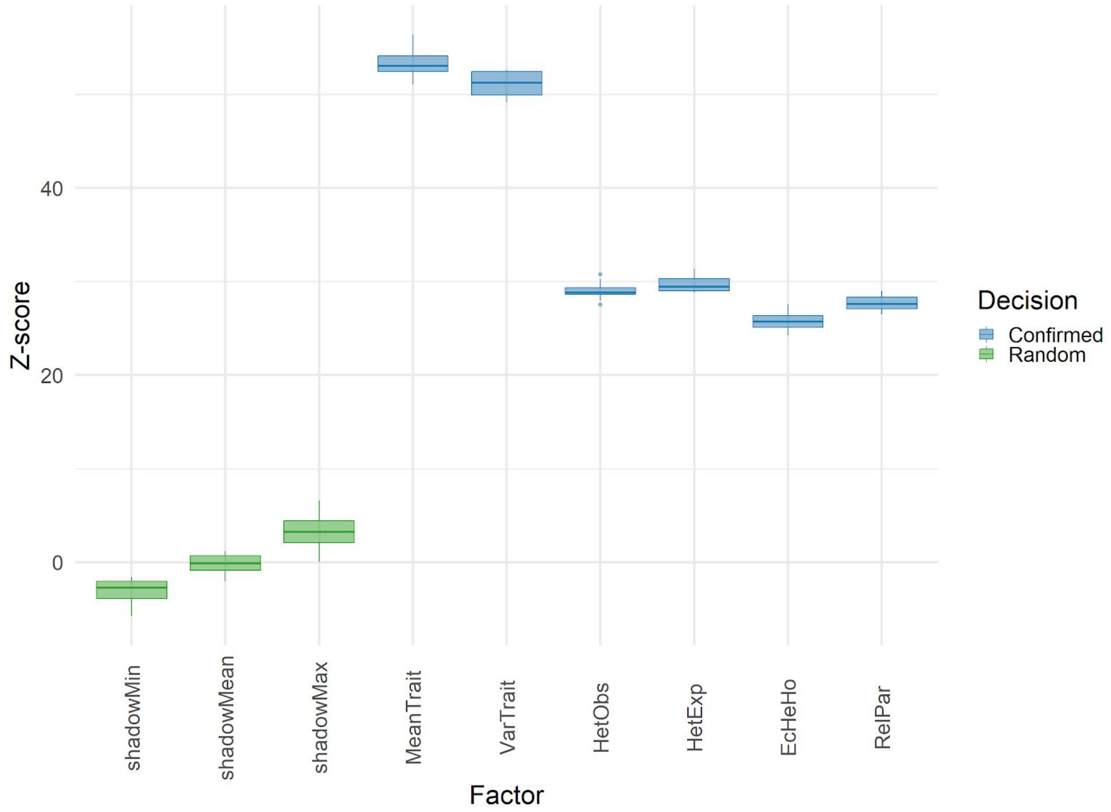

Importance (Z-score) for each features estimated with Boruta algorithm to explain the variability of prediction within family. Boruta shadow features were ShadowMin, ShadowMean and ShadowMax. The test factors were the trait family's mean (MeanTrait), the trait family's variance (VarTrait), the heterozygosity observed (HetObs) in the family, the heterozygosity expected (HetExp) from the parent genotypes, the difference between the two (EcHeHo) and the genomic relationship between the parent (RelPar) estimated with the 7K SNP set. Algorithm decision for each factor based on the significativity of the difference between factors and the shadow features are in color: Green: Shadow features, Blue: Confirmed and Red Rejected.
